# *ADAMTS12*, a new candidate gene for pediatric stroke

**DOI:** 10.1371/journal.pone.0237928

**Published:** 2020-08-20

**Authors:** Anika Witten, Frank Rühle, Marlous de Witt, Andrei Barysenka, Michael Stach, Ralf Junker, Ulrike Nowak-Göttl, Monika Stoll

**Affiliations:** 1 Institute of Human Genetics, Genetic Epidemiology, University of Münster, Münster, Germany; 2 IT Service Centre, University Hospital of Münster, Münster, Germany; 3 Institute of Clinical Chemistry, University Hospital Schleswig-Holstein, Kiel, Germany; 4 Department of Biochemistry, Cardiovascular Research Institute Maastricht, Maastricht University, Maastricht, The Netherlands; Stellenbosch University Faculty of Medicine and Health Sciences, SOUTH AFRICA

## Abstract

We recently reported a family-based genome wide association study (GWAS) for pediatric stroke pointing our attention to two significantly associated genes of the ADAMTS (a disintegrin and metalloproteinase with thrombospondin motifs) gene family *ADAMTS2* (rs469568, *p* = 8x10^-6^) and *ADAMTS12* (rs1364044, *p* = 2.9x10^-6^). To further investigate these candidate genes, we applied a targeted resequencing approach on 48 discordant sib-pairs for pediatric stroke followed by genotyping of the detected non-synonymous variants in the full cohort of 270 offspring trios and subsequent fine mapping analysis. We identified eight non-synonymous SNPs in *ADAMTS2* and six in *ADAMTS12* potentially influencing the respective protein function. These variants were genotyped within a cohort of 270 affected offspring trios, association analysis revealed the *ADAMTS12* variant rs77581578 to be significantly under-transmitted (*p* = 6.26x10^-3^) to pediatric stroke patients. The finding was validated in a pediatric venous thromboembolism (VTE) cohort of 189 affected trios. Subsequent haplotype analysis of *ADAMTS12* detected a significantly associated haplotype comprising the originally identified GWAS variant. Several ADAMTS genes such as *ADAMTS13* are involved in thromboembolic disease process. Here, we provide further evidence for *ADAMTS12* to likely play a role in pediatric stroke. Further functional studies are warranted to assess the functional role of ADAMTS12 in the pathogenesis of stroke.

## Introduction

Pediatric stroke happens with an incidence of 2.6–6.4 per 100 000 children per year and is still one of the top 10 causes of death in children [1, 2]. Extracellular matrix components like members of the ADAMTS (a disintegrin and metalloproteinase with thrombospondin motifs) gene family, primarily *ADAMTS13*, in combination with misbalanced coagulation signals appear to play an important role in pediatric stroke etiology after postnatal vascular injuries [3, 4].

Recently, we have reported results of a family-based genome wide association study (GWAS) for pediatric stroke pointing our attention to *ADAMTS2* and *ADAMTS12* [1]. *ADAMTS2* (rs469568, *p* = 8x10^-6^) and *ADAMTS12* (rs1364044, *p* = 2.9x10^-6^) were both highly associated with pediatric stroke. Furthermore, an association of rs1364044 was described having a protective effect in cerebral aneurysms (CA) pathogenesis in a case control study (OR 0.65) [5]. *ADAMTS2* is a pro-collagen N-propeptidase and known to be involved in pro-collagen processing and has properties of angiogenesis inhibition and interfering with tumor growth. The protein is involved in the innate immune system, playing a role in calming inflammation and tissue repair [6]. *ADAMTS12*, is classified as proteinase of the cartilage oligomeric matrix protein (COMP), it is inducing neutrophil apoptosis in mice, and silenced *ADAMTS12* genes were found in human tumor cells. As *ADAMTS2*, the protein is showing antiangiogenic characteristics [7].

To further investigate the molecular background and function in pediatric stroke of the two genes we conducted a targeted resequencing approach on 48 discordant sib-pairs and a subsequent fine mapping analysis in the full cohort of 270 affected trios.

## Material and methods

### Subjects

The study was performed in accordance with the Helsinki Declaration of 1975 and was approved by the ethical standards of the medical ethics committee, University of Münster (2008-161-f-S) and of the University Clinics Schleswig-Holstein (UKSH) (B 304/16), Germany. Written confirmed consent was obtained from all participants or their parents. Enrollment and genotyping of affected pediatric stroke family trios, probands selection and characteristics of 48 independent pediatric probands and 48 unaffected siblings has been described by Arning et al. and Stoll et al. [1, 3]. From the same catchment area, 189 offspring trios for pediatric venous thromboembolism (VTE) enrolled from neonates and children with confirmed diagnosis of VTE not older than 18 years at onset and having available nonaffected biological brothers, sisters and parents were utilized for the pseudo-replication study and are described in detail by Rühle et al. [8].

### Targeted resequencing and data analysis

The resequencing approach and subsequent next generation sequencing (NGS) data analysis was the same described in detail in our previous study [3]. In brief, 1μg genomic DNA isolated from EDTA blood samples was applied to perform a TruSeq DNA Sample Prep (Illumina) followed by target capture using the NimbleGen SeqCap EZ Choice Library Preparation System (Roche). The resulting libraries were pooled equimolar and sequenced in a paired-end mode (100 cycles) using the TruSeq PE Cluster Kit v3 and TruSeq SBS chemistry v2 (Illumina) on a HiScanSQ sequencing system (Illumina). After quality control, on average of 382,245 read pairs per sample were retained for further analysis. High-quality reads were mapped to the reference genome (hg19) using BWA software (ver. 0.6.2) [9]. Over 99% of the reads were mapped resulting in a mean coverage of the target region of about 227. Reads were realigned around indels and quality score recalibration was performed using GATK 2.0 [10]. GATK tool UnifiedGenotyper was used to produce a raw variant set (SNPs, short indels). In order to filter false positive variants from this set, “hard filtering” of the variants was performed [11, 12]. The Protein Variation Effect Analyzer (PROVEAN) web tool was used to assign Sorting Intolerant From Tolerant (SIFT) scores for the (non-synonymous) variants [13]. Combined Annotation Dependent Depletion (CADD) scores were looked up on https://cadd.gs.washington.edu/. Sequencing data is deposited to the Sequence Read Archive (SRA) and accessible with the following link: http://www.ncbi.nlm.nih.gov/bioproject/647652.

### Association and haplotype testing

For association analysis, a family-based approach was implemented: A custom software based on the Sibship-Disequilibrium-Test (SDT) allows to test for association and linkage and does not require parental genotypes [14]. Rare-variant association analysis was performed on SNPs with an minor allele frequency (MAF) of ≤0.02 without ambiguous genotypes (NoCalls) using a C-alpha test statistic [15]; both tests are implemented in PLINK/SEQ (http://atgu.mgh.harvard.edu/plinkseq). Empirical *p*-values were estimated using 10000 permutations per gene. SNP haplotypes for the 48 discordant sib-pairs were tested by FBAT [16] using a sliding window approach ranging from 13 (*ADAMTS12*) to 15 (*ADAMTS2*) SNPs, the optimal window size was defined by the minimal *p*-value corrected for 1 million permutations. Median joining (MJ) networks were drawn by Network 4.6.1.1 (http://fluxus-engineering.com).

### Genotyping and association analyses

Genotyping in 270 affected offspring trios of 15 SNPs selected by sequencing was carried out with TaqMan SNP Genotyping Assays (Life Technologies) as described in Stoll et al. [3]. The missing-genotype rate for all SNPs was < 1%, Hardy-Weinberg-Equilibrium (HWE) for founders was tested using an exact test as implemented in PLINK software package version 1.07 [17] and was met for all. The total calling rate was 94% (non-synonymous SNPs *ADAMTS2*), 98.6% (non-synonymous SNPs *ADAMTS12*) and 95.6% (tagging SNPs). Association of pediatric stroke affection status was calculated using the Transmission Disequilibrium Test (TDT) as implement in PLINK without adjustment for covariates. The Bonferroni-method was used for multiple testing correction. Association of haplotypes was calculated using the family trio association test implemented in Haploview 4.2 [18] defining blocks by the 4 gamete rule, examine haplotypes >5% and applying a HW *p*-value cutoff of 0.05. Pseudo-replication in 189 pediatric VTE family trios for rs77581578 was performed using the same settings.

A *p*-value cutoff of 0.05 was applied for all statistical test. The overall study design and workflow is shown in S1 Fig.

## Results

### Targeted resequencing of *ADAMTS2* and *ADAMTS12* in 48 discordant sib-pairs

Based on our association findings in the previous GWA study [1] for *ADAMTS2* and *ADAMTS12* we selected two candidate regions for subsequent NGS based on linkage disequilibrium (LD) information using the postgwas package [19]. For *ADAMTS2* a 275 kbp region (Chr5, 178,525,000–178,800,000) and for *ADAMTS12* a 415 kbp region (Chr5, 33,510,000–33,925,000) was selected for subsequent target enrichment and paired-end sequencing in 48 children affected with pediatric stroke and their unaffected siblings. After data processing, we were able to identify 1201 SNPs and indels in the region of *ADAMTS2* and 1435 for *ADAMTS12* (see S1 and S2 Tables). Out of these variants, eight SNPs (*ADAMTS2*) and six SNPs (*ADAMTS12*) were annotated as non-synonymous in the coding regions in the two genes and represent interesting new disease candidates.

For association analysis of the identified variants a Sibship Disequilibrium Test (SDT) was used, no significant *p*-values were detected likely due to power restrictions (see S3 Table).

### Testing for rare variants within *ADAMTS2* and *ADAMTS12*

Our targeted resequencing approach identified 287 (*ADAMTS2*) and 406 (*ADAMTS12*) rare variants (MAF≤0.02) in the region of the two genes not considered for the SDT association analysis and we applied a C-alpha test statistic to test for an accumulation of rare variants in the affected children. This collapsing test yielded a significant association with a combined *p*-value of *p* = 3.8x10^-2^ for *ADAMTS2* and *p* = 4.5 x10^-2^ for *ADAMTS12*.

### Median joining networks

**Fig 1:**
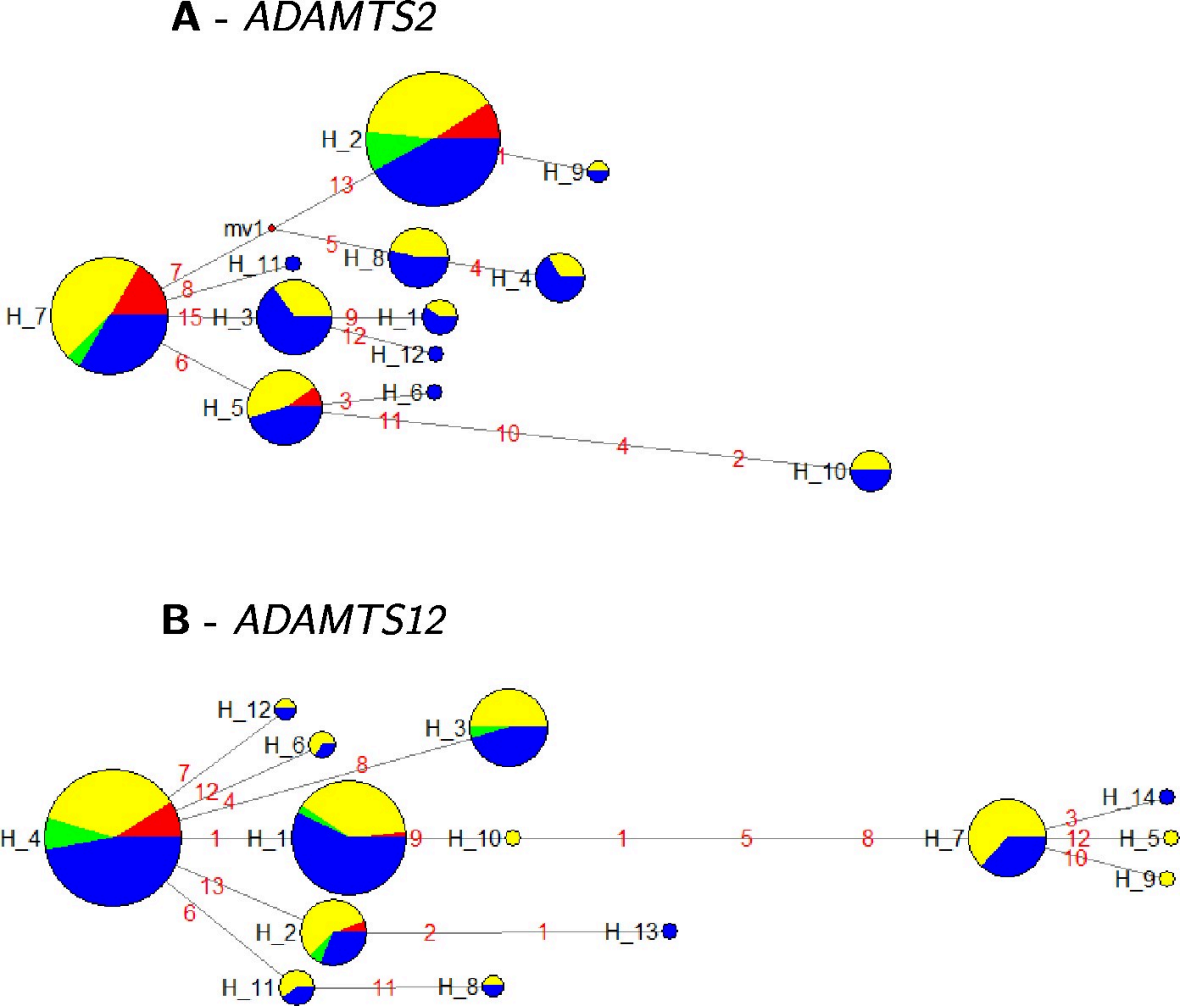
Median joining networks for *ADAMTS2* and *ADAMTS12*. Median joining networks of our detected haplotypes from *ADAMTS2* (A) and *ADAMTS12* (B) genes with indicated domain positions. Node size indicates the quantity of probands showing the haplotype either hetero- or homozygous. Color code represents the distribution of haplotypes in cases and controls (case/heterozygous = yellow, case/homozygous = red, control/heterozygous = blue, control/ homozygous = green). Evolutionary distance between haplotypes is weighted for allele frequencies and illustrated by edge length.

To assess the genetic structure of our two candidate genes *ADAMTS2* and *ADAMTS12* we performed a haplotype analysis based on our targeted resequencing data of the 48 discordant sib-pairs and visualized the results applying median joining (MJ) networks (Fig 1). The MJ networks enable to infer the evolutionary relationship of the haplotypes given as the length of the edges. The ancestral *ADAMTS2* haplotype H_7 showed the strongest association signal (*p* = 9.2x10^-5^). The *p*-value for the most strongly associated haplotype for *ADAMTS12* (H_1) was *p* = 5.2x10^-4^. Both haplotypes are located in the propeptide domain of the respective ADAMTS gene and point on potential functional changes fixed in our study population.

### *ADAMTS2* and *ADAMTS12* non-synonymous variant validation in 270 family trios

The non-synonymous called variants identified in our applied NGS approach on 48 discordant sib-pairs for *ADAMTS2* and *ADAMTS12* and not implemented on the previously used array for genome-wide genotyping [1] (rs1054480, rs398829 *ADAMTS2*; rs25754, rs3813474 *ADAMTS12*) were forwarded to validation in the full cohort of 270 affected offspring trios for pediatric stroke already applied in our former GWAS study [1] and subsequent family-association testing using TDT (see Table 1). While no significant association was found for variants residing in *ADAMTS2*, we observed the *ADAMTS12* variant rs77581578 to be significantly under-transmitted (*p* = 6.3x10^-3^) to pediatric stroke patients. This protective variant resides within a thrombospondin 1 domain of *ADAMTS12* and leads to replacement of proline by threonine at amino acid position 1329 (transcript ID NP_001311441). This change is predicted as damaging to the protein according to the respective SIFT score of 0.029 and a CADD score of 23.3 and potentially influences the protein´s function and adhesion properties.

**Table 1:** Family-association testing results of coding *ADAMTS2* and *ADAMTS12* variants.

SNP	position	A1	A2	MAF	T	U	OR (95% C.I.)	Chi^2^	*p*	Bonf
*ADAMTS2*										
rs35445112	178,555,097	T	C	0.026	11	17	0.65 (0.3–1.38)	1.29	0.256	1
rs35372714	178,563,002	T	C	0.003	1	1	1 (0.06–15.99)	0	1	1
-	178,634,681	T	C	0.003	2	1	2 (0.18–22.06)	0.33	0.563	1
rs11750821	178,634,683	T	C	0.107	47	57	0.82 (0.56–1.21)	0.96	0.327	1
rs59567206	178,634,704	C	T	0.286	131	128	1.02 (0.8–1.31)	0.035	0.852	1
*ADAMTS12*										
rs61753559	33,546,207	C	T	0.005	3	2	1.5 (0.25–8.98)	0.20	0.655	1
rs112196098	33,549,350	G	C	0.045	21	25	0.84 (0.47–1.5)	0.35	0.555	1
**rs77581578**	33,549,374	T	G	0.009	0	10	0	10	2x10^-3^	6.3x10^-3^
rs140486982	33,891,894	A	G	0.006	2	3	0.67 (0.11–3.99)	0.20	0.655	1

Family-association testing of nine non-synonymous variants of *ADAMTS2* and *ADAMTS12*. Based on our targeted resequencing result for *ADAMTS2* and *ADAMTS12* variants were selected and forwarded to subsequent genotyping in the full cohort of 270 affected offspring trios for pediatric stroke.

(position = genomic location on chromosome 5, A1 = minor allele, A2 = major allele, MAF = minor allele frequency, T = transmitted minor allele count, U = untransmitted allele count, OR = odds ratio, C.I. = confidence interval, *p* = TDT *p*-value, Bonf = Bonferroni corrected *p*-value).

A separate cohort of 189 affected child trios suffering from venous thromboembolism (VTE) was used for pseudo-replication of the variant rs77581578 and subsequent TDT analysis revealed a significant validation signal in the replication cohort of *p* = 0.025 (Bonferroni corrected; T = 0, U = 5).

### Fine mapping of *ADAMTS12*

**Fig 2:**
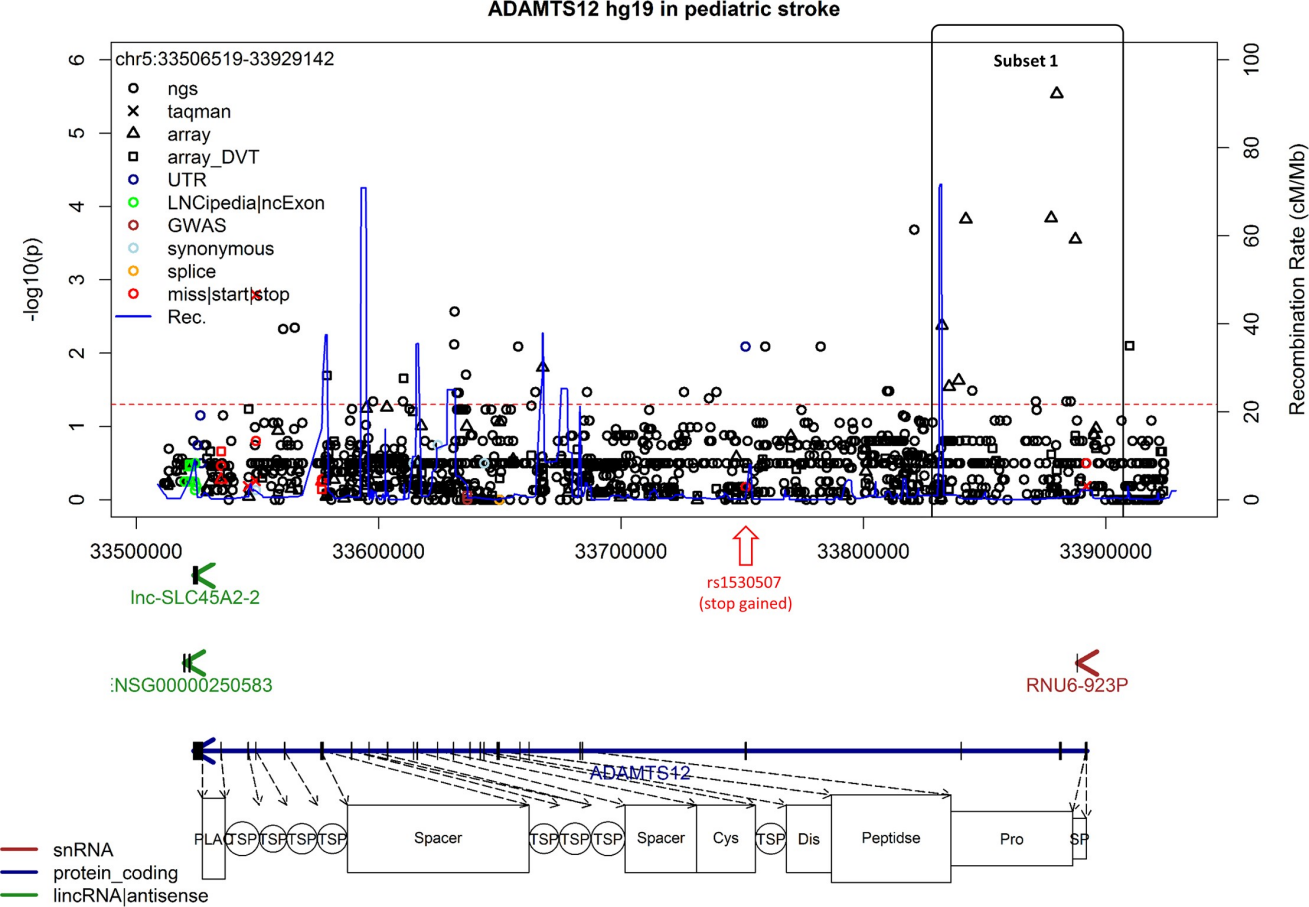
*ADAMTS12* regional plot. Regional plot of *ADAMTS12* showing the dominant haplotype block and association of the detected SNPs marker with pediatric stroke.

Due to the results of the single marker analysis above we decided to focus on *ADAMTS12* and to perform a fine mapping analysis. A dominant LD block was noticed (subset 1, see Fig 2) for *ADAMTS12*. Fine mapping was performed in this subset because it accumulates several marginal GWAS association results and is also carrying the strongest GWAS association signal (rs1364044). Beside rs1364044, four intronic haplotype tagging SNPs were selected within this subset for genotyping in the full cohort of 270 offspring trios. As quality control for the genotyping data an additional SNP carrying the same information as rs1364044 (rs7443937) was selected for TaqMan genotyping (see Table 2). A further promising variant, rs1530507 detected by our targeted resequencing approach, was introduced to the genotyping step due to its stop gained function. None of these variants except for rs7443937 (*p* = 3.1x10^-3^) showed a significant association with pediatric stroke using a single marker TDT analysis.

**Table 2:** Single marker association analysis of *ADAMTS12*.

subset	SNP ID	A1	A2	MAF	T	U	OR (95% C.I.)	*p*	Bonf
1	rs114803348	A	C	0.22	81	108	0.75 (0.56–1.00)	0.049	0.73
1	rs17501324	T	C	0.08	47	36	1.458 (0.85–2.01)	0.227	1
1	rs139318738	T	C	0.05	28	24	1.17 (0.68–2.01)	0.579	1
1	rs75989721	G	A	0.29	101	117	0.86 (0.66–1.13)	0.279	1
1	**rs7443937**	C	T	0.38	153	98	1.56 (1.21–2.01)	5.2x10^-4^	3.1x10^-3^
	rs1530507	A	T	0.27	79	70	1.13 (0.82–1.56)	0.461	1

Single marker association analysis for the tagging SNPs in the two subsets of *ADAMTS12* for subsequent genotyping in the full cohort of 270 affected offspring trios for pediatric stroke.

(A1 = minor allele, A2 = major allele, MAF = minor allele frequency, T = transmitted minor allele count, U = untransmitted allele count, OR = odds ratio, C.I. = confidence interval, *p* = TDT *p*-value, Bonf = Bonferroni corrected *p*-value).

The genotype data was utilized for LD and haplotype analysis and it was possible to define five haplotypes in the full cohort that occur with a frequency greater than 5% (see Table 3). Two haplotypes (CCCTC and CCCTT) having a frequency of 42% and 21% respectively were significantly associated with pediatric stroke. Haplotype CCCTC (*p* = 5x10^-4^) is under-transmitted from parents to children and the corresponding haplotype CCCTT (*p* = 6.6x10^-5^) over-transmitted.

**Table 3:** Haplotype association results for *ADAMTS12*.

Haplotype	Frequency	T	U	Chi^2^	*p*
CCCTC	0.42	104.2	161.2	12.26	5.0x10^-4^
CCCTT	0.21	121.8	67.0	15.94	6.55x10^-5^
CCACC	0.17	67.6	94.9	4.59	0.032
CTCTT	0.09	48.7	34.8	2.33	0.127
CCCCC	0.07	40.9	29.4	1.89	0.69

Haplotype association results for *ADAMTS12*, Subset1. Association analysis was performed using a TDT test (given *p*-value).

(T = transmitted minor allele count, U = untransmitted allele count, *p* = TDT *p*-value).

## Discussion

Here we report the identification and validation of several non-synonymous *ADAMTS2* and *ADAMTS12* variants to further prove the role of the two members of the ADAMTS gene family in the disease etiology of pediatric stroke. *ADAMTS2* (rs469568, *p* = 8x10^-6^) and *ADAMTS12* (rs1364044, *p* = 2.9x10^-6^) were first associated with the occurrence of pediatric stroke in a family-based GWAS published by our group [1]. To our knowledge, this comprehensive cohort of 270 families affected with pediatric stroke is unique and offers the outstanding possibility to characterize the genetic background of the disease and to identify the respective underlying molecular mechanisms without any environmental risk factors for stroke in adults such as smoking or obesity. Interestingly, the *ADAMTS12* variant rs1364044 has recently shown to be associated with the pathogenesis of cerebral aneurysms emphasizing the role of the gene in stroke related phenotypes [5].

Therefore, we conducted a NGS based targeted resequencing approach to trace our previous GWAS findings, where we first sequenced the surrounding genomic regions based on LD information of the *ADAMTS2* and *ADAMTS12* GWAS hits in 48 discordant sib-pairs. We were able to detect several synonymous and non-synonymous variants within the coding regions of the two genes, which strengthen their role as interesting novel candidate genes for pediatric stroke. Although we applied a discordant sib-pair approach and SDT testing, which should provide an advantage to achieve sufficient statistical power while testing a reasonable sample size [20] due to sequencing costs, we were not able to achieve statistical significance for any of these variants.

We are aware that, despite the discordant sib-pair study design, resequencing of only 96 individual samples is a limitation to test for rare variants. Nevertheless, we applied a gene based collapsing test (C-alpha) on the identified rare variants. We were able to support our previous association findings for the two genes by detecting a significant C-alpha *p*-value of *p* = 3.8x10^-2^ for *ADAMTS2* and *p* = 4.5x10^-2^ for *ADAMTS12*. Based on our resequencing data, we applied haplotype MJ network analysis revealing first evidence on the contribution of the propeptide domain of both genes in disease etiology which is discussed later and an evolutionary fixation on our study population.

In contrast to our SDT association findings we were able to validate the non-synonymous *ADAMTS12* variant rs77581578 (TDT *p* = 6.3x10^-3^) in the full cohort of 270 affected offspring trios for pediatric stroke. The thrombospondin (TSP)-1 domain variant is predicted as influencing protein function and protein´s adhesion properties, this finding is in accordance with previous reports on ADAMTS gene family members such as *ADAMTS13* linking disease associated variants of the thrombospondin domain and protein function in pediatric stroke [3]. An independent replication of our association finding in another study population would strengthen the impact of our findings. We lack an independent replication cohort for pediatric stroke as to our knowledge; no comparable collection of father-mother-child trios is available in the scientific community. Indeed, we were in the fortunate position to have access to a study cohort for pediatric VTE recruited from the same catchment area. Pediatric stroke and VTE share a similar genetic background as well as a comparable prevalence of prothrombotic risk factors, and—in our opinion—is the most suitable cohort for validation of our findings given that no other pediatric stroke cohort is available to conduct a true replication. Notably, the pediatric stroke and VTE share many associated clinical conditions and diseases such as neonatal infections, leukemia or sickle cell disease [21, 22]. Most notably many prothrombotic abnormalities are prominent risk factors for pediatric stroke und both diseases share known susceptibility genes as *prothrombin* or *factor V-Leiden* [22, 23]. Furthermore, risk factors differ between children and adults due to the lack of classical environmental risk factors like smoking stressing the need to replicate findings within pediatric cohorts [6]. Interestingly, we were able to pseudo-replicate the variant rs77581578 (TDT *p* = 0.025) in this cohort but we were not able to validate the stop gained variant rs1530507. Our findings point on *ADAMTS12* as a promising new candidate gene for pediatric stroke hence we focused on this gene for a subsequent fine mapping analysis to refine the identified association signal. The resulting significantly associated common haplotype is, in accordance with the initial GWAS, signal located in the in the intronic regions in-between the propeptide domain of *ADAMTS12*.

## Conclusion

Taken together, our study confirms the role of *ADAMTS12* as a promising candidate gene for pediatric stroke. Our approach has shown that the region of the associated haplotype block in *ADAMTS12* and the variant rs77581578, is of particular importance in identifying the underlying functional mechanisms in pediatric stroke. *ADAMTS12* is known to degrade cartilage oligomeric matrix protein (COMP), a non-collagenous protein in cartilage. Abnormalities in this protein usually result in skeletal dysplasia. Produced by vascular smooth muscle cells and platelets, it has also a key role in maintaining cardiovascular haemostasis and downregulating coagulation by inhibition of thrombin [24]. An increase in *ADAMTS12* activity would hypothetical lead to the degradation of COMP resulting in less inhibition of thrombin and an elevated risk for ischemic stroke. However, the *ADAMTS12*-COMP-thrombin axis has not yet been reported in the context of (pediatric) stroke. The determination of *ADAMTS12* levels in our study cohort in the future would allow to establish a causal relationship between *ADAMTS12* levels and pediatric stroke susceptibility. Nevertheless, interaction of the TSP-1 like repeats of *ADAMTS12* with the EGF-like repeat domain in COMP had been reported in absence of other domains, including the propeptide domain [25]. Therefore, the propeptide domain may only have a marginal impact on the catalytic function of *ADAMTS12*. Further functional studies are warranted to assess the role of *ADAMTS12* in disease etiology.

## Supporting information

S1 Fig Study design. Workflow and design of the study including sample sizes and methods.
(TIF)

S1 Table Sibship Disequilibrium Test (SDT) for *ADAMTS2* and *ADAMTS12* variants. Variants detected using NGS based target enrichment in the defined genomic region of *ADAMTS2*.
(DOCX)

S2 Table Sibship Disequilibrium Test (SDT) for *ADAMTS2* and *ADAMTS12* variants. Variants detected using NGS based target enrichment in the defined genomic region of *ADAMTS12*.
(DOCX)

S3 Table Sibship Disequilibrium Test (SDT) for *ADAMTS2* and *ADAMTS12* variants. Results of the SDT association analysis in 48 discordant sib-pairs for the non-synonymous variants in *ADAMTS2* and *ADAMTS12*.
(DOCX)
