# Supplementary figures and images for: ADAMTS12, a new candidate gene for pediatric stroke
Source: PLoS One. 2020 Aug 20;15(8):e0237928. doi: 10.1371/journal.pone.0237928 (PMC7446847; doi:10.1371/journal.pone.0237928)

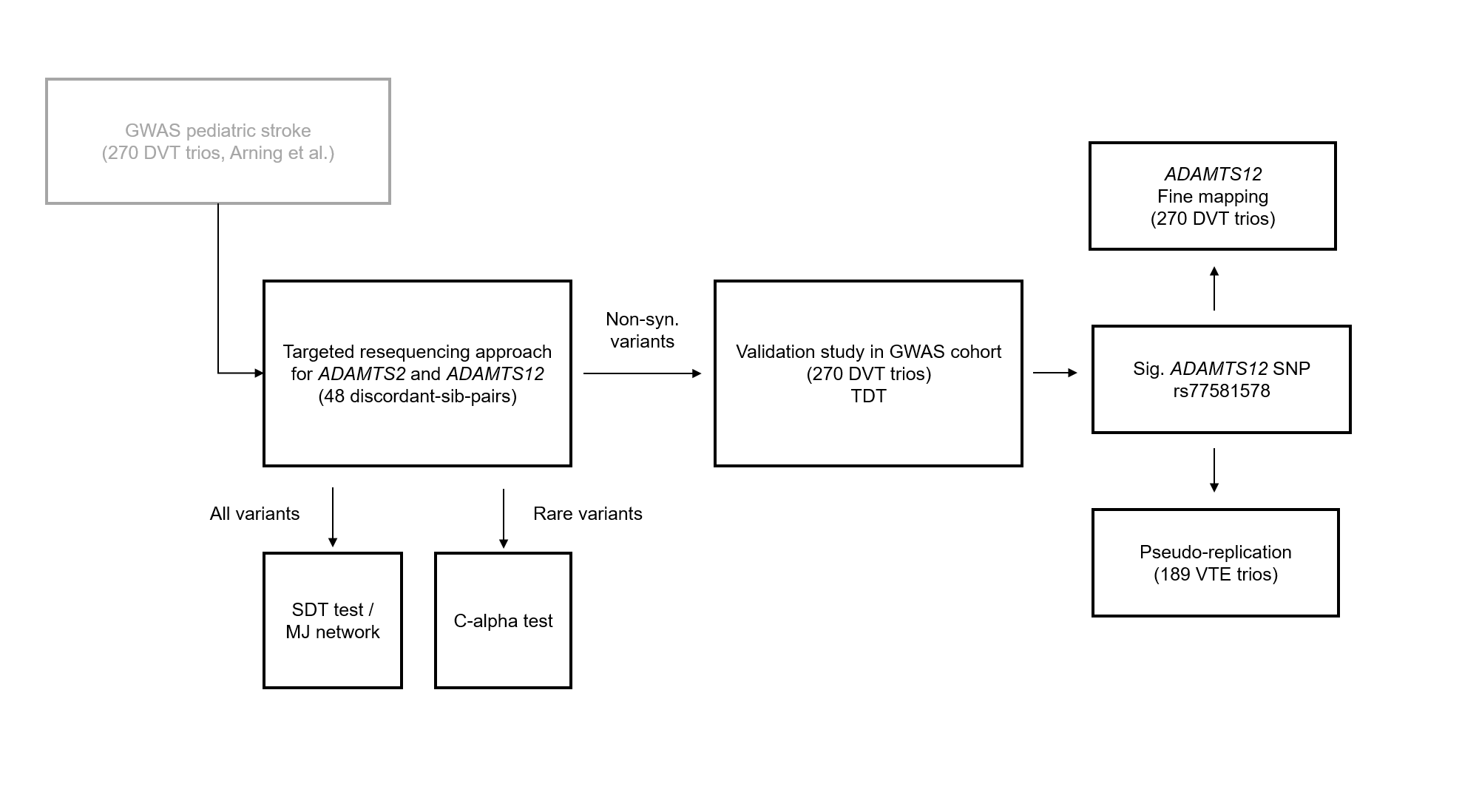

Supplement: S1 Fig — Workflow and design of the study including sample sizes and methods. (TIF) [file pone.0237928.s001.tif]
