# Supplementary material for: ADAMTS12, a new candidate gene for pediatric stroke
Source: PLoS One. 2020 Aug 20;15(8):e0237928. doi: 10.1371/journal.pone.0237928 (PMC7446847; doi:10.1371/journal.pone.0237928)
Supplement: S1 Table — Variants detected using NGS based target enrichment in the defined genomic region of ADAMTS2. (DOCX) [file pone.0237928.s002.docx]

**S1 Table. *ADAMTS2* variants.** Variants detected using NGS based target enrichment in the defined genomic region of *ADAMTS2*.

| ID | POS | REF | ALT | EFFECT | SIFT | Global minor allele frequency (all individuals) |
| --- | --- | --- | --- | --- | --- | --- |
| . | 178534037 | C | G | DOWNSTREAM | NA |  |
| rs191566574 | 178534999 | C | T | DOWNSTREAM | NA | 0.0006 |
| rs6877691 | 178535134 | T | C | DOWNSTREAM | NA | 0.4848 |
| rs138624619 | 178535603 | G | A | DOWNSTREAM | NA | 0.0082 |
| rs11750568 | 178535713 | A | G | DOWNSTREAM | NA | 0.2212 |
| rs185544283 | 178535773 | G | A | DOWNSTREAM | NA | 0.0004 |
| rs7722790 | 178536078 | T | C | DOWNSTREAM | NA | 0.1414 |
| rs7718768 | 178536089 | A | G | DOWNSTREAM | NA | 0.2386 |
| rs62393095 | 178536331 | T | C | DOWNSTREAM | NA |  |
| . | 178536445 | C | T | DOWNSTREAM | NA |  |
| rs10052941 | 178536457 | T | C | DOWNSTREAM | NA | 0.0084 |
| rs7719616 | 178536584 | A | G | DOWNSTREAM | NA | 0.2380 |
| rs7723801 | 178536634 | T | C | DOWNSTREAM | NA | 0.2338 |
| rs140016662 | 178536696 | G | A | DOWNSTREAM | NA | 0.0028 |
| rs185553060 | 178537240 | A | G | DOWNSTREAM | NA | 0.0080 |
| . | 178537278 | C | T | DOWNSTREAM | NA |  |
| . | 178537637 | T | A | DOWNSTREAM | NA |  |
| rs76423191 | 178537743 | C | T | DOWNSTREAM | NA | 0.0044 |
| rs4701047 | 178537814 | A | C | DOWNSTREAM | NA | 0.0841 |
| rs1044209 | 178537916 | A | T | UTR_3_PRIME | NA | 0.264 |
| rs1044205 | 178537917 | C | T | UTR_3_PRIME | NA | 0.264 |
| rs78886769 | 178538429 | G | A | UTR_3_PRIME | NA | 0.020 |
| . | 178538437 | T | C | UTR_3_PRIME | NA |  |
| rs3797615 | 178538472 | G | A | UTR_3_PRIME | NA | 0.346 |
| rs17666900 | 178540015 | C | A | UTR_3_PRIME | NA | 0.054 |
| rs116130524 | 178540052 | G | A | UTR_3_PRIME | NA | 0.005 |
| rs10479525 | 178540656 | T | G | UTR_3_PRIME | NA | 0.477 |
| rs1054480 | 178540975 | G | A | NON_SYNONYMOUS_CODING | 0.2 | 0.264 |
| . | 178541631 | G | A | INTRON | NA |  |
| rs4701048 | 178541941 | A | G | INTRON | NA | 0.478 |
| rs3822591 | 178542241 | G | A | INTRON | NA | 0.469 |
| rs3776807 | 178542682 | A | G | INTRON | NA | 0.304 |
| rs3776808 | 178542705 | G | A | INTRON | NA | 0.260 |
| rs3776809 | 178542706 | A | G | INTRON | NA | 0.259 |
| . | 178543124 | C | T | INTRON | NA |  |
| . | 178543155 | T | C | INTRON | NA |  |
| rs3797590 | 178544084 | A | G | INTRON | NA | 0.479 |
| rs55849753 | 178544213 | G | A | INTRON | NA | 0.240 |
| . | 178544218 | C | T | INTRON | NA |  |
| . | 178544483 | T | C | INTRON | NA |  |
| . | 178544763 | T | C | INTRON | NA |  |
| rs147765529 | 178545194 | T | C | INTRON | NA | 0.034 |
| rs4701050 | 178545385 | C | T | INTRON | NA | 0.458 |
| rs2161170 | 178546302 | C | A | INTRON | NA | 0.465 |
| . | 178546323 | C | T | INTRON | NA |  |
| rs2112319 | 178546584 | T | C | INTRON | NA | 0.028 |
| . | 178546605 | G | C | INTRON | NA |  |
| . | 178546636 | C | G | INTRON | NA |  |
| . | 178547286 | G | A | INTRON | NA |  |
| rs888762 | 178547313 | C | A | INTRON | NA | 0.204 |
| rs2288382 | 178547567 | G | A | INTRON | NA | 0.222 |
| rs2303640 | 178548514 | G | C | INTRON | NA | 0.261 |
| rs10447301 | 178549254 | G | C | INTRON | NA |  |
| rs2303641 | 178549791 | G | A | INTRON | NA | 0.252 |
| . | 178549934 | C | T | INTRON | NA |  |
| rs6871456 | 178550811 | G | C | INTRON | NA | 0.343 |
| rs10447302 | 178550869 | T | C | INTRON | NA | 0.226 |
| rs4700782 | 178551155 | A | G | INTRON | NA | 0.022 |
| rs116326067 | 178551201 | C | T | INTRON | NA | 0.006 |
| . | 178552115 | G | A | SYNONYMOUS_CODING | NA |  |
| rs116808588 | 178552466 | C | T | INTRON | NA | 0.008 |
| rs6860046 | 178552518 | A | G | INTRON | NA | 0.421 |
| rs7718671 | 178552715 | G | A | INTRON | NA | 0.447 |
| rs78566312 | 178552899 | C | T | INTRON | NA | 0.021 |
| rs2303642 | 178552956 | T | C | INTRON | NA | 0.160 |
| rs2303643 | 178553185 | G | A | INTRON | NA | 0.493 |
| rs139553138 | 178553278 | C | T | INTRON | NA | 0.003 |
| rs6601018 | 178553341 | T | C | INTRON | NA | 0.345 |
| rs62396115 | 178553402 | C | T | INTRON | NA | 0.002 |
| rs6601019 | 178553450 | A | G | INTRON | NA | 0.260 |
| rs79338928 | 178554173 | G | A | INTRON | NA | 0.005 |
| rs141293393 | 178554808 | C | T | INTRON | NA | 0.009 |
| rs2303644 | 178555045 | G | A | SYNONYMOUS_CODING | NA | 0.130 |
| rs35445112 | 178555097 | C | T | NON_SYNONYMOUS_CODING | 0.03 | 0.011 |
| rs1427845 | 178555196 | T | C | INTRON | NA | 0.016 |
| rs6601020 | 178555463 | G | A | INTRON | NA | 0.447 |
| rs144041125 | 178555640 | C | T | INTRON | NA | 0.049 |
| rs139598137 | 178555685 | C | T | INTRON | NA | 0.003 |
| rs35648285 | 178555768 | G | A | INTRON | NA | 0.227 |
| rs3776810 | 178556385 | T | C | INTRON | NA | 0.417 |
| rs140401199 | 178557107 | T | C | INTRON | NA | 0.030 |
| rs2303645 | 178557292 | C | T | INTRON | NA | 0.271 |
| . | 178557394 | T | C | INTRON | NA |  |
| . | 178557793 | C | T | INTRON | NA |  |
| rs182308629 | 178557904 | G | A | INTRON | NA | 0.000 |
| rs7722275 | 178557919 | C | T | INTRON | NA | 0.377 |
| . | 178557926 | A | G | INTRON | NA |  |
| rs7722433 | 178558025 | C | T | INTRON | NA | 0.290 |
| rs34389865 | 178558053 | G | C | INTRON | NA | 0.274 |
| rs7722465 | 178558088 | C | T | INTRON | NA | 0.291 |
| rs34211544 | 178558183 | A | G | INTRON | NA | 0.225 |
| rs7704524 | 178558194 | A | G | INTRON | NA | 0.324 |
| rs3822594 | 178559411 | C | G | INTRON | NA | 0.167 |
| . | 178559637 | C | G | INTRON | NA |  |
| . | 178559878 | G | A | SYNONYMOUS_CODING | NA |  |
| rs34204311 | 178560082 | T | A | INTRON | NA | 0.002 |
| rs139446921 | 178560090 | C | T | INTRON | NA | 0.002 |
| rs10464082 | 178561064 | G | A | INTRON | NA | 0.239 |
| rs3776811 | 178561267 | G | C | INTRON | NA | 0.435 |
| rs62396128 | 178561707 | A | G | INTRON | NA | 0.236 |
| rs1298651 | 178561777 | T | G | INTRON | NA | 0.211 |
| rs62396129 | 178562163 | A | G | INTRON | NA | 0.379 |
| rs75782830 | 178562238 | G | A | INTRON | NA | 0.131 |
| rs73344617 | 178562240 | T | C | INTRON | NA | 0.196 |
| rs3776812 | 178562389 | C | T | INTRON | NA | 0.196 |
| rs3822595 | 178562834 | C | T | INTRON | NA | 0.162 |
| rs1972715 | 178562967 | G | A | SYNONYMOUS_CODING | NA | 0.197 |
| rs35372714 | 178563002 | C | T | NON_SYNONYMOUS_CODING | 0.09 | 0.004 |
| rs1549603 | 178563125 | A | T | INTRON | NA | 0.197 |
| rs9286043 | 178563362 | T | C | INTRON | NA | 0.434 |
| rs9286044 | 178563420 | G | A | INTRON | NA | 0.196 |
| rs112334680 | 178563525 | G | C | INTRON | NA | 0.131 |
| rs10057914 | 178563709 | T | C | INTRON | NA | 0.007 |
| . | 178563710 | G | A | INTRON | NA |  |
| . | 178563737 | C | A | INTRON | NA |  |
| rs112071001 | 178563828 | G | A | INTRON | NA | 0.131 |
| rs6861255 | 178563895 | T | C | INTRON | NA | 0.465 |
| rs112158313 | 178564098 | C | T | INTRON | NA | 0.007 |
| rs9286045 | 178564971 | C | T | INTRON | NA | 0.184 |
| rs2303639 | 178565001 | C | T | INTRON | NA | 0.131 |
| rs9286046 | 178565052 | C | T | INTRON | NA | 0.197 |
| rs888759 | 178565121 | T | C | INTRON | NA | 0.469 |
| rs34447103 | 178565237 | C | T | INTRON | NA | 0.238 |
| rs147969709 | 178565375 | C | T | INTRON | NA | 0.003 |
| rs114827273 | 178565514 | C | T | INTRON | NA | 0.009 |
| rs35926292 | 178565543 | C | G | INTRON | NA | 0.234 |
| rs3822596 | 178565694 | G | C | INTRON | NA | 0.153 |
| rs3822597 | 178565708 | A | C | INTRON | NA | 0.410 |
| rs3797593 | 178565818 | G | A | INTRON | NA | 0.329 |
| rs35022365 | 178566168 | T | C | INTRON | NA | 0.005 |
| . | 178566186 | C | G | INTRON | NA |  |
| rs3797594 | 178566304 | C | T | INTRON | NA | 0.162 |
| rs2303638 | 178567054 | A | G | INTRON | NA | 0.237 |
| rs34369919 | 178567082 | C | T | INTRON | NA | 0.071 |
| rs35311590 | 178567925 | C | T | INTRON | NA | 0.232 |
| rs141322886 | 178568106 | G | A | INTRON | NA | 0.002 |
| rs150776777 | 178568108 | G | A | INTRON | NA | 0.003 |
| rs12522721 | 178568148 | C | T | INTRON | NA | 0.130 |
| rs78480065 | 178568691 | G | A | INTRON | NA | 0.070 |
| rs4701055 | 178568874 | C | T | INTRON | NA | 0.158 |
| rs144660684 | 178568876 | C | G | INTRON | NA | 0.011 |
| rs9687070 | 178569149 | G | A | INTRON | NA | 0.374 |
| rs12515865 | 178569180 | A | T | INTRON | NA | 0.168 |
| rs58887996 | 178569941 | T | A | INTRON | NA | 0.230 |
| rs112902313 | 178570180 | G | A | INTRON | NA | 0.164 |
| rs185117796 | 178570271 | T | C | INTRON | NA | 0.006 |
| rs140886752 | 178570296 | A | G | INTRON | NA | 0.008 |
| rs10041147 | 178570631 | G | A | INTRON | NA | 0.352 |
| rs59775353 | 178570664 | G | A | INTRON | NA | 0.186 |
| rs186297063 | 178570685 | G | A | INTRON | NA | 0.001 |
| rs10038655 | 178570695 | A | G | INTRON | NA | 0.151 |
| rs10036369 | 178570902 | C | T | INTRON | NA | 0.383 |
| rs10039254 | 178570914 | A | G | INTRON | NA | 0.353 |
| rs7711539 | 178570926 | T | C | INTRON | NA | 0.352 |
| rs12517889 | 178571271 | T | C | INTRON | NA | 0.131 |
| rs34346845 | 178571507 | C | T | INTRON | NA | 0.229 |
| rs116046923 | 178571573 | C | A | INTRON | NA | 0.025 |
| rs4701057 | 178571857 | G | A | INTRON | NA | 0.412 |
| rs149328836 | 178572154 | G | A | INTRON | NA |  |
| rs11746863 | 178572268 | G | T | INTRON | NA | 0.150 |
| rs115478004 | 178572546 | T | C | INTRON | NA | 0.028 |
| rs34132209 | 178572884 | G | T | INTRON | NA | 0.226 |
| rs11955365 | 178573024 | T | G | DOWNSTREAM | NA | 0.191 |
| rs11951329 | 178573266 | C | G | DOWNSTREAM | NA | 0.185 |
| rs7736323 | 178573564 | G | A | DOWNSTREAM | NA | 0.164 |
| rs115894832 | 178573611 | G | T | DOWNSTREAM | NA | 0.002 |
| rs76119352 | 178573617 | G | A | DOWNSTREAM | NA | 0.157 |
| rs7727517 | 178573954 | T | C | DOWNSTREAM | NA | 0.356 |
| rs180913363 | 178574299 | A | G | DOWNSTREAM | NA | 0.003 |
| rs151146348 | 178574546 | C | T | DOWNSTREAM | NA | 0.003 |
| rs3776815 | 178574732 | C | A | DOWNSTREAM | NA | 0.385 |
| rs3776816 | 178574868 | A | G | DOWNSTREAM | NA | 0.397 |
| rs114893452 | 178575113 | G | A | DOWNSTREAM | NA | 0.001 |
| . | 178575327 | T | C | DOWNSTREAM | NA |  |
| rs10065978 | 178575384 | G | A | DOWNSTREAM | NA | 0.402 |
| rs10066034 | 178575429 | G | T | DOWNSTREAM | NA | 0.169 |
| rs3797595 | 178575917 | C | T | DOWNSTREAM | NA | 0.371 |
| rs3797596 | 178575948 | G | T | DOWNSTREAM | NA | 0.141 |
| rs79302514 | 178576204 | G | A | DOWNSTREAM | NA | 0.016 |
| rs141860824 | 178576243 | C | T | DOWNSTREAM | NA | 0.003 |
| rs140194527 | 178576446 | T | C | DOWNSTREAM | NA | 0.006 |
| rs3822599 | 178576471 | T | C | DOWNSTREAM | NA | 0.392 |
| rs3756566 | 178576631 | G | A | DOWNSTREAM | NA | 0.410 |
| rs3756567 | 178576680 | C | T | DOWNSTREAM | NA | 0.190 |
| rs2411908 | 178577231 | T | G | DOWNSTREAM | NA | 0.146 |
| rs2411907 | 178577267 | G | C | DOWNSTREAM | NA | 0.389 |
| rs143871229 | 178577290 | A | G | DOWNSTREAM | NA | 0.009 |
| rs2411906 | 178577392 | C | T | DOWNSTREAM | NA | 0.408 |
| rs2411905 | 178577487 | G | A | DOWNSTREAM | NA | 0.391 |
| rs35814218 | 178577570 | C | T | DOWNSTREAM | NA | 0.224 |
| rs111240301 | 178577588 | C | T | DOWNSTREAM | NA | 0.004 |
| . | 178577592 | C | T | DOWNSTREAM | NA |  |
| rs111928685 | 178577773 | G | A | DOWNSTREAM | NA | 0.011 |
| rs139658049 | 178578135 | C | T | NON_SYNONYMOUS_CODING | 0 | 0.008 |
| rs115530457 | 178578982 | A | G | INTRON | NA | 0.002 |
| rs2303637 | 178579119 | C | G | INTRON | NA | 0.244 |
| rs115550684 | 178579134 | C | T | INTRON | NA | 0.007 |
| rs11746123 | 178579394 | T | C | INTRON | NA | 0.160 |
| rs3776817 | 178579748 | C | T | INTRON | NA | 0.309 |
| rs35557468 | 178579850 | T | C | INTRON | NA | 0.040 |
| rs55863715 | 178579876 | G | A | INTRON | NA |  |
| rs3776818 | 178579914 | T | C | INTRON | NA | 0.469 |
| rs10076252 | 178580091 | G | A | INTRON | NA | 0.263 |
| rs115740207 | 178580646 | G | A | INTRON | NA | 0.026 |
| rs2278222 | 178581797 | C | T | INTRON | NA | 0.231 |
| rs2278221 | 178581859 | G | A | SYNONYMOUS_CODING | NA | 0.228 |
| rs4700784 | 178581977 | G | A | INTRON | NA | 0.181 |
| rs72818606 | 178582363 | C | A | INTRON | NA | 0.005 |
| rs56327529 | 178583165 | G | A | INTRON | NA | 0.013 |
| rs10041737 | 178583442 | C | G | INTRON | NA | 0.190 |
| rs7729756 | 178584302 | C | T | INTRON | NA | 0.304 |
| rs10079886 | 178584595 | T | C | INTRON | NA | 0.198 |
| rs4701059 | 178584779 | A | G | INTRON | NA | 0.168 |
| rs4701060 | 178584874 | C | T | INTRON | NA | 0.196 |
| rs4701061 | 178584944 | G | A | INTRON | NA | 0.197 |
| rs4701064 | 178584989 | A | G | INTRON | NA | 0.197 |
| rs151085877 | 178585058 | C | T | INTRON | NA | 0.008 |
| rs34865831 | 178585187 | C | T | INTRON | NA | 0.230 |
| rs72818607 | 178585394 | A | T | INTRON | NA | 0.005 |
| rs41285551 | 178585589 | C | T | INTRON | NA | 0.216 |
| rs147861022 | 178585692 | C | T | INTRON | NA | 0.001 |
| rs112433508 | 178586041 | C | A | INTRON | NA | 0.053 |
| rs114355563 | 178586270 | C | T | INTRON | NA | 0.008 |
| rs113936403 | 178586315 | G | A | INTRON | NA | 0.014 |
| rs75339809 | 178586349 | G | A | INTRON | NA | 0.001 |
| rs143564607 | 178586454 | G | A | INTRON | NA | 0.001 |
| . | 178586916 | T | C | INTRON | NA |  |
| . | 178587186 | G | A | INTRON | NA |  |
| . | 178587212 | C | G | INTRON | NA |  |
| rs12187962 | 178587296 | C | T | INTRON | NA | 0.267 |
| . | 178588227 | A | G | INTRON | NA |  |
| rs35718962 | 178588531 | G | C | INTRON | NA | 0.321 |
| . | 178589097 | C | G | INTRON | NA |  |
| rs145004720 | 178589608 | G | A | INTRON | NA | 0.010 |
| rs111611225 | 178589694 | C | T | INTRON | NA | 0.003 |
| rs4701065 | 178589930 | A | G | INTRON | NA | 0.439 |
| rs66541557 | 178590656 | T | C | INTRON | NA | 0.111 |
| . | 178590813 | C | T | INTRON | NA |  |
| rs182074541 | 178591161 | C | T | INTRON | NA | 0.014 |
| rs72818610 | 178591246 | G | A | INTRON | NA | 0.067 |
| rs138941969 | 178591294 | G | A | INTRON | NA | 0.007 |
| . | 178591424 | C | G | INTRON | NA |  |
| rs6899233 | 178591438 | G | A | INTRON | NA | 0.286 |
| rs142692720 | 178592037 | T | C | INTRON | NA | 0.005 |
| rs7706427 | 178592302 | C | T | INTRON | NA | 0.269 |
| . | 178593000 | C | T | INTRON | NA |  |
| rs79953818 | 178593631 | G | C | INTRON | NA | 0.086 |
| rs11960126 | 178593669 | C | T | INTRON | NA | 0.257 |
| rs34095418 | 178593899 | G | C | INTRON | NA |  |
| rs72818611 | 178594180 | A | G | INTRON | NA | 0.131 |
| rs10035188 | 178594647 | C | T | INTRON | NA | 0.197 |
| rs62396136 | 178594682 | G | T | INTRON | NA | 0.006 |
| rs116236689 | 178594976 | C | T | INTRON | NA | 0.018 |
| rs10071352 | 178595338 | C | G | INTRON | NA | 0.355 |
| . | 178595424 | G | A | INTRON | NA |  |
| rs4701067 | 178595483 | C | T | INTRON | NA | 0.325 |
| rs7732251 | 178595485 | C | T | INTRON | NA | 0.368 |
| rs4700785 | 178595504 | G | A | INTRON | NA | 0.306 |
| rs7735461 | 178595870 | G | A | INTRON | NA | 0.465 |
| rs116636097 | 178595877 | G | T | INTRON | NA | 0.013 |
| rs7718264 | 178595918 | A | G | INTRON | NA | 0.335 |
| . | 178596315 | C | T | INTRON | NA |  |
| rs147365846 | 178596370 | G | C | INTRON | NA | 0.010 |
| rs7704239 | 178596915 | C | G | INTRON | NA | 0.134 |
| . | 178597274 | C | T | INTRON | NA |  |
| rs60562987 | 178597400 | G | A | INTRON | NA | 0.206 |
| rs78988206 | 178598014 | A | G | INTRON | NA | 0.059 |
| rs13184510 | 178598691 | C | T | INTRON | NA | 0.017 |
| rs4700786 | 178598778 | A | G | INTRON | NA | 0.105 |
| rs10079445 | 178598862 | C | T | INTRON | NA | 0.055 |
| rs34540364 | 178599138 | C | T | INTRON | NA | 0.071 |
| rs35884442 | 178599170 | C | T | INTRON | NA | 0.098 |
| rs185389774 | 178599453 | C | G | INTRON | NA | 0.002 |
| rs1530499 | 178599617 | G | A | INTRON | NA | 0.341 |
| . | 178599813 | C | T | INTRON | NA |  |
| . | 178599869 | A | T | INTRON | NA |  |
| rs192089857 | 178600083 | C | T | INTRON | NA | 0.000 |
| . | 178601052 | T | G | INTRON | NA |  |
| rs60074362 | 178601085 | A | G | INTRON | NA | 0.129 |
| rs62396137 | 178601466 | G | A | INTRON | NA | 0.118 |
| rs888764 | 178601536 | C | T | INTRON | NA | 0.237 |
| rs115972004 | 178601767 | G | A | INTRON | NA | 0.086 |
| rs4701072 | 178601824 | G | A | INTRON | NA | 0.463 |
| rs184262303 | 178602027 | C | T | INTRON | NA | 0.016 |
| rs71589489 | 178602028 | G | A | INTRON | NA |  |
| . | 178602043 | A | G | INTRON | NA |  |
| rs873987 | 178602683 | G | A | INTRON | NA | 0.116 |
| rs1106062 | 178602734 | G | A | INTRON | NA | 0.457 |
| rs4374749 | 178602782 | A | G | INTRON | NA | 0.061 |
| . | 178603123 | A | C | INTRON | NA |  |
| rs73809706 | 178603525 | A | T | INTRON | NA | 0.095 |
| rs75593596 | 178603606 | G | A | INTRON | NA | 0.064 |
| rs4701077 | 178604377 | C | T | INTRON | NA | 0.478 |
| rs138151836 | 178604394 | C | A | INTRON | NA | 0.016 |
| rs142637445 | 178604414 | T | C | INTRON | NA | 0.011 |
| rs6862563 | 178604465 | G | A | INTRON | NA | 0.063 |
| . | 178605688 | C | T | INTRON | NA |  |
| rs10050501 | 178606073 | A | G | INTRON | NA | 0.041 |
| . | 178606106 | C | T | INTRON | NA |  |
| rs17668185 | 178606285 | A | G | INTRON | NA | 0.109 |
| rs182963078 | 178606421 | A | C | INTRON | NA | 0.003 |
| rs147662380 | 178607083 | A | G | INTRON | NA | 0.001 |
| rs888763 | 178607770 | T | C | INTRON | NA | 0.313 |
| rs1862212 | 178607922 | A | T | INTRON | NA | 0.414 |
| rs35462609 | 178608112 | G | A | SYNONYMOUS_CODING | NA | 0.011 |
| rs78222573 | 178608204 | G | A | INTRON | NA | 0.007 |
| . | 178608316 | C | G | INTRON | NA |  |
| rs11749583 | 178608341 | C | T | INTRON | NA | 0.057 |
| rs1110514 | 178608404 | T | A | INTRON | NA | 0.231 |
| rs71611480 | 178608463 | G | A | INTRON | NA | 0.016 |
| . | 178608523 | C | T | INTRON | NA |  |
| . | 178608821 | A | G | INTRON | NA |  |
| rs3797600 | 178609060 | T | C | INTRON | NA | 0.373 |
| rs3797601 | 178609213 | T | C | INTRON | NA | 0.231 |
| . | 178609352 | G | A | INTRON | NA |  |
| rs3797602 | 178609359 | G | T | INTRON | NA | 0.496 |
| rs35729255 | 178609614 | G | A | INTRON | NA | 0.013 |
| . | 178609756 | G | A | INTRON | NA |  |
| rs3776819 | 178610028 | T | C | INTRON | NA | 0.243 |
| rs10040944 | 178610730 | G | A | INTRON | NA | 0.128 |
| rs188787272 | 178611189 | C | T | INTRON | NA | 0.003 |
| rs252076 | 178611252 | G | A | INTRON | NA | 0.346 |
| rs112647030 | 178611331 | G | A | INTRON | NA | 0.053 |
| rs116434736 | 178611569 | C | G | INTRON | NA | 0.001 |
| rs420251 | 178612350 | A | G | INTRON | NA | 0.477 |
| . | 178612370 | G | C | INTRON | NA |  |
| rs252075 | 178612881 | G | C | INTRON | NA | 0.230 |
| rs252074 | 178613153 | C | T | INTRON | NA | 0.253 |
| . | 178613157 | G | A | INTRON | NA |  |
| . | 178613977 | C | T | INTRON | NA |  |
| rs252068 | 178614232 | C | G | INTRON | NA | 0.263 |
| rs252069 | 178614443 | T | C | INTRON | NA | 0.222 |
| rs194040 | 178614489 | G | A | INTRON | NA | 0.235 |
| rs252070 | 178614693 | G | A | INTRON | NA | 0.282 |
| rs3797606 | 178614799 | C | A | INTRON | NA | 0.037 |
| rs171667 | 178614867 | T | C | INTRON | NA | 0.222 |
| rs114899756 | 178615091 | C | T | INTRON | NA | 0.060 |
| rs10479527 | 178615092 | T | C | INTRON | NA | 0.004 |
| . | 178615122 | C | A | INTRON | NA |  |
| rs79176083 | 178615134 | C | A | INTRON | NA | 0.067 |
| rs187539 | 178615162 | G | A | INTRON | NA | 0.449 |
| rs34732189 | 178615578 | C | T | INTRON | NA | 0.027 |
| . | 178615732 | A | G | INTRON | NA |  |
| rs34556851 | 178615790 | T | C | INTRON | NA | 0.014 |
| rs34780896 | 178615875 | T | G | INTRON | NA | 0.015 |
| rs140105477 | 178615938 | T | G | INTRON | NA | 0.003 |
| . | 178615944 | C | T | INTRON | NA |  |
| rs252071 | 178616135 | T | C | INTRON | NA | 0.187 |
| rs62396156 | 178616188 | C | T | INTRON | NA | 0.001 |
| rs62396157 | 178616401 | T | C | INTRON | NA | 0.001 |
| rs454939 | 178616512 | T | C | INTRON | NA |  |
| rs59845470 | 178616565 | A | G | INTRON | NA | 0.043 |
| rs35601097 | 178616591 | T | C | INTRON | NA | 0.107 |
| . | 178616604 | C | T | INTRON | NA |  |
| rs34757332 | 178616675 | G | C | INTRON | NA | 0.029 |
| rs143677847 | 178616907 | G | A | INTRON | NA | 0.003 |
| rs12653014 | 178617269 | C | T | INTRON | NA | 0.050 |
| rs34688217 | 178617412 | G | T | INTRON | NA | 0.148 |
| rs181917965 | 178617450 | G | A | INTRON | NA | 0.001 |
| rs11249615 | 178617669 | G | T | INTRON | NA | 0.242 |
| rs11249616 | 178617695 | G | A | INTRON | NA | 0.229 |
| . | 178617863 | G | A | INTRON | NA |  |
| rs143060322 | 178618128 | G | A | INTRON | NA | 0.003 |
| rs7732747 | 178618159 | G | A | INTRON | NA | 0.055 |
| rs34478977 | 178618168 | C | T | INTRON | NA | 0.115 |
| rs7733929 | 178618188 | C | T | INTRON | NA | 0.233 |
| rs252073 | 178618219 | A | G | INTRON | NA | 0.000 |
| rs7732905 | 178618252 | G | A | INTRON | NA | 0.210 |
| . | 178618425 | C | T | INTRON | NA |  |
| rs189806773 | 178618447 | G | T | INTRON | NA |  |
| rs6895999 | 178618707 | C | A | INTRON | NA | 0.041 |
| rs6894805 | 178618727 | G | A | INTRON | NA | 0.043 |
| rs6859791 | 178618917 | C | T | INTRON | NA | 0.207 |
| rs6860197 | 178619190 | C | T | INTRON | NA | 0.086 |
| rs191802094 | 178619231 | G | A | INTRON | NA | 0.000 |
| . | 178619280 | C | T | INTRON | NA |  |
| rs11748179 | 178619384 | G | A | INTRON | NA | 0.059 |
| rs11741823 | 178619399 | C | A | INTRON | NA | 0.058 |
| rs10074081 | 178619774 | G | A | INTRON | NA | 0.155 |
| rs379589 | 178619896 | A | T | INTRON | NA | 0.192 |
| rs192067069 | 178619979 | C | T | INTRON | NA | 0.006 |
| rs4700787 | 178620251 | C | T | INTRON | NA | 0.200 |
| rs2052469 | 178620316 | C | T | INTRON | NA | 0.360 |
| rs4700788 | 178620555 | T | C | INTRON | NA | 0.380 |
| rs140502126 | 178620610 | T | C | INTRON | NA | 0.004 |
| . | 178620638 | G | A | INTRON | NA |  |
| rs10076130 | 178620710 | G | A | INTRON | NA | 0.338 |
| rs192723835 | 178620940 | G | A | INTRON | NA | 0.000 |
| rs115050997 | 178620974 | C | A | INTRON | NA | 0.004 |
| rs112650919 | 178621167 | C | T | INTRON | NA | 0.002 |
| rs3797609 | 178621505 | C | T | INTRON | NA | 0.376 |
| rs148549705 | 178621561 | C | G | INTRON | NA | 0.004 |
| rs3822601 | 178621576 | C | T | INTRON | NA | 0.381 |
| . | 178621680 | C | T | INTRON | NA |  |
| rs115948262 | 178621856 | T | C | INTRON | NA | 0.003 |
| rs193145294 | 178621904 | T | C | INTRON | NA | 0.002 |
| rs142116308 | 178622026 | C | G | INTRON | NA | 0.006 |
| rs153131 | 178622155 | T | C | INTRON | NA | 0.278 |
| rs111749915 | 178622353 | C | T | INTRON | NA | 0.002 |
| rs62396159 | 178622615 | C | T | INTRON | NA |  |
| rs184002569 | 178622721 | G | T | INTRON | NA | 0.001 |
| rs17668593 | 178622958 | C | T | INTRON | NA | 0.106 |
| rs6882867 | 178623021 | C | T | INTRON | NA | 0.023 |
| rs115551924 | 178623090 | G | T | INTRON | NA | 0.004 |
| rs4701079 | 178623326 | G | A | INTRON | NA | 0.210 |
| rs116754951 | 178623500 | G | A | INTRON | NA |  |
| rs751546 | 178623518 | G | C | INTRON | NA | 0.284 |
| rs2279979 | 178624101 | C | T | INTRON | NA | 0.299 |
| rs12189410 | 178624158 | T | C | INTRON | NA | 0.047 |
| rs28409173 | 178624844 | C | T | INTRON | NA | 0.089 |
| rs17079197 | 178624961 | C | T | INTRON | NA | 0.007 |
| rs7705793 | 178625525 | C | T | INTRON | NA | 0.029 |
| rs34241460 | 178626083 | C | T | INTRON | NA | 0.003 |
| rs77792767 | 178626372 | C | T | INTRON | NA | 0.110 |
| . | 178626418 | C | T | INTRON | NA |  |
| rs252060 | 178626437 | A | G | INTRON | NA | 0.145 |
| rs11747650 | 178626639 | T | C | INTRON | NA | 0.129 |
| . | 178626657 | G | A | INTRON | NA |  |
| rs10042679 | 178626760 | A | G | INTRON | NA | 0.263 |
| . | 178626892 | T | C | INTRON | NA |  |
| rs79287550 | 178627084 | G | C | INTRON | NA | 0.052 |
| . | 178627345 | G | A | INTRON | NA |  |
| rs188159177 | 178627448 | T | C | INTRON | NA | 0.001 |
| rs182076144 | 178627464 | C | G | INTRON | NA | 0.001 |
| rs143651284 | 178627680 | T | C | INTRON | NA | 0.000 |
| rs3797610 | 178627708 | A | C | INTRON | NA | 0.160 |
| rs11749201 | 178628067 | T | A | INTRON | NA | 0.092 |
| rs72818624 | 178628264 | C | T | INTRON | NA | 0.009 |
| rs141996878 | 178628289 | C | T | INTRON | NA | 0.005 |
| . | 178628404 | G | A | INTRON | NA |  |
| rs184834327 | 178628444 | C | T | INTRON | NA | 0.001 |
| rs194039 | 178628495 | C | T | INTRON | NA | 0.499 |
| rs189683990 | 178628515 | G | A | INTRON | NA | 0.000 |
| rs180886378 | 178628525 | G | A | INTRON | NA | 0.005 |
| . | 178628632 | C | A | INTRON | NA |  |
| rs2431435 | 178628771 | C | G | INTRON | NA | 0.161 |
| rs72818626 | 178628939 | G | A | INTRON | NA | 0.066 |
| rs11746118 | 178629251 | C | A | INTRON | NA | 0.084 |
| . | 178629270 | G | A | INTRON | NA |  |
| rs168773 | 178629361 | T | A | INTRON | NA | 0.457 |
| . | 178629534 | C | T | INTRON | NA |  |
| rs11960224 | 178629590 | T | A | INTRON | NA | 0.071 |
| rs76372972 | 178629673 | G | A | INTRON | NA | 0.002 |
| rs59809475 | 178629898 | A | G | INTRON | NA | 0.186 |
| rs143545670 | 178630211 | C | T | INTRON | NA | 0.001 |
| rs188359053 | 178630247 | G | A | INTRON | NA | 0.002 |
| . | 178630444 | C | T | INTRON | NA |  |
| rs252061 | 178630445 | G | A | INTRON | NA | 0.204 |
| rs34210609 | 178630705 | A | G | INTRON | NA | 0.254 |
| rs139894468 | 178630871 | G | A | INTRON | NA | 0.002 |
| rs77606167 | 178630876 | C | T | INTRON | NA | 0.006 |
| rs252062 | 178631251 | T | A | INTRON | NA | 0.456 |
| rs2431255 | 178631372 | T | G | INTRON | NA | 0.183 |
| . | 178631431 | C | T | INTRON | NA |  |
| rs72818631 | 178631497 | C | T | INTRON | NA | 0.058 |
| rs72818633 | 178631681 | C | T | INTRON | NA | 0.119 |
| rs3797612 | 178631699 | T | C | INTRON | NA | 0.469 |
| rs72818634 | 178631736 | G | A | INTRON | NA | 0.139 |
| rs3797613 | 178631820 | C | T | INTRON | NA | 0.102 |
| rs189773799 | 178631993 | C | T | INTRON | NA | 0.000 |
| rs614114 | 178632126 | C | T | INTRON | NA |  |
| . | 178632128 | G | A | INTRON | NA |  |
| rs252063 | 178632467 | T | C | INTRON | NA | 0.223 |
| rs252064 | 178632482 | G | A | INTRON | NA | 0.335 |
| rs439252 | 178633035 | C | T | INTRON | NA | 0.186 |
| rs17079202 | 178633088 | G | A | INTRON | NA | 0.021 |
| rs182762182 | 178633149 | G | A | INTRON | NA | 0.001 |
| rs112841247 | 178633219 | C | T | INTRON | NA | 0.003 |
| rs187053524 | 178633481 | G | A | INTRON | NA | 0.002 |
| rs113967154 | 178633775 | C | A | INTRON | NA | 0.004 |
| rs184269453 | 178633961 | C | T | INTRON | NA | 0.001 |
| rs457957 | 178633966 | C | T | INTRON | NA | 0.132 |
| . | 178634054 | T | C | INTRON | NA |  |
| . | 178634060 | C | T | INTRON | NA |  |
| rs115957547 | 178634211 | C | T | INTRON | NA | 0.002 |
| . | 178634342 | C | A | INTRON | NA |  |
| . | 178634385 | C | T | INTRON | NA |  |
| rs189164528 | 178634396 | G | A | INTRON | NA | 0.001 |
| rs77695342 | 178634411 | C | T | INTRON | NA | 0.002 |
| rs66565583 | 178634547 | G | A | SYNONYMOUS_CODING | NA | 0.136 |
| rs423552 | 178634619 | C | T | SYNONYMOUS_CODING | NA | 0.075 |
| rs398829 | 178634672 | C | T | NON_SYNONYMOUS_CODING | 0.56 | 0.432 |
| . | 178634681 | C | T | NON_SYNONYMOUS_CODING | 0.59 |  |
| rs11750821 | 178634683 | C | T | NON_SYNONYMOUS_CODING | 0.15 | 0.072 |
| rs59567206 | 178634704 | T | C | NON_SYNONYMOUS_CODING | 0.63 | 0.018 |
| rs141834320 | 178634964 | T | C | INTRON | NA | 0.006 |
| rs112729232 | 178634978 | A | T | INTRON | NA | 0.086 |
| rs416646 | 178635005 | C | T | INTRON | NA | 0.425 |
| . | 178635125 | C | T | INTRON | NA |  |
| rs115923899 | 178635462 | G | A | INTRON | NA | 0.022 |
| rs76810562 | 178635493 | C | T | INTRON | NA | 0.020 |
| rs62397463 | 178635551 | G | A | INTRON | NA | 0.004 |
| rs57326548 | 178635583 | C | T | INTRON | NA | 0.126 |
| rs55669906 | 178635641 | C | G | INTRON | NA | 0.096 |
| rs57207362 | 178635702 | G | A | INTRON | NA | 0.093 |
| rs58123454 | 178635731 | G | A | INTRON | NA | 0.096 |
| rs139127004 | 178635805 | C | T | INTRON | NA | 0.006 |
| rs77262167 | 178636136 | G | A | INTRON | NA | 0.023 |
| rs337807 | 178636202 | G | A | INTRON | NA | 0.211 |
| rs56061695 | 178636594 | G | A | INTRON | NA |  |
| rs56274637 | 178636700 | A | G | INTRON | NA | 0.134 |
| . | 178636808 | T | A | INTRON | NA |  |
| rs2411812 | 178637059 | T | A | INTRON | NA | 0.156 |
| rs337806 | 178637124 | G | T | INTRON | NA | 0.171 |
| rs1396438 | 178637179 | C | T | INTRON | NA | 0.078 |
| rs76291112 | 178637301 | G | T | INTRON | NA | 0.021 |
| . | 178637339 | C | T | INTRON | NA |  |
| rs7447544 | 178637502 | T | G | INTRON | NA | 0.148 |
| rs189256 | 178637965 | T | C | INTRON | NA | 0.223 |
| rs66629804 | 178638028 | T | G | INTRON | NA | 0.144 |
| rs115884109 | 178638451 | C | A | INTRON | NA | 0.002 |
| rs173072 | 178638560 | T | G | INTRON | NA | 0.240 |
| rs191415 | 178639040 | G | A | INTRON | NA | 0.236 |
| rs180045 | 178639086 | G | A | INTRON | NA | 0.236 |
| rs189255 | 178639127 | G | C | INTRON | NA | 0.237 |
| rs11750433 | 178639177 | C | T | INTRON | NA | 0.236 |
| rs11740803 | 178639213 | A | C | INTRON | NA | 0.236 |
| rs73329780 | 178639242 | G | A | INTRON | NA | 0.210 |
| rs111412180 | 178639388 | T | C | INTRON | NA | 0.241 |
| rs113963601 | 178639389 | G | A | INTRON | NA | 0.236 |
| rs113369755 | 178639453 | G | A | INTRON | NA | 0.206 |
| rs112992089 | 178639475 | C | A | INTRON | NA |  |
| rs186257452 | 178639485 | G | A | INTRON | NA |  |
| rs113008891 | 178639509 | T | C | INTRON | NA |  |
| rs13169936 | 178639546 | A | G | INTRON | NA | 0.235 |
| rs13188296 | 178639584 | C | T | INTRON | NA |  |
| rs58008039 | 178639738 | G | A | INTRON | NA |  |
| rs7734402 | 178639790 | A | G | INTRON | NA | 0.363 |
| rs7704451 | 178639879 | T | C | INTRON | NA | 0.336 |
| rs7718805 | 178639972 | C | A | INTRON | NA | 0.229 |
| rs7719124 | 178640129 | C | A | INTRON | NA | 0.267 |
| rs67436679 | 178640257 | C | T | INTRON | NA | 0.249 |
| rs114434633 | 178640300 | C | T | INTRON | NA | 0.004 |
| rs466750 | 178640760 | A | C | INTRON | NA | 0.233 |
| rs138101035 | 178640832 | C | T | INTRON | NA | 0.002 |
| rs59434527 | 178640833 | G | C | INTRON | NA | 0.106 |
| rs442406 | 178640982 | C | T | INTRON | NA | 0.236 |
| rs17606295 | 178641027 | C | T | INTRON | NA | 0.056 |
| rs662407 | 178641364 | A | G | INTRON | NA | 0.226 |
| . | 178641576 | G | A | INTRON | NA |  |
| rs459668 | 178641701 | G | A | INTRON | NA | 0.226 |
| rs79111715 | 178641756 | T | G | INTRON | NA | 0.017 |
| rs458272 | 178642008 | A | C | INTRON | NA | 0.226 |
| rs463455 | 178642030 | T | C | INTRON | NA | 0.198 |
| rs675880 | 178642105 | C | T | INTRON | NA | 0.226 |
| rs59887135 | 178642225 | G | A | INTRON | NA | 0.226 |
| rs60819052 | 178642310 | A | G | INTRON | NA | 0.226 |
| rs113610560 | 178642397 | T | C | INTRON | NA | 0.227 |
| rs112993967 | 178642405 | C | T | INTRON | NA | 0.227 |
| rs464156 | 178642524 | G | A | INTRON | NA | 0.360 |
| rs467320 | 178642590 | T | A | INTRON | NA | 0.224 |
| rs467333 | 178642627 | C | G | INTRON | NA | 0.226 |
| rs72818649 | 178642766 | A | G | INTRON | NA | 0.092 |
| rs150154249 | 178643432 | G | A | INTRON | NA | 0.100 |
| rs138925084 | 178643454 | C | T | INTRON | NA | 0.093 |
| rs115958611 | 178643513 | G | A | INTRON | NA | 0.018 |
| rs11747646 | 178643536 | G | T | INTRON | NA | 0.199 |
| rs11744583 | 178643569 | A | G | INTRON | NA | 0.140 |
| rs191363750 | 178643578 | T | C | INTRON | NA | 0.002 |
| . | 178643588 | G | C | INTRON | NA |  |
| . | 178643593 | C | T | INTRON | NA |  |
| rs2457099 | 178643740 | A | G | INTRON | NA | 0.493 |
| rs68081358 | 178643803 | A | G | INTRON | NA | 0.111 |
| rs463901 | 178644294 | A | G | INTRON | NA | 0.398 |
| rs465621 | 178644592 | G | T | INTRON | NA | 0.242 |
| rs74942845 | 178644672 | G | T | INTRON | NA | 0.076 |
| rs78642532 | 178644943 | A | G | INTRON | NA | 0.022 |
| rs467419 | 178645055 | T | C | INTRON | NA | 0.363 |
| rs456135 | 178645076 | C | G | INTRON | NA | 0.132 |
| rs464536 | 178645203 | A | G | INTRON | NA | 0.376 |
| rs113871315 | 178645204 | G | A | INTRON | NA | 0.001 |
| rs461898 | 178645310 | T | C | INTRON | NA | 0.430 |
| rs73331552 | 178645325 | C | T | INTRON | NA | 0.101 |
| rs55885988 | 178645638 | G | A | INTRON | NA | 0.118 |
| rs114118063 | 178645702 | C | T | INTRON | NA | 0.007 |
| rs389558 | 178645742 | C | T | INTRON | NA | 0.358 |
| rs466752 | 178645802 | A | G | INTRON | NA | 0.455 |
| rs455655 | 178646058 | G | C | INTRON | NA | 0.358 |
| rs463435 | 178646193 | A | G | INTRON | NA | 0.358 |
| rs7704836 | 178646288 | A | G | INTRON | NA | 0.358 |
| . | 178646578 | G | A | INTRON | NA |  |
| rs75963502 | 178646906 | T | C | INTRON | NA |  |
| rs11739937 | 178647124 | C | T | INTRON | NA | 0.218 |
| rs189765827 | 178647145 | G | A | INTRON | NA | 0.002 |
| rs6873892 | 178647219 | T | G | INTRON | NA | 0.131 |
| rs1979979 | 178647275 | G | A | INTRON | NA | 0.025 |
| . | 178647338 | C | A | INTRON | NA |  |
| rs411804 | 178647388 | C | T | INTRON | NA | 0.110 |
| . | 178647838 | G | A | INTRON | NA |  |
| . | 178647896 | C | T | INTRON | NA |  |
| rs72818655 | 178648002 | C | T | INTRON | NA | 0.079 |
| rs1623885 | 178648041 | C | T | INTRON | NA | 0.135 |
| rs35523163 | 178648193 | T | C | INTRON | NA | 0.151 |
| rs141417777 | 178648270 | C | T | INTRON | NA | 0.006 |
| rs1643811 | 178648369 | G | A | INTRON | NA | 0.489 |
| rs72818658 | 178648450 | C | T | INTRON | NA | 0.079 |
| rs77801323 | 178648511 | C | G | INTRON | NA | 0.023 |
| . | 178648736 | A | T | INTRON | NA |  |
| . | 178648757 | C | T | INTRON | NA |  |
| rs79609403 | 178648984 | C | T | INTRON | NA | 0.026 |
| rs13173873 | 178649283 | G | A | INTRON | NA | 0.123 |
| rs59676020 | 178649513 | G | A | INTRON | NA | 0.134 |
| rs6881760 | 178649608 | A | G | INTRON | NA | 0.035 |
| rs6859608 | 178649610 | G | T | INTRON | NA | 0.035 |
| rs72818660 | 178650167 | C | T | INTRON | NA | 0.007 |
| rs184016185 | 178650300 | C | T | INTRON | NA | 0.023 |
| rs166145 | 178650596 | C | T | INTRON | NA | 0.381 |
| . | 178650671 | A | T | INTRON | NA |  |
| . | 178651071 | G | A | INTRON | NA |  |
| rs17079224 | 178651122 | A | T | INTRON | NA | 0.133 |
| rs252067 | 178651285 | C | T | INTRON | NA | 0.115 |
| rs459319 | 178651472 | T | C | INTRON | NA | 0.087 |
| rs13360479 | 178651533 | T | C | INTRON | NA | 0.081 |
| rs467289 | 178651577 | G | A | INTRON | NA |  |
| rs7715542 | 178651808 | G | C | INTRON | NA | 0.257 |
| rs7716676 | 178651832 | C | T | INTRON | NA | 0.211 |
| rs111951512 | 178651968 | C | T | INTRON | NA | 0.010 |
| rs462644 | 178652196 | G | A | INTRON | NA | 0.239 |
| rs7716184 | 178652227 | G | A | INTRON | NA | 0.158 |
| rs458752 | 178652282 | G | A | INTRON | NA | 0.134 |
| rs143128088 | 178652420 | G | A | INTRON | NA | 0.001 |
| rs6881376 | 178652942 | G | C | INTRON | NA | 0.304 |
| rs190547989 | 178652987 | G | A | INTRON | NA | 0.002 |
| rs7704240 | 178653102 | A | C | INTRON | NA | 0.338 |
| rs35510292 | 178653337 | A | G | INTRON | NA | 0.294 |
| rs708320 | 178653454 | T | G | INTRON | NA | 0.342 |
| . | 178653545 | T | A | INTRON | NA |  |
| . | 178653547 | G | A | INTRON | NA |  |
| rs6883699 | 178653594 | C | T | INTRON | NA | 0.244 |
| rs457954 | 178653830 | C | G | INTRON | NA | 0.370 |
| rs2411810 | 178653849 | G | A | INTRON | NA | 0.271 |
| rs69638 | 178654216 | G | C | INTRON | NA | 0.348 |
| rs192337170 | 178654537 | T | C | INTRON | NA | 0.000 |
| rs455452 | 178654622 | T | C | INTRON | NA | 0.411 |
| rs116668730 | 178654839 | G | T | INTRON | NA | 0.009 |
| . | 178654857 | G | A | INTRON | NA |  |
| rs464850 | 178655117 | T | C | INTRON | NA | 0.233 |
| rs10044299 | 178655335 | G | C | INTRON | NA | 0.185 |
| rs7704320 | 178655407 | G | C | INTRON | NA | 0.425 |
| rs431472 | 178655538 | T | C | INTRON | NA | 0.343 |
| rs2431254 | 178656463 | T | C | INTRON | NA | 0.142 |
| . | 178656639 | C | A | INTRON | NA |  |
| rs138250263 | 178656640 | G | A | INTRON | NA | 0.001 |
| rs72818672 | 178656664 | A | T | INTRON | NA | 0.237 |
| rs4701081 | 178657017 | G | T | INTRON | NA | 0.207 |
| rs4701082 | 178657169 | G | A | INTRON | NA | 0.452 |
| rs4701083 | 178657190 | G | A | INTRON | NA | 0.023 |
| . | 178657536 | G | C | INTRON | NA |  |
| . | 178657590 | C | T | INTRON | NA |  |
| rs2003218 | 178657763 | G | A | INTRON | NA | 0.353 |
| rs139352577 | 178657869 | G | A | INTRON | NA | 0.025 |
| rs2261740 | 178657991 | A | G | INTRON | NA | 0.444 |
| rs1109179 | 178658114 | A | G | INTRON | NA | 0.177 |
| rs11249617 | 178658161 | G | T | INTRON | NA |  |
| rs1109178 | 178658440 | G | T | INTRON | NA | 0.206 |
| . | 178658455 | G | A | INTRON | NA |  |
| . | 178658788 | C | T | INTRON | NA |  |
| rs145691514 | 178658845 | G | A | INTRON | NA | 0.001 |
| rs116720269 | 178659274 | G | A | INTRON | NA | 0.003 |
| rs11739530 | 178659639 | G | A | INTRON | NA | 0.152 |
| rs456909 | 178659691 | C | T | INTRON | NA | 0.383 |
| . | 178660548 | C | T | INTRON | NA |  |
| rs76312903 | 178660770 | C | T | INTRON | NA | 0.036 |
| rs114330092 | 178661923 | G | T | INTRON | NA | 0.024 |
| rs115327276 | 178662011 | G | C | INTRON | NA | 0.004 |
| rs113960013 | 178662302 | T | G | INTRON | NA | 0.035 |
| rs468039 | 178662427 | A | G | INTRON | NA | 0.020 |
| rs467017 | 178663005 | G | T | INTRON | NA | 0.431 |
| rs469290 | 178663225 | T | C | INTRON | NA | 0.435 |
| rs469090 | 178663338 | G | A | INTRON | NA | 0.138 |
| rs469568 | 178663408 | A | C | INTRON | NA | 0.396 |
| rs78152777 | 178663463 | C | A | INTRON | NA | 0.040 |
| rs151249122 | 178663653 | G | A | INTRON | NA | 0.003 |
| rs11741099 | 178664463 | A | G | INTRON | NA | 0.290 |
| rs112504392 | 178664577 | G | A | INTRON | NA | 0.010 |
| rs75029825 | 178664820 | C | T | INTRON | NA | 0.024 |
| rs11746802 | 178665185 | C | T | INTRON | NA | 0.278 |
| rs469099 | 178665240 | G | A | INTRON | NA | 0.081 |
| rs13165525 | 178665562 | G | A | INTRON | NA | 0.152 |
| rs469262 | 178665824 | A | G | INTRON | NA | 0.455 |
| rs115830792 | 178665954 | G | A | INTRON | NA | 0.025 |
| rs469229 | 178666066 | C | T | INTRON | NA | 0.081 |
| rs182624583 | 178666735 | G | A | INTRON | NA | 0.002 |
| rs11741111 | 178666752 | G | C | INTRON | NA | 0.240 |
| rs469396 | 178667395 | G | C | INTRON | NA | 0.373 |
| . | 178667472 | G | A | INTRON | NA |  |
| rs468723 | 178667778 | A | G | INTRON | NA | 0.382 |
| rs72818683 | 178667899 | G | C | INTRON | NA | 0.013 |
| rs467604 | 178667984 | C | T | INTRON | NA | 0.466 |
| rs6868427 | 178668535 | C | T | INTRON | NA | 0.385 |
| rs12153128 | 178668714 | G | A | INTRON | NA | 0.260 |
| rs77081337 | 178668859 | G | A | INTRON | NA | 0.016 |
| rs338874 | 178668964 | G | C | INTRON | NA | 0.380 |
| rs116342625 | 178669153 | T | G | INTRON | NA | 0.019 |
| rs338875 | 178669297 | C | T | INTRON | NA | 0.326 |
| rs4700789 | 178669361 | A | G | INTRON | NA | 0.244 |
| rs338876 | 178669870 | A | G | INTRON | NA | 0.479 |
| rs11738217 | 178669893 | C | T | INTRON | NA | 0.311 |
| rs11249618 | 178670507 | T | C | INTRON | NA | 0.318 |
| rs77796045 | 178670796 | A | G | INTRON | NA | 0.019 |
| rs78166651 | 178670975 | G | A | INTRON | NA | 0.021 |
| rs11743204 | 178671014 | T | C | INTRON | NA | 0.318 |
| rs4700790 | 178671164 | G | A | INTRON | NA | 0.343 |
| rs6601023 | 178671774 | A | G | INTRON | NA | 0.321 |
| rs74318323 | 178671866 | C | T | INTRON | NA | 0.021 |
| rs11746596 | 178672483 | G | A | INTRON | NA | 0.308 |
| rs11743568 | 178672539 | A | G | INTRON | NA | 0.310 |
| rs6889935 | 178672543 | G | A | INTRON | NA | 0.172 |
| rs77827950 | 178672940 | T | C | INTRON | NA | 0.021 |
| . | 178672972 | A | T | INTRON | NA |  |
| rs76681953 | 178673638 | T | A | INTRON | NA | 0.019 |
| rs10059514 | 178673915 | T | C | INTRON | NA | 0.230 |
| rs1644144 | 178673988 | T | C | INTRON | NA | 0.221 |
| rs4701085 | 178674076 | A | G | INTRON | NA | 0.169 |
| rs466912 | 178675021 | A | G | INTRON | NA | 0.093 |
| rs7716112 | 178675065 | G | A | INTRON | NA | 0.194 |
| . | 178675541 | G | C | INTRON | NA |  |
| rs116430077 | 178675645 | A | T | INTRON | NA | 0.006 |
| rs191300040 | 178676634 | C | T | INTRON | NA | 0.006 |
| rs448543 | 178677061 | T | C | INTRON | NA | 0.137 |
| rs11743904 | 178677149 | T | C | INTRON | NA | 0.008 |
| . | 178677899 | G | T | INTRON | NA |  |
| rs6894964 | 178678309 | C | T | INTRON | NA | 0.038 |
| rs2054782 | 178678417 | C | T | INTRON | NA | 0.201 |
| rs13155737 | 178678529 | T | A | INTRON | NA | 0.199 |
| . | 178678747 | G | A | INTRON | NA |  |
| . | 178678806 | C | T | INTRON | NA |  |
| rs62394987 | 178678919 | C | G | INTRON | NA | 0.191 |
| rs180287 | 178678978 | G | C | INTRON | NA | 0.031 |
| rs192088719 | 178679237 | C | G | INTRON | NA | 0.002 |
| . | 178679389 | T | A | INTRON | NA |  |
| rs338877 | 178679390 | T | A | INTRON | NA | 0.210 |
| rs79667494 | 178679462 | T | C | INTRON | NA | 0.022 |
| rs338878 | 178679557 | A | T | INTRON | NA | 0.101 |
| rs182734200 | 178681017 | A | G | INTRON | NA | 0.001 |
| . | 178681607 | G | C | INTRON | NA |  |
| rs378074 | 178682067 | A | G | INTRON | NA | 0.490 |
| rs6877180 | 178682209 | C | T | INTRON | NA | 0.201 |
| . | 178682325 | A | G | INTRON | NA |  |
| rs114751054 | 178682694 | G | A | INTRON | NA | 0.019 |
| rs139080330 | 178682826 | G | A | INTRON | NA | 0.002 |
| rs55965316 | 178683054 | C | T | INTRON | NA | 0.034 |
| . | 178683074 | G | A | INTRON | NA |  |
| rs144467956 | 178683094 | G | A | INTRON | NA | 0.006 |
| . | 178683817 | C | T | INTRON | NA |  |
| rs150198267 | 178684014 | A | G | INTRON | NA | 0.023 |
| rs461542 | 178684261 | T | C | INTRON | NA | 0.060 |
| . | 178684332 | G | A | INTRON | NA |  |
| rs75386558 | 178684375 | G | A | INTRON | NA | 0.005 |
| rs74490385 | 178684499 | G | C | INTRON | NA | 0.037 |
| rs191453 | 178684555 | G | A | INTRON | NA | 0.221 |
| rs148404938 | 178685002 | G | A | INTRON | NA | 0.001 |
| rs77826120 | 178685058 | G | A | INTRON | NA | 0.020 |
| rs340119 | 178685386 | T | C | INTRON | NA | 0.118 |
| rs13179057 | 178685479 | C | G | INTRON | NA | 0.242 |
| rs13178420 | 178685790 | G | A | INTRON | NA | 0.221 |
| rs13169291 | 178685950 | T | G | INTRON | NA | 0.192 |
| rs76604404 | 178686012 | C | A | INTRON | NA | 0.021 |
| rs114327132 | 178686053 | G | A | INTRON | NA | 0.020 |
| rs17607156 | 178686332 | G | A | INTRON | NA | 0.219 |
| rs340124 | 178686590 | G | A | INTRON | NA | 0.427 |
| rs340123 | 178686624 | A | G | INTRON | NA | 0.059 |
| rs173104 | 178686928 | C | T | INTRON | NA | 0.462 |
| . | 178687802 | G | A | INTRON | NA |  |
| rs459191 | 178687911 | C | T | INTRON | NA | 0.011 |
| rs462815 | 178687913 | T | C | INTRON | NA | 0.420 |
| rs186237686 | 178688073 | T | C | INTRON | NA | 0.002 |
| rs340121 | 178688093 | C | T | INTRON | NA | 0.418 |
| rs72818696 | 178688173 | G | T | INTRON | NA | 0.144 |
| rs115045337 | 178688488 | C | T | INTRON | NA | 0.027 |
| rs35932911 | 178688494 | C | T | INTRON | NA | 0.040 |
| . | 178688917 | C | T | INTRON | NA |  |
| rs12652571 | 178689042 | G | T | INTRON | NA | 0.027 |
| rs116666021 | 178689209 | C | T | INTRON | NA | 0.024 |
| rs35009598 | 178689278 | T | A | INTRON | NA | 0.027 |
| rs17079267 | 178689326 | G | A | INTRON | NA | 0.114 |
| rs338883 | 178689403 | A | G | INTRON | NA | 0.208 |
| rs9885424 | 178689856 | C | T | INTRON | NA | 0.114 |
| rs338882 | 178690725 | G | A | INTRON | NA | 0.456 |
| rs42875 | 178690776 | A | G | INTRON | NA | 0.127 |
| rs138112685 | 178690838 | G | A | INTRON | NA |  |
| rs77822365 | 178690869 | C | T | INTRON | NA | 0.024 |
| rs116633076 | 178691005 | C | A | INTRON | NA | 0.027 |
| rs12514197 | 178691301 | T | C | INTRON | NA | 0.203 |
| . | 178691550 | C | T | INTRON | NA |  |
| rs142643768 | 178691594 | C | T | INTRON | NA | 0.012 |
| rs17670161 | 178691595 | G | A | INTRON | NA | 0.205 |
| rs75105657 | 178691677 | C | T | INTRON | NA | 0.029 |
| . | 178691749 | G | A | INTRON | NA |  |
| rs149221202 | 178692272 | C | T | INTRON | NA | 0.003 |
| rs34799208 | 178692379 | C | T | INTRON | NA | 0.388 |
| rs116438529 | 178692439 | C | T | INTRON | NA | 0.009 |
| rs40141 | 178692491 | G | C | INTRON | NA | 0.126 |
| rs17079283 | 178692763 | G | A | INTRON | NA | 0.323 |
| rs35754 | 178692930 | A | G | INTRON | NA | 0.366 |
| rs6601026 | 178692932 | A | G | INTRON | NA | 0.400 |
| rs6601027 | 178693083 | C | T | INTRON | NA | 0.386 |
| rs72818699 | 178693464 | C | T | INTRON | NA | 0.100 |
| rs147352729 | 178693485 | G | A | INTRON | NA | 0.008 |
| rs17079284 | 178693963 | T | C | INTRON | NA | 0.097 |
| rs114048458 | 178694129 | C | G | INTRON | NA | 0.018 |
| rs7701383 | 178694400 | T | C | INTRON | NA | 0.403 |
| rs13189853 | 178694707 | C | A | INTRON | NA | 0.202 |
| . | 178695001 | G | A | INTRON | NA |  |
| rs6601028 | 178695086 | A | G | INTRON | NA | 0.403 |
| rs78825768 | 178695106 | T | C | INTRON | NA | 0.012 |
| rs76702112 | 178695446 | A | G | INTRON | NA | 0.018 |
| rs75542619 | 178695537 | G | A | INTRON | NA | 0.018 |
| . | 178695722 | G | A | INTRON | NA |  |
| rs73333240 | 178696225 | C | G | INTRON | NA | 0.067 |
| rs4700794 | 178696348 | T | G | INTRON | NA | 0.291 |
| rs163489 | 178696394 | A | G | INTRON | NA | 0.381 |
| . | 178696475 | G | A | INTRON | NA |  |
| rs145883167 | 178696558 | C | T | INTRON | NA | 0.034 |
| rs147093552 | 178697001 | G | A | INTRON | NA | 0.010 |
| rs13158356 | 178697223 | A | G | INTRON | NA | 0.259 |
| rs66961507 | 178697281 | G | A | INTRON | NA | 0.139 |
| rs163490 | 178697317 | T | C | INTRON | NA | 0.015 |
| rs60007930 | 178697544 | G | A | INTRON | NA | 0.096 |
| rs78736869 | 178697791 | C | T | INTRON | NA | 0.011 |
| rs11950390 | 178697827 | T | C | INTRON | NA | 0.356 |
| rs4701088 | 178698324 | G | A | INTRON | NA |  |
| rs62394996 | 178698344 | A | G | INTRON | NA | 0.261 |
| rs141802850 | 178698409 | C | A | INTRON | NA | 0.004 |
| rs62394997 | 178698421 | G | A | INTRON | NA | 0.100 |
| rs62394998 | 178698545 | A | G | INTRON | NA | 0.384 |
| rs901253 | 178698709 | C | T | INTRON | NA | 0.388 |
| rs115264712 | 178698881 | C | T | INTRON | NA | 0.015 |
| rs77157918 | 178698941 | C | T | INTRON | NA | 0.022 |
| rs115532608 | 178699146 | C | T | INTRON | NA | 0.013 |
| rs901254 | 178699311 | C | T | INTRON | NA | 0.012 |
| rs75669660 | 178699583 | C | T | INTRON | NA | 0.017 |
| rs79956663 | 178700358 | G | A | INTRON | NA | 0.017 |
| . | 178700633 | C | A | INTRON | NA |  |
| rs57392122 | 178700660 | T | C | INTRON | NA | 0.125 |
| rs58570299 | 178700900 | C | T | INTRON | NA | 0.096 |
| . | 178700996 | C | T | INTRON | NA |  |
| rs6884564 | 178701079 | C | G | INTRON | NA | 0.326 |
| rs76466021 | 178701530 | G | A | INTRON | NA | 0.019 |
| rs2013411 | 178701724 | C | T | INTRON | NA | 0.249 |
| rs78191428 | 178701825 | G | T | INTRON | NA | 0.018 |
| rs55715334 | 178702204 | A | G | INTRON | NA | 0.126 |
| rs248184 | 178702280 | A | C | INTRON | NA | 0.156 |
| rs12153439 | 178702348 | G | T | INTRON | NA | 0.358 |
| rs75091832 | 178702553 | G | A | INTRON | NA | 0.023 |
| rs56832700 | 178702598 | G | A | INTRON | NA | 0.030 |
| . | 178702703 | G | A | INTRON | NA |  |
| rs181166473 | 178702704 | T | C | INTRON | NA | 0.005 |
| rs78767149 | 178702794 | C | T | INTRON | NA | 0.018 |
| rs77485023 | 178702939 | G | A | INTRON | NA | 0.023 |
| . | 178702980 | G | A | INTRON | NA |  |
| . | 178702981 | C | A | INTRON | NA |  |
| rs74472167 | 178703084 | C | G | INTRON | NA | 0.015 |
| rs75467736 | 178703109 | A | G | INTRON | NA | 0.018 |
| . | 178703158 | C | T | INTRON | NA |  |
| rs78074186 | 178703219 | G | A | INTRON | NA | 0.018 |
| . | 178703418 | G | A | INTRON | NA |  |
| rs11249621 | 178703432 | C | G | INTRON | NA | 0.237 |
| rs28692855 | 178703520 | A | C | INTRON | NA | 0.015 |
| rs4701089 | 178704106 | T | C | INTRON | NA | 0.449 |
| rs62395001 | 178704305 | C | T | INTRON | NA | 0.221 |
| rs71596415 | 178705110 | A | C | INTRON | NA |  |
| rs71596416 | 178705122 | A | G | INTRON | NA |  |
| . | 178705171 | C | G | INTRON | NA |  |
| rs377841 | 178705430 | C | T | INTRON | NA | 0.074 |
| rs116658311 | 178705623 | C | T | INTRON | NA | 0.003 |
| rs461539 | 178705648 | A | G | INTRON | NA | 0.009 |
| rs13165403 | 178705707 | C | T | INTRON | NA | 0.239 |
| rs74451768 | 178705997 | G | C | INTRON | NA | 0.024 |
| rs62395003 | 178706006 | T | C | INTRON | NA | 0.135 |
| rs28484587 | 178706121 | T | C | INTRON | NA | 0.284 |
| rs6895117 | 178706162 | T | C | INTRON | NA | 0.250 |
| rs76885140 | 178706576 | G | A | INTRON | NA | 0.020 |
| rs459770 | 178707077 | A | G | INTRON | NA | 0.382 |
| rs10053515 | 178707115 | T | G | INTRON | NA | 0.289 |
| . | 178707301 | G | A | INTRON | NA |  |
| rs74713493 | 178707459 | C | T | INTRON | NA | 0.087 |
| rs366480 | 178707639 | G | C | INTRON | NA | 0.270 |
| rs648863 | 178707711 | G | A | INTRON | NA | 0.148 |
| rs193135527 | 178707736 | G | A | INTRON | NA | 0.001 |
| rs74293437 | 178707831 | A | G | INTRON | NA | 0.113 |
| rs55742814 | 178707866 | C | T | INTRON | NA | 0.258 |
| rs377379 | 178707920 | G | A | INTRON | NA | 0.041 |
| rs184027964 | 178708060 | T | C | INTRON | NA | 0.003 |
| rs407818 | 178708209 | A | T | INTRON | NA | 0.364 |
| rs410826 | 178708474 | C | T | INTRON | NA | 0.087 |
| rs11249623 | 178708591 | G | A | INTRON | NA | 0.448 |
| rs78190819 | 178708640 | G | A | INTRON | NA | 0.016 |
| . | 178708668 | T | G | INTRON | NA |  |
| rs189356812 | 178708845 | C | G | INTRON | NA | 0.001 |
| rs4473760 | 178708951 | G | A | INTRON | NA | 0.016 |
| rs13172933 | 178710228 | A | G | INTRON | NA | 0.104 |
| rs13153552 | 178710254 | C | T | INTRON | NA | 0.101 |
| rs116183056 | 178711202 | C | T | INTRON | NA | 0.030 |
| rs113311236 | 178711283 | T | C | INTRON | NA | 0.230 |
| rs13158232 | 178711593 | G | A | INTRON | NA | 0.103 |
| rs6888613 | 178712059 | T | C | INTRON | NA | 0.373 |
| rs413912 | 178712347 | C | G | INTRON | NA | 0.183 |
| rs114732867 | 178712915 | T | C | INTRON | NA | 0.006 |
| rs338880 | 178713260 | C | T | INTRON | NA | 0.388 |
| rs234994 | 178713383 | A | G | INTRON | NA | 0.022 |
| . | 178713661 | C | G | INTRON | NA |  |
| rs338881 | 178713712 | G | A | INTRON | NA | 0.155 |
| rs1078474 | 178714068 | A | G | INTRON | NA | 0.016 |
| rs116267285 | 178714292 | C | T | INTRON | NA | 0.009 |
| rs98071 | 178714358 | A | G | INTRON | NA | 0.210 |
| rs98070 | 178714378 | G | A | INTRON | NA | 0.157 |
| rs111995879 | 178715230 | C | T | INTRON | NA | 0.010 |
| . | 178715673 | C | T | INTRON | NA |  |
| rs72820615 | 178716207 | C | T | INTRON | NA | 0.244 |
| rs181797 | 178716389 | A | G | INTRON | NA | 0.244 |
| rs6881016 | 178716569 | G | A | INTRON | NA | 0.259 |
| rs163493 | 178717011 | C | G | INTRON | NA |  |
| rs35419568 | 178717156 | T | C | INTRON | NA | 0.138 |
| . | 178717290 | C | G | INTRON | NA |  |
| rs163323 | 178717559 | G | A | INTRON | NA | 0.439 |
| rs188450929 | 178717576 | G | A | INTRON | NA | 0.002 |
| rs12152929 | 178718346 | C | T | INTRON | NA | 0.029 |
| rs116681650 | 178718371 | C | T | INTRON | NA | 0.009 |
| rs111936171 | 178718974 | G | A | INTRON | NA | 0.001 |
| rs71611484 | 178719185 | G | C | INTRON | NA | 0.100 |
| rs4700798 | 178719364 | T | C | INTRON | NA | 0.244 |
| rs163491 | 178719448 | T | A | INTRON | NA | 0.227 |
| . | 178719533 | T | C | INTRON | NA |  |
| rs1862266 | 178719705 | T | C | INTRON | NA | 0.183 |
| rs163324 | 178720043 | G | A | INTRON | NA | 0.076 |
| rs78509577 | 178720647 | C | T | INTRON | NA | 0.211 |
| . | 178720949 | T | G | INTRON | NA |  |
| rs166031 | 178720968 | A | G | INTRON | NA | 0.257 |
| rs111849414 | 178721434 | C | T | INTRON | NA | 0.009 |
| . | 178721852 | C | T | INTRON | NA |  |
| . | 178721988 | C | T | INTRON | NA |  |
| rs340117 | 178722024 | G | A | INTRON | NA | 0.010 |
| rs6874467 | 178722356 | A | G | INTRON | NA | 0.268 |
| rs405302 | 178722432 | C | A | INTRON | NA | 0.007 |
| . | 178722623 | C | T | INTRON | NA |  |
| rs457659 | 178722700 | A | C | INTRON | NA | 0.209 |
| rs111945621 | 178723105 | G | A | INTRON | NA | 0.009 |
| rs340116 | 178723301 | C | T | INTRON | NA | 0.240 |
| rs144766741 | 178723381 | G | A | INTRON | NA | 0.001 |
| rs140574356 | 178723708 | C | T | INTRON | NA | 0.006 |
| rs141244609 | 178724571 | T | C | INTRON | NA | 0.004 |
| rs467053 | 178724727 | T | C | INTRON | NA | 0.476 |
| . | 178724738 | C | T | INTRON | NA |  |
| rs12652703 | 178724795 | G | C | INTRON | NA | 0.308 |
| rs67025042 | 178724807 | G | C | INTRON | NA | 0.256 |
| rs112641894 | 178724882 | G | A | INTRON | NA | 0.003 |
| rs139023691 | 178725062 | C | T | INTRON | NA | 0.004 |
| rs116541085 | 178725307 | T | C | INTRON | NA | 0.001 |
| rs467342 | 178725532 | C | T | INTRON | NA | 0.347 |
| rs7702938 | 178725603 | G | C | INTRON | NA | 0.254 |
| rs17607923 | 178725823 | T | C | INTRON | NA | 0.011 |
| rs144511335 | 178725977 | A | G | INTRON | NA | 0.000 |
| rs111890921 | 178726455 | A | G | INTRON | NA | 0.018 |
| rs4485912 | 178726502 | C | G | INTRON | NA | 0.241 |
| . | 178726509 | A | G | INTRON | NA |  |
| rs55698132 | 178726785 | T | A | INTRON | NA | 0.012 |
| rs190635453 | 178726922 | C | T | INTRON | NA | 0.048 |
| rs28380667 | 178727060 | C | T | INTRON | NA | 0.150 |
| rs2457098 | 178727074 | C | T | INTRON | NA | 0.416 |
| rs929771 | 178727670 | G | A | INTRON | NA | 0.026 |
| rs929770 | 178727697 | T | A | INTRON | NA | 0.027 |
| rs929769 | 178727997 | T | C | INTRON | NA | 0.499 |
| rs149980005 | 178728354 | A | C | INTRON | NA | 0.004 |
| rs149210855 | 178728546 | C | T | INTRON | NA | 0.002 |
| rs2457100 | 178728830 | C | T | INTRON | NA | 0.432 |
| rs412398 | 178728907 | C | T | INTRON | NA | 0.002 |
| rs2431424 | 178728965 | G | C | INTRON | NA | 0.025 |
| . | 178729086 | A | G | INTRON | NA |  |
| . | 178729188 | T | C | INTRON | NA |  |
| rs74494693 | 178729356 | C | T | INTRON | NA | 0.083 |
| . | 178729792 | A | G | INTRON | NA |  |
| rs2431423 | 178730062 | T | G | INTRON | NA | 0.243 |
| rs2450197 | 178730638 | T | A | INTRON | NA | 0.244 |
| . | 178731071 | C | T | INTRON | NA |  |
| rs1961716 | 178731921 | C | T | INTRON | NA | 0.203 |
| rs2431422 | 178731950 | G | A | INTRON | NA | 0.198 |
| rs10074922 | 178732482 | T | C | INTRON | NA | 0.178 |
| rs6892931 | 178733281 | T | C | INTRON | NA |  |
| rs462925 | 178734092 | C | T | INTRON | NA | 0.009 |
| rs55773105 | 178734392 | A | C | INTRON | NA | 0.087 |
| . | 178734517 | G | A | INTRON | NA |  |
| rs114827265 | 178734931 | C | A | INTRON | NA | 0.017 |
| rs338879 | 178735068 | A | G | INTRON | NA | 0.197 |
| rs11749917 | 178735677 | A | G | INTRON | NA | 0.035 |
| rs146235175 | 178735994 | C | T | INTRON | NA | 0.011 |
| rs115178105 | 178736022 | G | A | INTRON | NA | 0.030 |
| rs112361927 | 178736252 | G | A | INTRON | NA | 0.003 |
| rs11750794 | 178736595 | A | G | INTRON | NA | 0.025 |
| rs115777327 | 178736629 | C | T | INTRON | NA | 0.044 |
| rs151076594 | 178736749 | C | A | INTRON | NA | 0.012 |
| rs62395027 | 178737132 | C | T | INTRON | NA | 0.025 |
| rs11738729 | 178737516 | A | T | INTRON | NA | 0.045 |
| rs116462352 | 178737568 | T | A | INTRON | NA | 0.008 |
| rs878932 | 178737752 | T | C | INTRON | NA | 0.278 |
| rs173558 | 178737948 | C | A | INTRON | NA | 0.173 |
| rs62395028 | 178738284 | T | C | INTRON | NA | 0.024 |
| rs13179536 | 178738392 | G | T | INTRON | NA | 0.124 |
| rs62395029 | 178738437 | A | G | INTRON | NA | 0.027 |
| rs463868 | 178738578 | T | C | INTRON | NA | 0.085 |
| rs878933 | 178739016 | G | A | INTRON | NA | 0.169 |
| rs11743472 | 178739541 | G | A | INTRON | NA | 0.021 |
| . | 178739991 | G | A | INTRON | NA |  |
| rs248180 | 178740070 | C | T | INTRON | NA | 0.193 |
| . | 178740224 | C | T | INTRON | NA |  |
| rs1683633 | 178741117 | A | G | INTRON | NA | 0.256 |
| rs11745264 | 178741383 | G | A | INTRON | NA | 0.030 |
| rs112639528 | 178741408 | C | T | INTRON | NA | 0.002 |
| . | 178741791 | T | C | INTRON | NA |  |
| rs113444657 | 178741885 | G | A | INTRON | NA | 0.004 |
| rs76025810 | 178742521 | A | G | INTRON | NA | 0.047 |
| rs340113 | 178742614 | C | T | INTRON | NA | 0.103 |
| rs340114 | 178742625 | C | G | INTRON | NA | 0.054 |
| rs6888230 | 178742761 | G | A | INTRON | NA | 0.376 |
| rs78644397 | 178742769 | T | C | INTRON | NA | 0.047 |
| rs75841589 | 178743158 | A | G | INTRON | NA | 0.047 |
| rs77255290 | 178743177 | G | A | INTRON | NA | 0.007 |
| rs58665820 | 178743281 | G | A | INTRON | NA | 0.173 |
| rs72820642 | 178743307 | G | C | INTRON | NA | 0.007 |
| rs80123113 | 178743348 | A | G | INTRON | NA | 0.047 |
| rs116261332 | 178743423 | C | A | INTRON | NA | 0.017 |
| rs185299 | 178743627 | G | A | INTRON | NA | 0.168 |
| rs55736255 | 178744089 | G | A | INTRON | NA | 0.030 |
| rs6884587 | 178744172 | T | C | INTRON | NA | 0.053 |
| rs80227607 | 178744532 | C | A | INTRON | NA | 0.001 |
| rs340115 | 178744798 | C | T | INTRON | NA | 0.051 |
| rs41122 | 178744959 | G | A | INTRON | NA | 0.428 |
| rs77409252 | 178745573 | C | T | INTRON | NA | 0.043 |
| rs34418943 | 178745970 | C | T | INTRON | NA | 0.022 |
| rs117958765 | 178746342 | C | A | INTRON | NA | 0.014 |
| rs72820643 | 178746376 | G | A | INTRON | NA | 0.016 |
| rs163321 | 178746447 | A | G | INTRON | NA | 0.458 |
| rs12153310 | 178746816 | G | A | INTRON | NA | 0.158 |
| rs56033019 | 178746897 | C | T | INTRON | NA | 0.006 |
| rs163322 | 178747015 | T | C | INTRON | NA | 0.446 |
| . | 178747079 | C | T | INTRON | NA |  |
| rs186340004 | 178747182 | C | T | INTRON | NA | 0.003 |
| . | 178747340 | G | A | INTRON | NA |  |
| rs2036688 | 178747387 | C | T | INTRON | NA | 0.147 |
| rs460892 | 178747468 | C | G | INTRON | NA | 0.414 |
| . | 178747571 | A | G | INTRON | NA |  |
| rs180289 | 178747612 | C | T | INTRON | NA | 0.256 |
| rs72820646 | 178747739 | T | C | INTRON | NA | 0.029 |
| rs163497 | 178747939 | C | T | INTRON | NA | 0.236 |
| rs23438 | 178748332 | G | A | INTRON | NA | 0.279 |
| rs72820647 | 178748351 | C | T | INTRON | NA | 0.012 |
| rs186872 | 178748547 | T | C | INTRON | NA | 0.140 |
| rs456356 | 178748645 | T | C | INTRON | NA | 0.236 |
| . | 178748679 | G | A | INTRON | NA |  |
| rs163498 | 178748987 | A | G | INTRON | NA | 0.379 |
| rs116762514 | 178749074 | G | A | INTRON | NA | 0.002 |
| rs34750498 | 178749082 | C | G | INTRON | NA |  |
| . | 178749255 | C | T | INTRON | NA |  |
| rs457432 | 178749303 | A | G | INTRON | NA | 0.099 |
| rs148215671 | 178749338 | C | T | INTRON | NA | 0.007 |
| . | 178749390 | G | A | INTRON | NA |  |
| rs114962475 | 178749398 | T | C | INTRON | NA | 0.007 |
| rs185160292 | 178749448 | G | A | INTRON | NA | 0.000 |
| rs79719856 | 178749572 | G | A | INTRON | NA | 0.068 |
| rs459106 | 178749661 | A | G | INTRON | NA | 0.427 |
| . | 178749675 | A | G | INTRON | NA |  |
| rs464610 | 178749813 | G | A | INTRON | NA | 0.463 |
| rs455437 | 178749829 | A | G | INTRON | NA | 0.159 |
| rs139779696 | 178749851 | G | A | INTRON | NA | 0.002 |
| rs458925 | 178749898 | T | G | INTRON | NA | 0.402 |
| rs408508 | 178750140 | G | C | INTRON | NA | 0.140 |
| rs420843 | 178750256 | C | T | INTRON | NA | 0.132 |
| rs422172 | 178750377 | T | C | INTRON | NA | 0.418 |
| . | 178750470 | G | A | INTRON | NA |  |
| rs368318 | 178750511 | A | T | INTRON | NA | 0.425 |
| rs457316 | 178750656 | C | G | INTRON | NA | 0.477 |
| rs463635 | 178750679 | G | A | INTRON | NA | 0.426 |
| rs72820654 | 178750865 | T | A | INTRON | NA | 0.007 |
| rs2054781 | 178751269 | T | C | INTRON | NA | 0.422 |
| rs145188007 | 178751553 | G | A | INTRON | NA | 0.003 |
| rs4701095 | 178752189 | C | T | INTRON | NA | 0.112 |
| rs116266446 | 178752262 | G | A | INTRON | NA | 0.003 |
| rs72648841 | 178752312 | G | T | INTRON | NA | 0.253 |
| rs112153047 | 178752809 | T | C | INTRON | NA | 0.071 |
| rs72648842 | 178753037 | C | T | INTRON | NA | 0.260 |
| rs185905168 | 178753480 | C | T | INTRON | NA | 0.007 |
| rs13155813 | 178753541 | G | A | INTRON | NA | 0.012 |
| rs68093766 | 178753568 | G | T | INTRON | NA | 0.305 |
| rs146575893 | 178753630 | G | T | INTRON | NA | 0.003 |
| rs57604610 | 178753692 | C | G | INTRON | NA | 0.087 |
| rs116000644 | 178753920 | C | A | INTRON | NA | 0.001 |
| rs62395042 | 178753982 | C | T | INTRON | NA | 0.389 |
| rs59286644 | 178754425 | A | G | INTRON | NA | 0.301 |
| rs11956034 | 178754468 | C | T | INTRON | NA | 0.330 |
| rs4700799 | 178754945 | A | G | INTRON | NA | 0.379 |
| rs11951985 | 178755060 | C | T | INTRON | NA |  |
| . | 178755258 | T | C | INTRON | NA |  |
| rs67515729 | 178755476 | A | G | INTRON | NA | 0.303 |
| rs72648843 | 178755847 | C | T | INTRON | NA | 0.250 |
| rs150080900 | 178756020 | G | A | INTRON | NA | 0.002 |
| rs6892617 | 178756066 | T | C | INTRON | NA | 0.076 |
| rs113146948 | 178756072 | C | T | INTRON | NA | 0.033 |
| rs138076033 | 178756095 | A | G | INTRON | NA | 0.054 |
| rs142536092 | 178756099 | A | C | INTRON | NA | 0.055 |
| . | 178756196 | C | T | INTRON | NA |  |
| rs76493103 | 178756208 | G | A | INTRON | NA | 0.078 |
| rs28618672 | 178756242 | G | C | INTRON | NA | 0.023 |
| rs28556579 | 178756245 | G | T | INTRON | NA | 0.022 |
| rs113751837 | 178756281 | T | C | INTRON | NA | 0.045 |
| . | 178756292 | C | T | INTRON | NA |  |
| rs443941 | 178756557 | T | C | INTRON | NA | 0.417 |
| . | 178756866 | G | A | INTRON | NA |  |
| . | 178756898 | C | T | INTRON | NA |  |
| rs114528210 | 178757480 | G | A | INTRON | NA | 0.004 |
| rs403488 | 178757626 | C | G | INTRON | NA | 0.321 |
| rs138113367 | 178757884 | C | T | INTRON | NA | 0.002 |
| rs62395044 | 178758026 | G | A | INTRON | NA | 0.225 |
| rs34839395 | 178758161 | C | T | INTRON | NA | 0.232 |
| rs2431434 | 178758238 | G | A | INTRON | NA | 0.156 |
| rs340419 | 178758909 | T | C | INTRON | NA | 0.296 |
| rs78060930 | 178759504 | G | C | INTRON | NA | 0.005 |
| rs148515374 | 178759776 | G | A | INTRON | NA | 0.003 |
| rs11745920 | 178759900 | C | T | INTRON | NA | 0.197 |
| rs75231519 | 178760068 | G | A | INTRON | NA | 0.027 |
| rs340418 | 178760954 | A | G | INTRON | NA | 0.120 |
| rs181471248 | 178761145 | G | A | INTRON | NA | 0.002 |
| rs7734278 | 178761358 | G | A | INTRON | NA | 0.471 |
| . | 178761496 | A | G | INTRON | NA |  |
| rs340417 | 178762064 | C | A | INTRON | NA | 0.119 |
| rs3846697 | 178762092 | T | C | INTRON | NA | 0.253 |
| rs59044622 | 178762140 | G | A | INTRON | NA | 0.033 |
| rs56413521 | 178762892 | C | T | INTRON | NA | 0.009 |
| rs411010 | 178763097 | A | G | INTRON | NA | 0.340 |
| rs444735 | 178763108 | C | T | INTRON | NA | 0.356 |
| rs153817 | 178763320 | C | T | INTRON | NA | 0.354 |
| rs114091179 | 178763484 | C | G | INTRON | NA | 0.008 |
| rs153816 | 178763605 | C | T | INTRON | NA | 0.119 |
| rs74884811 | 178763644 | C | T | INTRON | NA | 0.170 |
| rs76653143 | 178763724 | G | A | INTRON | NA | 0.040 |
| . | 178763874 | G | C | INTRON | NA |  |
| rs153814 | 178764482 | T | C | INTRON | NA | 0.371 |
| rs26811 | 178764898 | T | A | INTRON | NA | 0.443 |
| rs26812 | 178764906 | A | G | INTRON | NA | 0.396 |
| rs33900 | 178765138 | T | C | INTRON | NA | 0.351 |
| rs27697 | 178765157 | C | G | INTRON | NA | 0.335 |
| rs28060 | 178765373 | G | A | INTRON | NA | 0.394 |
| rs3843495 | 178765495 | C | T | INTRON | NA | 0.013 |
| rs141872172 | 178765506 | A | G | INTRON | NA | 0.022 |
| rs182397041 | 178765935 | G | A | INTRON | NA | 0.001 |
| rs26813 | 178766209 | T | C | INTRON | NA | 0.179 |
| . | 178766484 | G | C | INTRON | NA |  |
| rs55722639 | 178766570 | G | A | INTRON | NA | 0.226 |
| . | 178766752 | C | T | INTRON | NA |  |
| rs28549 | 178766912 | A | G | INTRON | NA | 0.330 |
| rs7733548 | 178766925 | G | A | INTRON | NA | 0.228 |
| rs7720895 | 178767511 | A | G | INTRON | NA | 0.245 |
| rs117490923 | 178767870 | C | T | INTRON | NA | 0.005 |
| rs33898 | 178767910 | G | A | INTRON | NA | 0.112 |
| rs115561455 | 178768034 | G | A | INTRON | NA | 0.004 |
| rs78032198 | 178768043 | C | G | INTRON | NA | 0.008 |
| rs33897 | 178768312 | C | T | INTRON | NA | 0.142 |
| rs76429249 | 178768401 | G | T | INTRON | NA | 0.177 |
| rs55714836 | 178768439 | T | C | INTRON | NA | 0.255 |
| rs33896 | 178768505 | C | T | INTRON | NA | 0.354 |
| rs3846699 | 178768578 | G | A | INTRON | NA | 0.165 |
| rs340112 | 178768583 | A | G | INTRON | NA | 0.002 |
| rs115300558 | 178768671 | G | A | INTRON | NA | 0.005 |
| . | 178768867 | C | T | INTRON | NA |  |
| rs3889567 | 178768919 | C | T | INTRON | NA | 0.176 |
| rs142128070 | 178769328 | C | T | INTRON | NA | 0.000 |
| rs26814 | 178769518 | A | G | INTRON | NA |  |
| rs75213418 | 178769527 | A | G | INTRON | NA | 0.026 |
| rs7713020 | 178769937 | C | T | INTRON | NA | 0.178 |
| rs7736370 | 178770100 | A | G | INTRON | NA | 0.179 |
| rs4700800 | 178770202 | C | T | INTRON | NA | 0.198 |
| . | 178770213 | A | G | INTRON | NA |  |
| . | 178770240 | A | G | INTRON | NA |  |
| rs4701098 | 178770381 | G | A | INTRON | NA | 0.394 |
| rs115623068 | 178770465 | C | A | INTRON | NA | 0.003 |
| rs12513486 | 178770485 | C | A | INTRON | NA | 0.200 |
| rs2271214 | 178770718 | A | G | INTRON | NA | 0.196 |
| rs2271213 | 178770759 | C | G | INTRON | NA | 0.395 |
| rs2271212 | 178770981 | A | G | SYNONYMOUS_CODING | NA |  |
| rs3892886 | 178772813 | T | C | UPSTREAM | NA | 0.206 |
| rs3892887 | 178772854 | G | A | UPSTREAM | NA | 0.206 |
| . | 178772978 | C | G | UPSTREAM | NA |  |
| rs153813 | 178773041 | T | G | UPSTREAM | NA | 0.468 |
| rs340111 | 178773203 | C | T | UPSTREAM | NA | 0.436 |
| rs3756570 | 178773217 | C | T | UPSTREAM | NA | 0.196 |
| rs3846700 | 178773476 | A | G | UPSTREAM | NA | 0.395 |
| rs3846701 | 178773477 | T | A | UPSTREAM | NA | 0.395 |
| . | 178773607 | G | A | UPSTREAM | NA |  |
| rs3822603 | 178773617 | C | G | UPSTREAM | NA | 0.195 |
| rs4340904 | 178773662 | G | A | UPSTREAM | NA | 0.001 |
| rs255028 | 178773938 | G | A | UPSTREAM | NA |  |
| rs149873293 | 178774237 | C | T | UPSTREAM | NA | 0.001 |
| rs78899330 | 178774463 | A | G | UPSTREAM | NA | 0.024 |
| rs456150 | 178774478 | T | C | UPSTREAM | NA | 0.001 |
| rs255027 | 178774860 | A | G | UPSTREAM | NA | 0.349 |
| rs33903 | 178775199 | G | A | UPSTREAM | NA | 0.020 |
| rs79007950 | 178775492 | G | T | UPSTREAM | NA | 0.004 |
| rs138452822 | 178775817 | G | C | UPSTREAM | NA | 0.012 |
| rs33902 | 178776122 | C | T | UPSTREAM | NA | 0.166 |
| rs33901 | 178776147 | G | C | UPSTREAM | NA | 0.352 |
| rs340110 | 178776822 | C | A | UPSTREAM | NA | 0.404 |
| . | 178776928 | G | A | UPSTREAM | NA |  |
