# Supplementary material for: ADAMTS12, a new candidate gene for pediatric stroke
Source: PLoS One. 2020 Aug 20;15(8):e0237928. doi: 10.1371/journal.pone.0237928 (PMC7446847; doi:10.1371/journal.pone.0237928)
Supplement: S2 Table — Variants detected using NGS based target enrichment in the defined genomic region of ADAMTS12. (DOCX) [file pone.0237928.s003.docx]

**S2 Table. *ADAMTS12* variants.** Variants detected using NGS based target enrichment in the defined genomic region of *ADAMTS12*.

| ID | POS | REF | ALT | EFFECT | SIFT | Global minor allele frequency (all individuals) |
| --- | --- | --- | --- | --- | --- | --- |
| rs12521936 | 33522400 | G | A | DOWNSTREAM | NA | 0.2412 |
| . | 33522678 | C | T | DOWNSTREAM | NA |  |
| rs187560643 | 33523552 | C | T | DOWNSTREAM | NA | 0.0010 |
| rs116613080 | 33523613 | G | T | DOWNSTREAM | NA | 0.0024 |
| rs6872391 | 33524190 | A | G | DOWNSTREAM | NA | 0.0887 |
| rs72737381 | 33524239 | C | T | DOWNSTREAM | NA | 0.0589 |
| . | 33524386 | C | T | DOWNSTREAM | NA |  |
| rs79792805 | 33524787 | G | A | DOWNSTREAM | NA | 0.0060 |
| rs27405 | 33525321 | C | T | DOWNSTREAM | NA | 0.4920 |
| rs27739 | 33526441 | A | T | DOWNSTREAM | NA | 0.2041 |
| rs141034975 | 33526807 | T | C | DOWNSTREAM | NA | 0.0016 |
| . | 33526838 | C | T | DOWNSTREAM | NA |  |
| . | 33527053 | G | A | DOWNSTREAM | NA |  |
| rs256605 | 33528018 | C | T | INTRON | NA | 0.4285 |
| rs72737382 | 33528825 | G | A | INTRON | NA | 0.1106 |
| rs150799292 | 33528892 | C | T | INTRON | NA | 0.0583 |
| rs256604 | 33528909 | T | C | INTRON | NA | 0.4946 |
| rs10521004 | 33529263 | T | C | INTRON | NA | 0.1234 |
| rs7702210 | 33529398 | C | T | INTRON | NA | 0.0743 |
| rs7722715 | 33529783 | T | G | INTRON | NA | 0.0553 |
| . | 33530385 | A | G | INTRON | NA |  |
| rs256603 | 33530529 | C | T | INTRON | NA | 0.4002 |
| rs256602 | 33530556 | C | T | INTRON | NA | 0.3349 |
| rs256601 | 33530746 | A | C | INTRON | NA | 0.3912 |
| rs78625068 | 33531100 | G | A | INTRON | NA |  |
| rs116799890 | 33531128 | T | C | INTRON | NA | 0.0128 |
| rs256792 | 33531645 | C | T | INTRON | NA | 0.4950 |
| rs78350456 | 33531804 | C | A | INTRON | NA | 0.0030 |
| rs27675 | 33531875 | G | A | INTRON | NA | 0.4006 |
| rs25750 | 33532288 | C | T | INTRON | NA | 0.4006 |
| rs142689957 | 33532497 | T | C | INTRON | NA | 0.0040 |
| rs25751 | 33532562 | C | T | INTRON | NA | 0.4004 |
| rs25752 | 33532596 | C | G | INTRON | NA |  |
| rs25753 | 33532843 | A | G | INTRON | NA | 0.3958 |
| rs457935 | 33533373 | C | T | INTRON | NA | 0.4006 |
| rs72737388 | 33533432 | A | G | INTRON | NA | 0.0553 |
| rs27975 | 33533679 | T | C | INTRON | NA | 0.3838 |
| rs28100 | 33533865 | C | G | INTRON | NA | 0.4625 |
| rs25754 | 33535060 | G | A | NON_SYNONYMOUS_CODING | 0.41 | 0.4004 |
| rs66492429 | 33535135 | C | T | INTRON | NA | 0.0148 |
| rs6865176 | 33535393 | C | T | INTRON | NA | 0.0553 |
| . | 33535416 | C | T | INTRON | NA |  |
| rs457353 | 33535571 | G | T | INTRON | NA | 0.4004 |
| rs2277039 | 33535802 | A | G | INTRON | NA | 0.0547 |
| rs25755 | 33535814 | T | G | INTRON | NA | 0.2067 |
| rs25756 | 33536033 | T | C | INTRON | NA | 0.3956 |
| rs27850 | 33536228 | G | A | INTRON | NA | 0.4004 |
| rs29969 | 33536291 | A | G | INTRON | NA | 0.3636 |
| rs27404 | 33536616 | G | C | INTRON | NA | 0.3912 |
| . | 33537618 | T | A | INTRON | NA |  |
| rs17566960 | 33537702 | G | A | INTRON | NA | 0.0150 |
| rs149247027 | 33538165 | G | A | INTRON | NA | 0.0018 |
| rs154219 | 33538328 | A | G | INTRON | NA | 0.3337 |
| rs256641 | 33538528 | A | C | INTRON | NA | 0.4976 |
| rs256642 | 33539171 | G | C | INTRON | NA | 0.4173 |
| rs149777833 | 33539172 | G | A | INTRON | NA | 0.0679 |
| rs256643 | 33539197 | C | T | INTRON | NA | 0.4545 |
| rs256644 | 33539291 | C | A | INTRON | NA | 0.4545 |
| rs17488979 | 33539428 | T | C | INTRON | NA | 0.0042 |
| . | 33539516 | A | G | INTRON | NA |  |
| rs72737392 | 33539762 | C | T | INTRON | NA | 0.0553 |
| rs256646 | 33540093 | G | C | INTRON | NA | 0.3614 |
| rs171759 | 33540418 | A | G | INTRON | NA | 0.4549 |
| rs185366 | 33540446 | C | A | INTRON | NA | 0.4171 |
| rs256647 | 33540452 | A | G | INTRON | NA | 0.4545 |
| rs75610429 | 33540670 | C | T | INTRON | NA |  |
| rs112679346 | 33541427 | T | A | INTRON | NA | 0.0647 |
| rs464719 | 33541527 | C | T | INTRON | NA | 0.4545 |
| rs368704 | 33542704 | A | G | INTRON | NA | 0.4173 |
| . | 33545965 | G | A | INTRON | NA |  |
| rs61753559 | 33546207 | T | C | NON_SYNONYMOUS_CODING | 0.12 | 0.0010 |
| rs29967 | 33546432 | A | G | INTRON | NA | 0.3918 |
| rs72737394 | 33546491 | A | G | INTRON | NA | 0.0607 |
| . | 33547400 | T | C | INTRON | NA |  |
| rs29968 | 33547787 | A | G | INTRON | NA | 0.4856 |
| . | 33548247 | G | A | INTRON | NA |  |
| rs190460020 | 33548248 | C | G | INTRON | NA | 0.0014 |
| rs1501724 | 33549014 | T | C | INTRON | NA | 0.1901 |
| rs114689616 | 33549031 | C | G | INTRON | NA | 0.0100 |
| rs465015 | 33549118 | G | A | INTRON | NA |  |
| rs112196098 | 33549350 | C | G | NON_SYNONYMOUS_CODING | 1 | 0.0076 |
| rs77581578 | 33549374 | G | T | NON_SYNONYMOUS_CODING | 0.01 | 0.0076 |
| rs61748195 | 33549387 | G | A | SYNONYMOUS_CODING | NA | 0.0437 |
| rs462629 | 33549559 | C | A | INTRON | NA | 0.3285 |
| rs102940 | 33550124 | A | G | INTRON | NA | 0.4724 |
| rs755368 | 33550170 | C | T | INTRON | NA | 0.0080 |
| rs192478929 | 33551010 | C | T | INTRON | NA | 0.0008 |
| . | 33551122 | C | T | INTRON | NA |  |
| rs696155 | 33552040 | G | A | INTRON | NA | 0.3580 |
| rs545861 | 33552341 | T | C | INTRON | NA | 0.4730 |
| rs184210547 | 33552475 | C | T | INTRON | NA | 0.0012 |
| rs457359 | 33552829 | C | T | INTRON | NA | 0.3055 |
| rs62351099 | 33552851 | G | A | INTRON | NA | 0.0330 |
| . | 33554140 | A | G | INTRON | NA |  |
| rs149220092 | 33555691 | T | C | INTRON | NA |  |
| rs116551687 | 33555749 | G | A | INTRON | NA | 0.0054 |
| rs116045300 | 33556163 | G | A | INTRON | NA | 0.0022 |
| rs154218 | 33556340 | A | T | INTRON | NA | 0.0014 |
| rs256640 | 33556346 | A | G | INTRON | NA | 0.3063 |
| rs256639 | 33556366 | G | A | INTRON | NA | 0.3710 |
| rs154230 | 33556690 | T | C | INTRON | NA | 0.3063 |
| rs168888 | 33556949 | G | A | INTRON | NA | 0.3886 |
| rs79078288 | 33557242 | A | T | INTRON | NA | 0.0114 |
| rs256638 | 33557960 | C | T | INTRON | NA | 0.3884 |
| . | 33557988 | A | G | INTRON | NA |  |
| rs256637 | 33557999 | G | A | INTRON | NA | 0.4012 |
| . | 33558439 | T | C | INTRON | NA |  |
| rs194381 | 33558544 | C | T | INTRON | NA | 0.2959 |
| rs75818459 | 33559104 | T | C | INTRON | NA | 0.0176 |
| rs113163214 | 33559132 | C | T | INTRON | NA | 0.0068 |
| rs62351101 | 33559410 | G | A | INTRON | NA | 0.1364 |
| rs35585640 | 33559705 | G | A | INTRON | NA | 0.1821 |
| rs142040856 | 33559733 | C | T | INTRON | NA | 0.0030 |
| rs145326128 | 33559878 | C | T | INTRON | NA | 0.0110 |
| rs371394 | 33560028 | G | C | INTRON | NA | 0.3592 |
| rs10447123 | 33560395 | C | A | INTRON | NA | 0.0156 |
| rs696837 | 33560623 | A | T | INTRON | NA | 0.2951 |
| rs861768 | 33560834 | A | T | INTRON | NA | 0.2951 |
| . | 33562139 | C | G | INTRON | NA |  |
| rs17489325 | 33562360 | A | G | INTRON | NA | 0.2550 |
| rs16891329 | 33562451 | C | T | INTRON | NA | 0.0725 |
| rs17567498 | 33563744 | C | T | INTRON | NA | 0.1831 |
| rs1501727 | 33563980 | T | C | INTRON | NA | 0.3045 |
| . | 33564222 | G | A | INTRON | NA |  |
| rs4146150 | 33565137 | C | T | INTRON | NA | 0.4948 |
| rs35283710 | 33565406 | A | G | INTRON | NA | 0.1875 |
| rs2016646 | 33565995 | A | G | INTRON | NA | 0.1711 |
| rs700934 | 33566174 | G | A | INTRON | NA | 0.2354 |
| rs13158954 | 33566653 | G | T | INTRON | NA | 0.1829 |
| . | 33566800 | A | G | INTRON | NA |  |
| rs114922228 | 33567331 | G | A | INTRON | NA | 0.0130 |
| rs182478188 | 33568496 | G | A | INTRON | NA | 0.0008 |
| rs11742406 | 33568521 | G | C | INTRON | NA | 0.2877 |
| rs11742409 | 33568544 | G | T | INTRON | NA | 0.2919 |
| . | 33568772 | G | A | INTRON | NA |  |
| rs190251430 | 33568885 | G | A | INTRON | NA | 0.0014 |
| rs143922286 | 33569569 | G | A | INTRON | NA |  |
| rs151262246 | 33571667 | C | G | INTRON | NA | 0.2955 |
| rs140510395 | 33571668 | C | A | INTRON | NA | 0.2953 |
| rs11747983 | 33574591 | C | G | INTRON | NA | 0.2610 |
| . | 33575327 | C | T | INTRON | NA |  |
| . | 33575866 | C | T | INTRON | NA |  |
| . | 33576135 | G | A | INTRON | NA |  |
| rs3813474 | 33576602 | A | G | NON_SYNONYMOUS_CODING | 0.91 | 0.0513 |
| . | 33577548 | G | A | INTRON | NA |  |
| rs16891332 | 33578083 | T | C | INTRON | NA | 0.0913 |
| rs79174796 | 33578190 | G | A | INTRON | NA | 0.0647 |
| rs62351123 | 33578295 | T | A | INTRON | NA | 0.1512 |
| rs10042589 | 33578441 | G | A | INTRON | NA | 0.2326 |
| rs256651 | 33578470 | C | T | INTRON | NA | 0.2059 |
| rs140069426 | 33578530 | G | A | INTRON | NA | 0.0014 |
| rs16891337 | 33578638 | A | G | INTRON | NA | 0.0933 |
| rs75396177 | 33578854 | C | G | INTRON | NA | 0.0439 |
| rs256650 | 33579126 | G | A | INTRON | NA | 0.1841 |
| rs256649 | 33579193 | A | G | INTRON | NA | 0.1851 |
| rs460845 | 33579253 | G | C | INTRON | NA | 0.1881 |
| rs460532 | 33579255 | G | A | INTRON | NA | 0.1883 |
| rs187672 | 33579291 | C | T | INTRON | NA | 0.1937 |
| rs6866077 | 33579395 | T | C | INTRON | NA | 0.1885 |
| rs750803 | 33579523 | T | C | INTRON | NA | 0.3994 |
| rs6885236 | 33579543 | C | T | INTRON | NA | 0.1883 |
| rs420356 | 33579570 | T | C | INTRON | NA | 0.1927 |
| rs435783 | 33579812 | T | C | INTRON | NA | 0.1925 |
| rs141154571 | 33579825 | A | T | INTRON | NA | 0.0008 |
| rs385599 | 33579935 | G | T | INTRON | NA | 0.1923 |
| rs385224 | 33580053 | G | T | INTRON | NA | 0.1913 |
| rs79082973 | 33580119 | C | T | INTRON | NA | 0.0437 |
| rs384821 | 33580181 | G | A | INTRON | NA | 0.1915 |
| rs380548 | 33580204 | C | T | INTRON | NA | 0.1913 |
| rs365376 | 33580431 | T | C | INTRON | NA | 0.1913 |
| rs112072724 | 33581303 | C | T | INTRON | NA | 0.0028 |
| rs97795 | 33581630 | G | A | INTRON | NA | 0.4563 |
| rs62351125 | 33581853 | G | C | INTRON | NA | 0.0012 |
| rs99629 | 33582050 | G | A | INTRON | NA | 0.4091 |
| rs6861377 | 33582157 | C | G | INTRON | NA | 0.4213 |
| rs146795088 | 33582636 | T | C | INTRON | NA | 0.0014 |
| rs1690973 | 33583064 | T | C | INTRON | NA | 0.4026 |
| . | 33583398 | T | A | INTRON | NA |  |
| rs2642667 | 33584224 | T | C | INTRON | NA | 0.4066 |
| rs1846979 | 33584560 | T | C | INTRON | NA | 0.4567 |
| rs428052 | 33585401 | G | A | INTRON | NA | 0.4083 |
| . | 33585473 | C | T | INTRON | NA |  |
| rs408758 | 33585754 | T | C | INTRON | NA | 0.3355 |
| rs72737399 | 33585768 | C | T | INTRON | NA | 0.0437 |
| rs441175 | 33586003 | T | C | INTRON | NA | 0.4573 |
| rs409592 | 33586031 | T | C | INTRON | NA | 0.4069 |
| rs10072202 | 33586037 | A | G | INTRON | NA | 0.0106 |
| rs7715688 | 33586255 | T | A | INTRON | NA | 0.1567 |
| rs148858210 | 33587695 | G | C | INTRON | NA | 0.0042 |
| rs411690 | 33587804 | T | G | INTRON | NA | 0.2063 |
| rs418591 | 33587842 | T | G | INTRON | NA | 0.2067 |
| rs436415 | 33588070 | A | G | INTRON | NA | 0.2065 |
| rs818726 | 33588146 | A | G | INTRON | NA | 0.4535 |
| rs114523844 | 33588237 | G | A | INTRON | NA | 0.0124 |
| rs818725 | 33588303 | C | G | INTRON | NA | 0.1963 |
| rs429419 | 33588335 | C | G | INTRON | NA | 0.2061 |
| rs2453289 | 33588416 | A | G | INTRON | NA | 0.3968 |
| rs115917866 | 33588483 | C | G | INTRON | NA | 0.0040 |
| rs141518800 | 33588556 | C | T | INTRON | NA | 0.0054 |
| rs4242083 | 33589017 | A | G | INTRON | NA | 0.0729 |
| rs78068038 | 33589038 | A | T | INTRON | NA | 0.0042 |
| . | 33589043 | C | T | INTRON | NA |  |
| . | 33589136 | T | C | INTRON | NA |  |
| rs17568177 | 33589349 | A | T | INTRON | NA | 0.2069 |
| rs74983142 | 33589844 | T | C | INTRON | NA | 0.0619 |
| . | 33589986 | T | G | INTRON | NA |  |
| rs6878028 | 33590675 | G | A | INTRON | NA | 0.0138 |
| rs6882270 | 33590968 | C | A | INTRON | NA | 0.1645 |
| rs16891371 | 33591433 | A | G | INTRON | NA | 0.2296 |
| rs2331242 | 33591897 | A | T | INTRON | NA | 0.4517 |
| rs140583764 | 33592033 | C | T | INTRON | NA | 0.0100 |
| rs113349612 | 33592330 | G | A | INTRON | NA | 0.0613 |
| rs4538612 | 33592456 | T | C | INTRON | NA | 0.4950 |
| rs12519217 | 33592803 | T | A | INTRON | NA | 0.4946 |
| rs35496999 | 33593699 | A | G | INTRON | NA | 0.1721 |
| rs145158028 | 33593789 | T | C | INTRON | NA | 0.0040 |
| . | 33594073 | C | G | INTRON | NA |  |
| . | 33594479 | T | C | INTRON | NA |  |
| rs7730682 | 33594566 | T | C | INTRON | NA | 0.2105 |
| rs13185934 | 33594778 | C | T | INTRON | NA | 0.2508 |
| rs12188767 | 33594916 | T | C | INTRON | NA | 0.3201 |
| rs76287253 | 33594927 | A | G | INTRON | NA | 0.0024 |
| rs12188768 | 33594942 | T | C | INTRON | NA | 0.2119 |
| rs58847269 | 33595473 | C | T | INTRON | NA | 0.2123 |
| . | 33595546 | C | T | INTRON | NA |  |
| rs16891385 | 33595689 | T | C | INTRON | NA | 0.1016 |
| rs13153550 | 33595803 | C | T | INTRON | NA | 0.4239 |
| rs140874483 | 33595914 | C | G | INTRON | NA | 0.0024 |
| rs10461924 | 33595996 | C | T | INTRON | NA | 0.4643 |
| rs10461925 | 33596193 | A | G | INTRON | NA | 0.4778 |
| rs77324891 | 33596352 | G | A | INTRON | NA | 0.0359 |
| . | 33596849 | C | T | INTRON | NA |  |
| rs4601049 | 33596876 | G | C | INTRON | NA | 0.4882 |
| . | 33596959 | G | A | INTRON | NA |  |
| rs4437376 | 33597239 | A | T | INTRON | NA | 0.4730 |
| rs4407634 | 33597403 | C | T | INTRON | NA | 0.4730 |
| rs4376270 | 33597456 | G | A | INTRON | NA | 0.4730 |
| rs4409097 | 33597559 | C | T | INTRON | NA | 0.4732 |
| rs13163888 | 33597624 | C | G | INTRON | NA | 0.4730 |
| rs78153794 | 33597723 | C | A | INTRON | NA | 0.0126 |
| rs12697290 | 33597841 | T | A | INTRON | NA | 0.2043 |
| rs13164117 | 33597890 | A | G | INTRON | NA | 0.4786 |
| rs12697291 | 33598017 | G | A | INTRON | NA | 0.2224 |
| rs10075345 | 33598302 | A | G | INTRON | NA | 0.4992 |
| rs10060471 | 33598328 | C | T | INTRON | NA | 0.4994 |
| rs12234073 | 33598344 | A | G | INTRON | NA | 0.4796 |
| rs6892916 | 33598499 | C | G | INTRON | NA | 0.0218 |
| rs6893575 | 33598685 | G | A | INTRON | NA | 0.0218 |
| rs6893739 | 33598798 | G | A | INTRON | NA | 0.0218 |
| rs1423535 | 33598909 | A | G | INTRON | NA | 0.2915 |
| rs1423536 | 33599157 | T | C | INTRON | NA | 0.4976 |
| rs13184210 | 33599685 | C | T | INTRON | NA | 0.4976 |
| rs13184224 | 33599761 | A | G | INTRON | NA | 0.4970 |
| rs13184759 | 33599797 | G | A | INTRON | NA | 0.4976 |
| rs13171234 | 33600365 | T | C | INTRON | NA | 0.4874 |
| rs13189065 | 33600465 | A | G | INTRON | NA | 0.4860 |
| rs2113042 | 33600490 | G | A | INTRON | NA | 0.4748 |
| rs2113043 | 33600633 | A | G | INTRON | NA | 0.1959 |
| rs2113044 | 33600634 | T | C | INTRON | NA | 0.4880 |
| rs5025643 | 33600647 | T | A | INTRON | NA | 0.0218 |
| rs7725296 | 33600753 | T | C | INTRON | NA | 0.2630 |
| rs143319971 | 33600821 | A | G | INTRON | NA | 0.0040 |
| rs13190167 | 33600848 | G | A | INTRON | NA | 0.4880 |
| rs115232625 | 33600977 | C | T | INTRON | NA | 0.0136 |
| rs13156059 | 33601099 | A | T | INTRON | NA | 0.4663 |
| rs13156200 | 33601141 | C | A | INTRON | NA | 0.4880 |
| rs9292500 | 33601883 | T | A | INTRON | NA | 0.2915 |
| rs1035506 | 33602260 | T | C | INTRON | NA | 0.3818 |
| rs1423538 | 33602461 | A | T | INTRON | NA | 0.4119 |
| rs1423537 | 33602605 | C | G | INTRON | NA | 0.4335 |
| rs10521005 | 33602714 | A | G | INTRON | NA | 0.4335 |
| rs10472225 | 33602793 | T | C | INTRON | NA | 0.4335 |
| rs10521006 | 33602891 | G | C | INTRON | NA | 0.1987 |
| rs6861273 | 33603035 | T | C | INTRON | NA | 0.0218 |
| rs10472872 | 33603075 | G | A | INTRON | NA | 0.4343 |
| rs10472226 | 33603129 | G | T | INTRON | NA | 0.4333 |
| rs1559264 | 33603231 | G | A | INTRON | NA | 0.4341 |
| rs9292501 | 33603410 | G | A | INTRON | NA | 0.4321 |
| rs10071294 | 33603684 | C | T | INTRON | NA | 0.4327 |
| rs9292502 | 33603759 | G | A | INTRON | NA | 0.4327 |
| rs10071393 | 33603795 | C | G | INTRON | NA | 0.4327 |
| rs12653860 | 33604140 | A | G | INTRON | NA | 0.3556 |
| . | 33604214 | A | C | INTRON | NA |  |
| rs12653881 | 33604249 | A | T | INTRON | NA | 0.3556 |
| rs12651898 | 33604290 | T | G | INTRON | NA | 0.3556 |
| rs77410302 | 33604322 | A | G | INTRON | NA | 0.0218 |
| rs62351141 | 33604361 | A | G | INTRON | NA | 0.1294 |
| rs150588509 | 33604537 | T | C | INTRON | NA | 0.0218 |
| rs11741435 | 33604804 | A | G | INTRON | NA | 0.3556 |
| rs77926777 | 33605223 | C | T | INTRON | NA | 0.0218 |
| rs17665461 | 33605252 | C | T | INTRON | NA | 0.0765 |
| rs181271622 | 33605351 | G | C | INTRON | NA | 0.0042 |
| . | 33605512 | T | C | INTRON | NA |  |
| rs72739415 | 33605680 | T | C | INTRON | NA | 0.0090 |
| rs4495187 | 33605740 | T | C | INTRON | NA | 0.3556 |
| rs113334547 | 33605755 | A | G | INTRON | NA | 0.0164 |
| rs4549533 | 33605858 | C | T | INTRON | NA | 0.4181 |
| rs4469200 | 33605920 | C | A | INTRON | NA | 0.3556 |
| rs4345323 | 33606084 | A | G | INTRON | NA | 0.3556 |
| rs10077285 | 33606344 | C | T | INTRON | NA | 0.3556 |
| rs4355529 | 33606636 | C | G | INTRON | NA | 0.3556 |
| rs4560551 | 33606751 | T | C | INTRON | NA | 0.3556 |
| rs74849569 | 33606824 | T | C | INTRON | NA | 0.0154 |
| rs4437378 | 33606846 | A | G | INTRON | NA | 0.3556 |
| rs4440363 | 33607001 | T | C | INTRON | NA | 0.3556 |
| . | 33607004 | A | T | INTRON | NA |  |
| rs6872736 | 33607031 | A | G | INTRON | NA | 0.0425 |
| rs6873674 | 33607371 | G | A | INTRON | NA | 0.3556 |
| rs6873693 | 33607397 | G | T | INTRON | NA | 0.3556 |
| rs6895257 | 33607537 | T | C | INTRON | NA | 0.3554 |
| rs4432892 | 33607710 | T | C | INTRON | NA |  |
| rs4446461 | 33608207 | A | G | INTRON | NA | 0.3556 |
| rs4469201 | 33608479 | C | G | INTRON | NA | 0.3558 |
| rs114261179 | 33609053 | G | C | INTRON | NA | 0.0218 |
| rs12697292 | 33609352 | C | G | INTRON | NA | 0.3558 |
| rs13153272 | 33609380 | T | C | INTRON | NA | 0.0687 |
| . | 33609426 | A | G | INTRON | NA |  |
| . | 33609488 | T | C | INTRON | NA |  |
| rs13353882 | 33609489 | T | C | INTRON | NA | 0.4621 |
| rs12697293 | 33609617 | A | G | INTRON | NA | 0.3351 |
| rs62351143 | 33609900 | C | T | INTRON | NA | 0.1795 |
| rs62351144 | 33610022 | T | C | INTRON | NA | 0.1581 |
| rs13176047 | 33610071 | G | A | INTRON | NA | 0.0308 |
| rs13175788 | 33610080 | C | T | INTRON | NA | 0.1979 |
| rs59978630 | 33610281 | T | C | INTRON | NA | 0.0425 |
| rs13176570 | 33610356 | G | A | INTRON | NA | 0.1759 |
| . | 33610502 | A | G | INTRON | NA |  |
| rs7729905 | 33610529 | A | G | INTRON | NA | 0.3349 |
| rs6451000 | 33610747 | G | T | INTRON | NA | 0.3133 |
| rs182667245 | 33610849 | G | A | INTRON | NA | 0.0002 |
| rs72739422 | 33611090 | G | A | INTRON | NA | 0.1284 |
| rs187358787 | 33611205 | A | G | INTRON | NA | 0.0016 |
| rs61633180 | 33611253 | A | C | INTRON | NA | 0.0218 |
| rs58610120 | 33611334 | A | C | INTRON | NA | 0.0427 |
| . | 33611419 | G | A | INTRON | NA |  |
| rs73080378 | 33611498 | A | C | INTRON | NA | 0.0427 |
| . | 33611685 | G | A | INTRON | NA |  |
| rs62351145 | 33611751 | G | C | INTRON | NA | 0.1709 |
| rs193018452 | 33611793 | A | T | INTRON | NA | 0.0028 |
| rs80304680 | 33611942 | T | C | INTRON | NA | 0.0695 |
| rs60741973 | 33612091 | G | A | INTRON | NA | 0.0423 |
| rs10941071 | 33612335 | C | T | INTRON | NA | 0.1971 |
| rs76844553 | 33612535 | G | A | INTRON | NA | 0.0224 |
| rs58085989 | 33612717 | A | G | INTRON | NA | 0.2288 |
| rs189573320 | 33613164 | T | C | INTRON | NA | 0.0014 |
| rs4421110 | 33614078 | T | C | INTRON | NA | 0.1302 |
| rs79210190 | 33614131 | C | T | INTRON | NA | 0.0262 |
| rs76139237 | 33614207 | G | A | INTRON | NA | 0.0264 |
| rs75853185 | 33614284 | A | G | INTRON | NA | 0.0262 |
| rs79306594 | 33614292 | A | G | INTRON | NA | 0.0262 |
| rs61748197 | 33614467 | C | A | SYNONYMOUS_CODING | NA | 0.0262 |
| rs80238766 | 33614500 | G | T | INTRON | NA | 0.0260 |
| rs28542423 | 33614729 | T | C | INTRON | NA | 0.2592 |
| . | 33615096 | C | T | INTRON | NA |  |
| rs13165202 | 33615306 | C | A | INTRON | NA | 0.1801 |
| rs55710642 | 33615353 | C | T | INTRON | NA | 0.0833 |
| rs77496541 | 33615398 | C | A | INTRON | NA | 0.0252 |
| rs116392600 | 33615494 | T | C | INTRON | NA | 0.0038 |
| rs183559230 | 33615520 | G | A | INTRON | NA | 0.0008 |
| rs12520271 | 33615552 | A | G | INTRON | NA | 0.2272 |
| . | 33615649 | T | C | INTRON | NA |  |
| rs10057235 | 33615904 | G | A | INTRON | NA | 0.4780 |
| rs4078398 | 33616311 | T | C | INTRON | NA | 0.2967 |
| rs13355994 | 33616606 | C | G | INTRON | NA | 0.1192 |
| . | 33616692 | T | C | INTRON | NA |  |
| rs74427971 | 33616698 | G | A | INTRON | NA | 0.0102 |
| . | 33617110 | C | A | INTRON | NA |  |
| rs4077002 | 33617354 | T | C | INTRON | NA | 0.1789 |
| rs4077001 | 33617565 | C | T | INTRON | NA | 0.2049 |
| rs4077000 | 33617591 | T | C | INTRON | NA | 0.0515 |
| rs9790941 | 33617698 | C | A | INTRON | NA | 0.4978 |
| rs4574547 | 33617767 | A | C | INTRON | NA | 0.1785 |
| . | 33617800 | T | G | INTRON | NA |  |
| rs3924007 | 33617847 | T | C | INTRON | NA | 0.1777 |
| rs13153024 | 33618327 | C | T | INTRON | NA | 0.0589 |
| rs147376115 | 33618330 | A | G | INTRON | NA | 0.0014 |
| rs4401575 | 33618766 | C | A | INTRON | NA | 0.1783 |
| rs72739427 | 33619106 | C | T | INTRON | NA | 0.0084 |
| rs140345148 | 33620007 | C | T | INTRON | NA | 0.0170 |
| rs184392497 | 33620612 | G | C | INTRON | NA | 0.0008 |
| rs7717651 | 33620736 | C | T | INTRON | NA | 0.3337 |
| rs7703871 | 33620887 | T | C | INTRON | NA | 0.2951 |
| rs143236987 | 33621549 | C | A | INTRON | NA | 0.0008 |
| rs9791008 | 33621983 | T | C | INTRON | NA | 0.1723 |
| rs138263548 | 33622061 | A | G | INTRON | NA | 0.0012 |
| rs9791011 | 33622158 | T | C | INTRON | NA | 0.4880 |
| . | 33622264 | G | T | INTRON | NA |  |
| rs182898687 | 33622735 | C | T | INTRON | NA | 0.0074 |
| . | 33622782 | C | T | INTRON | NA |  |
| rs78658744 | 33623076 | T | A | INTRON | NA | 0.0060 |
| rs7715934 | 33623760 | T | G | INTRON | NA | 0.1220 |
| rs13166731 | 33623935 | T | A | INTRON | NA | 0.0857 |
| rs13184524 | 33623985 | C | T | INTRON | NA | 0.0585 |
| rs7710204 | 33624155 | G | C | INTRON | NA | 0.4888 |
| rs61748198 | 33624430 | A | G | SYNONYMOUS_CODING | NA | 0.1607 |
| rs191778214 | 33624475 | G | T | INTRON | NA | 0.0008 |
| rs34333679 | 33624496 | G | A | INTRON | NA | 0.0859 |
| rs4866347 | 33624549 | T | C | INTRON | NA | 0.1811 |
| rs35643428 | 33624947 | G | C | INTRON | NA | 0.1232 |
| rs184263920 | 33624959 | A | G | INTRON | NA | 0.0016 |
| rs34290989 | 33625004 | A | G | INTRON | NA | 0.0861 |
| rs141143914 | 33625107 | T | C | INTRON | NA | 0.0020 |
| rs56269324 | 33625505 | G | A | INTRON | NA | 0.1212 |
| . | 33625513 | C | A | INTRON | NA |  |
| . | 33625662 | C | T | INTRON | NA |  |
| rs72739430 | 33625835 | G | C | INTRON | NA |  |
| rs55847630 | 33626111 | T | C | INTRON | NA | 0.4643 |
| rs74332675 | 33626315 | G | A | INTRON | NA | 0.1270 |
| rs62640991 | 33626461 | G | T | INTRON | NA | 0.0174 |
| rs62640992 | 33626551 | T | C | INTRON | NA | 0.3750 |
| . | 33626672 | T | G | INTRON | NA |  |
| rs111958946 | 33627444 | G | A | INTRON | NA |  |
| rs11742209 | 33628034 | G | A | INTRON | NA | 0.3798 |
| rs10060379 | 33628121 | G | T | INTRON | NA | 0.1350 |
| rs10068310 | 33628171 | T | C | INTRON | NA | 0.3808 |
| rs4242077 | 33628345 | T | A | INTRON | NA | 0.3808 |
| rs35736709 | 33628552 | T | G | INTRON | NA | 0.0719 |
| rs4866403 | 33628808 | A | G | INTRON | NA |  |
| rs4866349 | 33628813 | C | A | INTRON | NA | 0.3800 |
| rs4866350 | 33628836 | C | T | INTRON | NA | 0.2206 |
| rs11749644 | 33629183 | A | G | INTRON | NA | 0.3806 |
| rs67150159 | 33629330 | C | T | INTRON | NA | 0.3804 |
| rs58390113 | 33629431 | T | C | INTRON | NA | 0.3804 |
| rs78395356 | 33629465 | C | A | INTRON | NA | 0.3804 |
| rs56846874 | 33629528 | C | T | INTRON | NA | 0.3808 |
| rs60050221 | 33629726 | C | T | INTRON | NA | 0.3794 |
| rs59142449 | 33629796 | C | T | INTRON | NA | 0.3798 |
| rs57274184 | 33629814 | T | G | INTRON | NA | 0.3796 |
| rs67925815 | 33629903 | C | T | INTRON | NA | 0.3798 |
| rs57005451 | 33629947 | G | T | INTRON | NA | 0.3800 |
| rs57724077 | 33630009 | T | C | INTRON | NA | 0.3800 |
| . | 33630013 | C | G | INTRON | NA |  |
| rs4296807 | 33630643 | A | G | INTRON | NA | 0.1945 |
| rs62351149 | 33630683 | C | T | INTRON | NA | 0.0070 |
| rs4262111 | 33630752 | G | A | INTRON | NA | 0.1911 |
| rs4260678 | 33630839 | A | G | INTRON | NA | 0.3097 |
| rs79948421 | 33631098 | G | A | INTRON | NA | 0.0232 |
| rs112524145 | 33631100 | C | T | INTRON | NA | 0.0114 |
| rs7444287 | 33631142 | C | G | INTRON | NA | 0.3213 |
| rs4435872 | 33631342 | G | A | INTRON | NA | 0.4683 |
| rs4354048 | 33631616 | T | C | INTRON | NA | 0.2754 |
| rs4337859 | 33631739 | C | A | INTRON | NA | 0.2704 |
| rs4639236 | 33631845 | A | G | INTRON | NA | 0.4806 |
| . | 33631997 | A | G | INTRON | NA |  |
| rs72739437 | 33632199 | G | A | INTRON | NA | 0.0779 |
| rs78413334 | 33632341 | A | T | INTRON | NA | 0.0048 |
| rs10941075 | 33632343 | T | C | INTRON | NA | 0.4724 |
| rs75111025 | 33632369 | T | G | INTRON | NA |  |
| rs11959437 | 33632370 | T | G | INTRON | NA | 0.2696 |
| rs10941076 | 33632455 | G | A | INTRON | NA | 0.2702 |
| rs6897038 | 33632717 | T | C | INTRON | NA | 0.2696 |
| rs6451003 | 33632966 | G | A | INTRON | NA |  |
| rs11746951 | 33633034 | G | A | INTRON | NA | 0.0781 |
| rs115273561 | 33633060 | A | C | INTRON | NA | 0.0004 |
| rs6451004 | 33633103 | A | G | INTRON | NA | 0.2698 |
| rs72739439 | 33633213 | T | C | INTRON | NA | 0.0220 |
| rs7720255 | 33633256 | C | T | INTRON | NA | 0.0809 |
| rs6451005 | 33633582 | G | A | INTRON | NA | 0.2708 |
| rs6881181 | 33633614 | C | G | INTRON | NA | 0.2708 |
| rs4866405 | 33633772 | G | C | INTRON | NA | 0.2710 |
| rs4866352 | 33633834 | T | C | INTRON | NA | 0.4798 |
| rs10072973 | 33633921 | G | C | INTRON | NA |  |
| rs10072867 | 33633941 | C | G | INTRON | NA | 0.1709 |
| . | 33634343 | T | C | INTRON | NA |  |
| rs6896805 | 33634560 | A | G | INTRON | NA | 0.2155 |
| rs4358529 | 33634622 | C | T | INTRON | NA | 0.2143 |
| rs12652420 | 33635000 | T | C | INTRON | NA | 0.1278 |
| rs11748713 | 33635100 | C | A | INTRON | NA | 0.2284 |
| rs12110037 | 33635395 | C | T | INTRON | NA | 0.4854 |
| rs6862356 | 33635634 | C | T | INTRON | NA | 0.4836 |
| rs75766187 | 33635901 | T | C | INTRON | NA | 0.0240 |
| rs72739440 | 33636007 | C | T | INTRON | NA | 0.1661 |
| rs13164825 | 33636035 | T | C | INTRON | NA | 0.0971 |
| rs79695603 | 33636058 | G | A | INTRON | NA | 0.0240 |
| rs4380665 | 33636326 | T | G | INTRON | NA | 0.4994 |
| rs6868223 | 33636594 | G | A | INTRON | NA | 0.4900 |
| rs6868084 | 33636670 | C | G | INTRON | NA | 0.4986 |
| rs35529483 | 33637243 | A | G | INTRON | NA | 0.4998 |
| rs57395688 | 33637324 | G | T | INTRON | NA | 0.4986 |
| rs55943381 | 33637396 | T | C | INTRON | NA | 0.4986 |
| . | 33637639 | T | C | INTRON | NA |  |
| rs4076946 | 33637891 | C | A | INTRON | NA | 0.4052 |
| rs4076947 | 33637899 | G | A | INTRON | NA | 0.4052 |
| rs4076944 | 33638224 | A | G | INTRON | NA | 0.4808 |
| rs4242079 | 33638260 | G | A | INTRON | NA | 0.4052 |
| rs4501345 | 33638714 | C | T | INTRON | NA | 0.4054 |
| rs4263503 | 33638872 | T | A | INTRON | NA | 0.4914 |
| rs4076175 | 33639273 | T | A | INTRON | NA | 0.0020 |
| rs73758929 | 33639361 | A | G | INTRON | NA | 0.0294 |
| rs73758930 | 33639362 | C | T | INTRON | NA | 0.0294 |
| rs3935845 | 33639395 | T | A | INTRON | NA | 0.4780 |
| rs62352876 | 33639653 | C | T | INTRON | NA | 0.0929 |
| rs3935844 | 33639682 | G | A | INTRON | NA | 0.4820 |
| . | 33640203 | G | T | INTRON | NA |  |
| rs10074763 | 33640665 | C | T | INTRON | NA | 0.4587 |
| rs4519928 | 33641058 | T | C | INTRON | NA | 0.4577 |
| rs10076809 | 33641422 | G | T | INTRON | NA | 0.0224 |
| rs111507091 | 33641510 | A | G | INTRON | NA | 0.0084 |
| rs13355124 | 33641578 | T | G | INTRON | NA | 0.4575 |
| rs60892887 | 33642163 | G | A | INTRON | NA | 0.1252 |
| rs6861589 | 33642492 | C | T | INTRON | NA | 0.4597 |
| . | 33642550 | C | T | INTRON | NA |  |
| rs6861765 | 33642625 | A | C | INTRON | NA | 0.4599 |
| rs114102312 | 33642954 | T | C | INTRON | NA | 0.0024 |
| rs10065767 | 33643085 | A | G | INTRON | NA | 0.4597 |
| rs10058829 | 33643190 | T | C | INTRON | NA | 0.4595 |
| . | 33643247 | G | A | INTRON | NA |  |
| rs10065974 | 33643308 | A | T | INTRON | NA | 0.4597 |
| rs10066043 | 33643395 | A | T | INTRON | NA | 0.4597 |
| . | 33643431 | C | T | INTRON | NA |  |
| rs61748199 | 33643560 | G | A | SYNONYMOUS_CODING | NA | 0.0507 |
| rs4866410 | 33643960 | G | A | INTRON | NA | 0.4597 |
| . | 33644085 | T | C | INTRON | NA |  |
| rs35388476 | 33644123 | G | T | INTRON | NA | 0.0773 |
| rs12186661 | 33644382 | G | A | INTRON | NA | 0.4820 |
| rs12186654 | 33644456 | C | A | INTRON | NA | 0.4595 |
| rs74378135 | 33644644 | A | G | INTRON | NA | 0.0076 |
| rs6887865 | 33644936 | C | T | INTRON | NA | 0.4597 |
| rs6887739 | 33644954 | A | G | INTRON | NA | 0.4553 |
| . | 33644996 | C | T | INTRON | NA |  |
| rs12153197 | 33645804 | T | A | INTRON | NA | 0.4575 |
| rs12152900 | 33645810 | C | T | INTRON | NA | 0.4575 |
| rs10072013 | 33645916 | A | C | INTRON | NA | 0.3702 |
| rs12187134 | 33646096 | G | T | INTRON | NA | 0.4593 |
| rs10065197 | 33646100 | T | A | INTRON | NA | 0.1442 |
| rs4129123 | 33646283 | T | C | INTRON | NA | 0.4595 |
| rs4129122 | 33646300 | C | T | INTRON | NA | 0.4595 |
| rs10057508 | 33646456 | G | C | INTRON | NA | 0.0845 |
| rs138614942 | 33646592 | T | G | INTRON | NA | 0.0016 |
| . | 33646616 | T | C | INTRON | NA |  |
| rs149284810 | 33646661 | A | G | INTRON | NA | 0.0074 |
| rs6451006 | 33646823 | A | G | INTRON | NA |  |
| rs4242081 | 33647057 | C | A | INTRON | NA | 0.4595 |
| rs4866412 | 33647284 | G | A | INTRON | NA | 0.4553 |
| rs10059623 | 33647440 | G | A | INTRON | NA | 0.4597 |
| rs13176485 | 33647634 | G | A | INTRON | NA | 0.0775 |
| rs189062459 | 33647741 | T | C | INTRON | NA | 0.0016 |
| rs10059808 | 33647744 | G | A | INTRON | NA | 0.4820 |
| rs4635933 | 33648062 | G | A | INTRON | NA | 0.4597 |
| rs4568371 | 33648267 | G | C | INTRON | NA | 0.1492 |
| rs4337860 | 33648271 | A | G | INTRON | NA | 0.4595 |
| rs10042292 | 33648317 | C | T | INTRON | NA | 0.4595 |
| rs10045283 | 33648501 | T | A | INTRON | NA | 0.4595 |
| rs11949958 | 33648707 | A | G | INTRON | NA |  |
| rs72741407 | 33648789 | C | G | INTRON | NA | 0.0030 |
| rs6870538 | 33648906 | G | C | INTRON | NA | 0.4597 |
| rs4866353 | 33649220 | A | G | INTRON | NA | 0.4595 |
| rs4242082 | 33649637 | G | A | INTRON | NA | 0.4597 |
| rs4504387 | 33649807 | C | A | INTRON | NA | 0.4597 |
| rs4621563 | 33649905 | G | A | INTRON | NA | 0.4595 |
| rs59536722 | 33650304 | G | A | INTRON | NA | 0.0503 |
| rs78638486 | 33650354 | G | T | INTRON | NA | 0.0022 |
| rs4340923 | 33650523 | C | A | INTRON | NA | 0.1953 |
| rs75294462 | 33650971 | A | G | INTRON | NA | 0.0054 |
| rs113158485 | 33651001 | C | T | INTRON | NA | 0.0060 |
| rs34076126 | 33652049 | T | C | INTRON | NA | 0.0144 |
| rs13186176 | 33652670 | C | A | INTRON | NA | 0.0777 |
| rs13186184 | 33652675 | C | A | INTRON | NA | 0.0777 |
| rs146500273 | 33653483 | G | C | INTRON | NA | 0.0032 |
| rs183979631 | 33654057 | T | C | INTRON | NA | 0.0008 |
| rs10052473 | 33654160 | C | T | INTRON | NA | 0.4820 |
| rs13159053 | 33654560 | G | A | INTRON | NA | 0.0853 |
| rs79641002 | 33654854 | T | C | INTRON | NA | 0.0074 |
| rs75811467 | 33654859 | T | C | INTRON | NA | 0.0008 |
| rs4866357 | 33655501 | C | T | INTRON | NA | 0.0425 |
| rs10472230 | 33656085 | G | C | INTRON | NA | 0.1360 |
| . | 33656167 | T | G | INTRON | NA |  |
| . | 33656552 | C | A | INTRON | NA |  |
| rs76488179 | 33657555 | A | G | INTRON | NA | 0.0250 |
| rs180724470 | 33659817 | C | T | INTRON | NA | 0.0002 |
| rs6862337 | 33659825 | A | T | INTRON | NA | 0.4780 |
| rs190024025 | 33660293 | T | C | INTRON | NA | 0.0004 |
| rs34234503 | 33660468 | C | T | INTRON | NA | 0.1428 |
| rs78885641 | 33660551 | T | C | INTRON | NA | 0.0146 |
| . | 33660896 | A | T | INTRON | NA |  |
| rs6451007 | 33661909 | A | G | INTRON | NA | 0.0230 |
| rs75873234 | 33662914 | C | T | INTRON | NA | 0.0108 |
| rs12516835 | 33663016 | C | A | INTRON | NA | 0.4641 |
| rs4544826 | 33663770 | A | T | INTRON | NA | 0.4371 |
| rs7735589 | 33664657 | A | T | INTRON | NA | 0.1556 |
| rs4866432 | 33664840 | T | C | INTRON | NA | 0.3876 |
| rs6861490 | 33665290 | C | G | INTRON | NA | 0.4756 |
| . | 33666069 | C | T | INTRON | NA |  |
| rs115319327 | 33666332 | C | A | INTRON | NA | 0.0104 |
| rs10472873 | 33666554 | G | A | INTRON | NA | 0.3387 |
| rs10472874 | 33666723 | T | G | INTRON | NA | 0.3395 |
| rs10472875 | 33666745 | G | T | INTRON | NA | 0.3397 |
| rs10472876 | 33666756 | A | C | INTRON | NA | 0.3397 |
| rs10472877 | 33666804 | G | T | INTRON | NA | 0.3393 |
| rs10472878 | 33666848 | A | G | INTRON | NA | 0.3393 |
| rs10472879 | 33666892 | G | A | INTRON | NA | 0.3401 |
| rs34141937 | 33666970 | G | A | INTRON | NA | 0.3419 |
| rs4866358 | 33667277 | G | C | INTRON | NA | 0.0803 |
| rs4866359 | 33667298 | T | C | INTRON | NA | 0.0719 |
| rs4242085 | 33667386 | G | A | INTRON | NA | 0.1689 |
| rs4242086 | 33667409 | T | G | INTRON | NA | 0.1567 |
| rs12188318 | 33667602 | C | T | INTRON | NA | 0.0258 |
| rs12189209 | 33667640 | T | G | INTRON | NA | 0.2957 |
| rs7714190 | 33667644 | G | A | INTRON | NA | 0.0533 |
| rs4343844 | 33667722 | A | G | INTRON | NA | 0.3758 |
| rs4640797 | 33667730 | T | C | INTRON | NA | 0.3834 |
| rs9292503 | 33667753 | G | T | INTRON | NA | 0.3425 |
| rs4321753 | 33667805 | A | C | INTRON | NA | 0.2069 |
| . | 33667880 | G | A | INTRON | NA |  |
| rs4476726 | 33668070 | A | G | INTRON | NA |  |
| rs77920273 | 33668184 | C | T | INTRON | NA | 0.0439 |
| rs4524517 | 33668280 | G | A | INTRON | NA | 0.0342 |
| rs7718859 | 33668542 | A | C | INTRON | NA | 0.0527 |
| rs7718998 | 33668548 | C | T | INTRON | NA | 0.0527 |
| rs59579183 | 33668657 | G | C | INTRON | NA | 0.1801 |
| rs7719028 | 33668670 | A | G | INTRON | NA | 0.0527 |
| rs7719460 | 33668691 | G | A | INTRON | NA | 0.0527 |
| rs12187406 | 33668768 | C | T | INTRON | NA | 0.0527 |
| rs12187423 | 33668852 | G | T | INTRON | NA | 0.0527 |
| rs13181942 | 33669014 | G | C | INTRON | NA | 0.1028 |
| rs13163819 | 33669050 | T | A | INTRON | NA | 0.0527 |
| rs10461705 | 33669127 | T | C | INTRON | NA | 0.0527 |
| rs10461706 | 33669397 | C | T | INTRON | NA | 0.0509 |
| rs149689672 | 33669473 | G | C | INTRON | NA | 0.0054 |
| rs10461926 | 33669637 | C | T | INTRON | NA | 0.0541 |
| rs11956568 | 33669808 | T | C | INTRON | NA | 0.0188 |
| rs12697294 | 33669973 | G | C | INTRON | NA | 0.0541 |
| rs10941078 | 33670370 | A | G | INTRON | NA | 0.0547 |
| rs10941079 | 33670487 | C | T | INTRON | NA | 0.0543 |
| rs12187825 | 33670572 | C | T | INTRON | NA | 0.0545 |
| rs12189429 | 33670660 | A | T | INTRON | NA |  |
| rs12189439 | 33670779 | A | G | INTRON | NA | 0.0543 |
| rs12187852 | 33670788 | G | A | INTRON | NA | 0.0543 |
| rs4621564 | 33670937 | A | G | INTRON | NA | 0.0543 |
| rs4593272 | 33670975 | A | G | INTRON | NA | 0.0186 |
| rs4643962 | 33671029 | T | C | INTRON | NA | 0.0543 |
| rs62349646 | 33671112 | A | G | INTRON | NA | 0.1416 |
| rs4352600 | 33671338 | G | T | INTRON | NA | 0.0543 |
| rs62349647 | 33671898 | T | C | INTRON | NA | 0.0198 |
| rs192367383 | 33672747 | A | G | INTRON | NA | 0.0054 |
| rs10070624 | 33673381 | G | A | INTRON | NA | 0.0543 |
| rs10070578 | 33673414 | C | T | INTRON | NA | 0.0543 |
| rs12653176 | 33673505 | A | T | INTRON | NA | 0.3169 |
| rs10070635 | 33673510 | C | T | INTRON | NA | 0.1048 |
| rs77682564 | 33673594 | C | T | INTRON | NA | 0.0505 |
| rs12657364 | 33673805 | C | T | INTRON | NA | 0.0557 |
| rs76223015 | 33673885 | C | G | INTRON | NA | 0.0505 |
| . | 33673894 | A | C | INTRON | NA |  |
| rs12657847 | 33673954 | G | A | INTRON | NA | 0.0513 |
| rs34017745 | 33673964 | C | T | INTRON | NA | 0.1969 |
| rs12660055 | 33674014 | T | A | INTRON | NA | 0.0513 |
| rs12657882 | 33674101 | G | T | INTRON | NA | 0.0513 |
| rs12657884 | 33674149 | G | C | INTRON | NA |  |
| rs79369771 | 33674432 | A | T | INTRON | NA | 0.0509 |
| rs6895282 | 33674441 | C | T | INTRON | NA | 0.0709 |
| rs6451012 | 33674719 | A | G | INTRON | NA | 0.0186 |
| . | 33674907 | A | G | INTRON | NA |  |
| rs149125970 | 33675216 | G | A | INTRON | NA | 0.0008 |
| rs13158685 | 33675395 | T | C | INTRON | NA | 0.1635 |
| rs10052864 | 33675754 | T | C | INTRON | NA | 0.0871 |
| rs6865556 | 33676043 | C | T | INTRON | NA | 0.0871 |
| rs11749450 | 33676396 | C | A | INTRON | NA | 0.1156 |
| rs138102655 | 33676661 | T | C | INTRON | NA | 0.0100 |
| rs6893612 | 33677527 | T | C | INTRON | NA | 0.0477 |
| rs149236799 | 33677632 | C | T | INTRON | NA | 0.0010 |
| rs181501000 | 33677748 | A | G | INTRON | NA | 0.0012 |
| rs4437379 | 33678161 | C | A | INTRON | NA | 0.3984 |
| rs4437380 | 33678250 | C | T | INTRON | NA | 0.3810 |
| rs4340924 | 33678353 | T | C | INTRON | NA | 0.3165 |
| rs28645462 | 33678515 | G | T | INTRON | NA |  |
| rs28654376 | 33678973 | G | C | INTRON | NA | 0.0919 |
| rs4272135 | 33679189 | A | G | INTRON | NA | 0.0966 |
| rs4454056 | 33679243 | T | C | INTRON | NA | 0.1020 |
| rs4527609 | 33679245 | G | A | INTRON | NA | 0.1016 |
| rs72741419 | 33679270 | A | G | INTRON | NA | 0.1957 |
| rs10072189 | 33679766 | C | T | INTRON | NA | 0.0963 |
| rs10072340 | 33679795 | G | T | INTRON | NA | 0.0955 |
| rs10057193 | 33679849 | A | T | INTRON | NA | 0.0046 |
| rs72741420 | 33679963 | T | C | INTRON | NA | 0.1935 |
| rs10073899 | 33679984 | G | T | INTRON | NA | 0.0963 |
| rs10051817 | 33679999 | T | C | INTRON | NA | 0.0963 |
| rs10073852 | 33680014 | C | T | INTRON | NA | 0.0963 |
| rs147872298 | 33680341 | T | A | INTRON | NA | 0.0002 |
| rs35953152 | 33680348 | G | A | INTRON | NA | 0.4645 |
| rs4866360 | 33680442 | C | T | INTRON | NA | 0.4343 |
| rs62349657 | 33680443 | G | T | INTRON | NA | 0.0963 |
| rs62349658 | 33680457 | A | G | INTRON | NA | 0.0963 |
| . | 33680481 | G | A | INTRON | NA |  |
| rs28694452 | 33681175 | A | G | INTRON | NA | 0.0963 |
| rs28734991 | 33681281 | C | T | INTRON | NA | 0.0966 |
| rs28405871 | 33681317 | G | C | INTRON | NA | 0.0889 |
| rs10076297 | 33681642 | C | T | INTRON | NA | 0.3055 |
| rs10076385 | 33681786 | C | T | INTRON | NA | 0.0971 |
| rs116529456 | 33681928 | A | T | INTRON | NA | 0.0002 |
| rs78116490 | 33682029 | A | T | INTRON | NA | 0.0056 |
| rs10063113 | 33682032 | A | G | INTRON | NA | 0.0963 |
| rs10063126 | 33682089 | A | T | INTRON | NA | 0.0961 |
| rs10056057 | 33682143 | T | G | INTRON | NA | 0.0963 |
| rs62352060 | 33682246 | C | T | INTRON | NA | 0.2057 |
| rs57207780 | 33682305 | A | G | INTRON | NA | 0.2169 |
| rs62352061 | 33682496 | C | A | INTRON | NA | 0.0959 |
| rs62352062 | 33682727 | G | A | INTRON | NA |  |
| rs62352063 | 33682757 | G | A | INTRON | NA | 0.0961 |
| rs62352064 | 33682860 | A | G | INTRON | NA | 0.0961 |
| rs80018223 | 33682864 | C | T | INTRON | NA | 0.2085 |
| rs62352065 | 33683028 | G | C | INTRON | NA |  |
| rs10080136 | 33683089 | C | T | INTRON | NA | 0.0959 |
| rs6451013 | 33683551 | C | T | INTRON | NA | 0.0046 |
| rs12651808 | 33683696 | G | A | INTRON | NA | 0.2083 |
| rs72741422 | 33684287 | G | A | INTRON | NA | 0.1923 |
| rs62352066 | 33684293 | A | G | INTRON | NA | 0.0957 |
| rs185074320 | 33684423 | G | A | INTRON | NA | 0.0002 |
| rs6881709 | 33684426 | C | A | INTRON | NA | 0.0046 |
| rs76389904 | 33684538 | A | G | INTRON | NA | 0.2165 |
| rs4406136 | 33684699 | C | T | INTRON | NA | 0.0046 |
| rs114570109 | 33684777 | T | C | INTRON | NA | 0.0042 |
| rs72741423 | 33685010 | T | G | INTRON | NA | 0.1923 |
| rs12652847 | 33685042 | C | A | INTRON | NA | 0.0949 |
| rs75766198 | 33685321 | T | C | INTRON | NA | 0.2153 |
| rs11739910 | 33685359 | G | C | INTRON | NA | 0.1879 |
| rs10062990 | 33685863 | T | C | INTRON | NA | 0.4461 |
| rs116080982 | 33685924 | A | G | INTRON | NA | 0.0162 |
| rs4558994 | 33686080 | C | T | INTRON | NA | 0.4890 |
| rs4360047 | 33686344 | C | T | INTRON | NA | 0.2135 |
| rs7732212 | 33686480 | C | T | INTRON | NA | 0.4864 |
| rs115169076 | 33686668 | G | C | INTRON | NA | 0.0070 |
| rs13169784 | 33686762 | C | G | INTRON | NA | 0.4722 |
| rs9784619 | 33686861 | C | T | INTRON | NA | 0.4714 |
| rs13169825 | 33686872 | A | C | INTRON | NA | 0.1356 |
| rs59134524 | 33686874 | A | C | INTRON | NA | 0.2214 |
| rs116224810 | 33686904 | T | G | INTRON | NA | 0.0308 |
| rs78609389 | 33686906 | G | T | INTRON | NA | 0.2212 |
| rs9784714 | 33686971 | A | C | INTRON | NA | 0.1715 |
| rs10941080 | 33687535 | A | G | INTRON | NA | 0.4691 |
| rs6899224 | 33687594 | G | A | INTRON | NA | 0.2518 |
| rs7719969 | 33687640 | T | C | INTRON | NA | 0.4421 |
| rs6859145 | 33687779 | G | A | INTRON | NA | 0.2726 |
| rs6451014 | 33688036 | T | C | INTRON | NA | 0.0040 |
| rs10057610 | 33688094 | T | C | INTRON | NA | 0.2518 |
| rs9292504 | 33688167 | A | C | INTRON | NA | 0.2518 |
| rs4260679 | 33688538 | C | G | INTRON | NA | 0.0044 |
| rs10472882 | 33688697 | G | T | INTRON | NA | 0.2518 |
| rs10472883 | 33688835 | A | G | INTRON | NA | 0.2518 |
| . | 33688858 | C | T | INTRON | NA |  |
| rs10472884 | 33688870 | T | C | INTRON | NA | 0.2518 |
| rs67600960 | 33689032 | C | T | INTRON | NA | 0.2518 |
| rs12652117 | 33689392 | C | T | INTRON | NA | 0.2518 |
| . | 33689580 | G | C | INTRON | NA |  |
| rs78643504 | 33689593 | C | T | INTRON | NA | 0.2093 |
| rs10052224 | 33689624 | G | A | INTRON | NA | 0.2520 |
| rs4866361 | 33690093 | C | G | INTRON | NA | 0.2518 |
| rs4866362 | 33690120 | G | A | INTRON | NA | 0.2518 |
| rs4866363 | 33690313 | T | G | INTRON | NA | 0.2522 |
| rs10472885 | 33690435 | T | C | INTRON | NA | 0.0885 |
| rs9292505 | 33690780 | C | T | INTRON | NA | 0.0885 |
| rs4866364 | 33690860 | C | T | INTRON | NA | 0.0050 |
| . | 33690867 | T | C | INTRON | NA |  |
| rs10055979 | 33691164 | C | T | INTRON | NA | 0.0883 |
| rs4308487 | 33691330 | A | C | INTRON | NA | 0.1997 |
| rs10064197 | 33691438 | T | G | INTRON | NA | 0.2512 |
| . | 33691693 | C | T | INTRON | NA |  |
| rs10056476 | 33691751 | G | A | INTRON | NA | 0.2512 |
| rs10472887 | 33692260 | T | C | INTRON | NA | 0.2516 |
| rs10472888 | 33692396 | A | G | INTRON | NA | 0.2514 |
| rs10472889 | 33692591 | C | T | INTRON | NA | 0.2516 |
| rs9292507 | 33692705 | C | T | INTRON | NA | 0.2516 |
| rs28409078 | 33692805 | A | T | INTRON | NA | 0.1631 |
| rs74771932 | 33692819 | G | A | INTRON | NA | 0.2258 |
| rs10075107 | 33692996 | A | G | INTRON | NA | 0.2514 |
| rs10068461 | 33693368 | T | C | INTRON | NA | 0.2514 |
| rs10060487 | 33693452 | C | T | INTRON | NA | 0.0883 |
| rs10060550 | 33693539 | C | T | INTRON | NA | 0.2516 |
| rs10075428 | 33693584 | A | G | INTRON | NA | 0.2514 |
| rs13359675 | 33693946 | T | A | INTRON | NA | 0.1631 |
| rs80291708 | 33694073 | A | G | INTRON | NA | 0.2722 |
| rs114488172 | 33694110 | C | T | INTRON | NA |  |
| rs10062422 | 33694175 | C | T | INTRON | NA | 0.2514 |
| rs6867843 | 33694265 | C | A | INTRON | NA | 0.2151 |
| rs4866365 | 33694366 | T | C | INTRON | NA | 0.2514 |
| . | 33694426 | T | C | INTRON | NA |  |
| rs2032877 | 33694998 | C | T | INTRON | NA | 0.2089 |
| rs10064467 | 33695026 | C | T | INTRON | NA | 0.0883 |
| rs10064522 | 33695182 | C | A | INTRON | NA | 0.0883 |
| . | 33695224 | T | G | INTRON | NA |  |
| rs10064603 | 33695264 | C | T | INTRON | NA | 0.2510 |
| rs116586798 | 33695490 | T | C | INTRON | NA | 0.0054 |
| rs4866369 | 33695585 | G | A | INTRON | NA | 0.1625 |
| rs4866370 | 33695590 | T | C | INTRON | NA | 0.2504 |
| rs72741435 | 33695738 | C | A | INTRON | NA | 0.0134 |
| rs79896697 | 33695842 | C | T | INTRON | NA | 0.0036 |
| rs57789202 | 33695844 | G | A | INTRON | NA | 0.0877 |
| rs4866371 | 33695884 | G | C | INTRON | NA | 0.2530 |
| rs4866372 | 33695944 | A | T | INTRON | NA | 0.1653 |
| rs4866373 | 33695969 | T | C | INTRON | NA | 0.2532 |
| . | 33696215 | T | A | INTRON | NA |  |
| rs4583889 | 33696293 | A | G | INTRON | NA | 0.0054 |
| rs13358697 | 33696442 | C | T | INTRON | NA | 0.0875 |
| rs6860713 | 33696550 | T | G | INTRON | NA | 0.2630 |
| rs4461641 | 33696645 | G | C | INTRON | NA | 0.2634 |
| rs140225475 | 33696792 | T | C | INTRON | NA | 0.0024 |
| rs35185995 | 33696855 | A | G | INTRON | NA | 0.2119 |
| rs62352072 | 33697061 | T | A | INTRON | NA | 0.0875 |
| rs6451015 | 33697285 | T | A | INTRON | NA | 0.2634 |
| rs113142828 | 33697877 | G | T | INTRON | NA | 0.0214 |
| rs7729066 | 33697898 | A | G | INTRON | NA | 0.1701 |
| rs7729067 | 33697899 | A | C | INTRON | NA | 0.2592 |
| rs34001351 | 33698019 | G | A | INTRON | NA | 0.2115 |
| rs35443495 | 33698928 | C | T | INTRON | NA | 0.2087 |
| . | 33698988 | G | A | INTRON | NA |  |
| rs7448880 | 33699106 | A | G | INTRON | NA | 0.1623 |
| rs4461640 | 33699374 | T | C | INTRON | NA | 0.0357 |
| rs2113011 | 33699551 | C | A | INTRON | NA |  |
| rs4866374 | 33699618 | C | T | INTRON | NA | 0.1777 |
| rs966857 | 33699676 | C | T | INTRON | NA | 0.2250 |
| rs966855 | 33699777 | G | A | INTRON | NA | 0.2151 |
| rs10039504 | 33699808 | G | T | INTRON | NA | 0.0855 |
| rs6451016 | 33699859 | C | G | INTRON | NA |  |
| rs966854 | 33699969 | A | G | INTRON | NA | 0.2244 |
| rs10045599 | 33700094 | A | G | INTRON | NA | 0.0863 |
| rs7722626 | 33700229 | T | C | INTRON | NA | 0.2606 |
| rs4866375 | 33700650 | G | C | INTRON | NA | 0.1683 |
| rs80071375 | 33700761 | C | A | INTRON | NA |  |
| rs35290190 | 33700848 | G | T | INTRON | NA | 0.2228 |
| rs62352075 | 33700891 | C | T | INTRON | NA | 0.0132 |
| rs2331243 | 33701626 | T | C | INTRON | NA | 0.2177 |
| rs113245301 | 33701649 | G | C | INTRON | NA | 0.0052 |
| . | 33701791 | G | A | INTRON | NA |  |
| rs4295395 | 33701888 | A | G | INTRON | NA | 0.0044 |
| rs34072171 | 33702148 | A | G | INTRON | NA | 0.1823 |
| . | 33702697 | A | T | INTRON | NA |  |
| rs7700370 | 33702830 | T | C | INTRON | NA | 0.2456 |
| rs6451018 | 33703049 | A | C | INTRON | NA | 0.2226 |
| rs1119629 | 33703734 | T | C | INTRON | NA | 0.1849 |
| rs76685589 | 33704400 | G | A | INTRON | NA | 0.2177 |
| rs78662291 | 33704541 | G | A | INTRON | NA | 0.0026 |
| rs7734575 | 33704608 | G | A | INTRON | NA | 0.4619 |
| rs1946263 | 33704707 | A | T | INTRON | NA | 0.2226 |
| rs7734388 | 33704785 | A | T | INTRON | NA | 0.4617 |
| rs1946261 | 33705045 | T | C | INTRON | NA | 0.2220 |
| rs11958410 | 33705142 | C | G | INTRON | NA | 0.4595 |
| rs56022747 | 33705259 | G | A | INTRON | NA | 0.3728 |
| rs6860522 | 33705654 | A | C | INTRON | NA | 0.4593 |
| rs6860816 | 33705743 | C | T | INTRON | NA | 0.4591 |
| rs6861143 | 33705795 | G | A | INTRON | NA | 0.4591 |
| rs6451019 | 33706075 | C | T | INTRON | NA | 0.4591 |
| rs7706826 | 33706513 | A | G | INTRON | NA | 0.4591 |
| rs35452381 | 33706708 | T | C | INTRON | NA | 0.0633 |
| rs11749547 | 33706751 | G | A | INTRON | NA | 0.4591 |
| rs142888151 | 33708320 | T | A | INTRON | NA |  |
| rs114449463 | 33708391 | G | C | INTRON | NA |  |
| rs12520773 | 33708957 | G | A | INTRON | NA | 0.4591 |
| rs12515646 | 33709296 | A | G | INTRON | NA | 0.4591 |
| rs7722460 | 33709359 | A | G | INTRON | NA | 0.0044 |
| rs12523495 | 33709439 | T | C | INTRON | NA | 0.4597 |
| rs12520852 | 33709518 | C | T | INTRON | NA | 0.4595 |
| rs140640237 | 33709666 | T | G | INTRON | NA | 0.0569 |
| rs13190046 | 33709700 | T | C | INTRON | NA |  |
| rs13169978 | 33709705 | C | G | INTRON | NA | 0.3672 |
| rs7705604 | 33709761 | T | G | INTRON | NA | 0.2220 |
| . | 33709859 | G | A | INTRON | NA |  |
| rs7727220 | 33709863 | C | G | INTRON | NA | 0.2220 |
| rs116389950 | 33710082 | G | A | INTRON | NA |  |
| rs144428593 | 33710321 | A | G | INTRON | NA | 0.0016 |
| rs34893763 | 33710632 | T | A | INTRON | NA | 0.3666 |
| rs74486111 | 33710791 | C | G | INTRON | NA | 0.0110 |
| rs34159774 | 33710848 | A | T | INTRON | NA | 0.4589 |
| rs78558389 | 33711035 | G | C | INTRON | NA | 0.0633 |
| . | 33711250 | G | A | INTRON | NA |  |
| rs10054073 | 33711396 | T | G | INTRON | NA | 0.2212 |
| rs10076096 | 33711415 | C | A | INTRON | NA | 0.3187 |
| rs11749202 | 33711615 | C | A | INTRON | NA | 0.2196 |
| rs11739711 | 33711766 | T | G | INTRON | NA | 0.4597 |
| rs1423489 | 33711988 | C | T | INTRON | NA | 0.3115 |
| rs10078039 | 33712059 | G | T | INTRON | NA | 0.1695 |
| rs12522913 | 33712279 | C | T | INTRON | NA | 0.4621 |
| rs12522957 | 33712442 | C | T | INTRON | NA | 0.4593 |
| rs13362069 | 33712526 | G | T | INTRON | NA | 0.3109 |
| rs10050510 | 33713561 | C | T | INTRON | NA | 0.1695 |
| rs4400128 | 33713661 | T | C | INTRON | NA | 0.4599 |
| rs9292509 | 33713807 | C | T | INTRON | NA | 0.4762 |
| rs12516802 | 33713958 | T | G | INTRON | NA | 0.4599 |
| rs12519205 | 33713963 | A | G | INTRON | NA | 0.4599 |
| . | 33714206 | G | A | INTRON | NA |  |
| rs11738892 | 33714304 | C | T | INTRON | NA | 0.4591 |
| rs7721240 | 33714550 | A | C | INTRON | NA | 0.2216 |
| rs12516961 | 33714657 | T | C | INTRON | NA | 0.1815 |
| rs7721562 | 33714710 | A | G | INTRON | NA | 0.4597 |
| rs7725945 | 33714891 | G | A | INTRON | NA | 0.4591 |
| rs7708254 | 33715042 | T | C | INTRON | NA | 0.4423 |
| rs34517117 | 33715308 | T | C | INTRON | NA | 0.1394 |
| rs1895440 | 33715457 | C | T | INTRON | NA | 0.4706 |
| rs11740622 | 33715979 | C | A | INTRON | NA | 0.1392 |
| rs77672241 | 33716621 | G | T | INTRON | NA | 0.0925 |
| . | 33717344 | T | G | INTRON | NA |  |
| rs7737540 | 33717599 | C | T | INTRON | NA | 0.4882 |
| rs4409096 | 33717880 | T | C | INTRON | NA | 0.4046 |
| rs141643330 | 33718032 | C | T | INTRON | NA | 0.0020 |
| rs140139312 | 33718046 | A | G | INTRON | NA | 0.0038 |
| rs2877318 | 33718160 | T | G | INTRON | NA | 0.4946 |
| rs181743713 | 33718461 | C | T | INTRON | NA | 0.0014 |
| rs12519012 | 33719032 | A | G | INTRON | NA | 0.4952 |
| rs6451020 | 33719436 | A | G | INTRON | NA | 0.4157 |
| rs115610684 | 33719715 | C | T | INTRON | NA | 0.0170 |
| rs1423476 | 33720185 | C | A | INTRON | NA | 0.4812 |
| rs1423477 | 33720267 | C | T | INTRON | NA | 0.4938 |
| rs4866377 | 33720776 | G | A | INTRON | NA | 0.4954 |
| rs4866378 | 33720807 | A | G | INTRON | NA | 0.4818 |
| rs4866379 | 33720813 | T | C | INTRON | NA | 0.4129 |
| rs7720544 | 33721347 | A | G | INTRON | NA | 0.4816 |
| rs76199296 | 33721382 | C | T | INTRON | NA | 0.0659 |
| rs4866380 | 33721539 | G | A | INTRON | NA | 0.4708 |
| rs138533609 | 33721597 | C | T | INTRON | NA | 0.0016 |
| rs4866381 | 33721765 | G | A | INTRON | NA | 0.4046 |
| rs75591639 | 33722088 | G | A | INTRON | NA | 0.0637 |
| rs75837826 | 33722190 | G | A | INTRON | NA | 0.0661 |
| rs79798404 | 33722255 | C | T | INTRON | NA | 0.0663 |
| . | 33723221 | T | C | INTRON | NA |  |
| . | 33723588 | C | G | INTRON | NA |  |
| rs1423478 | 33723821 | C | A | INTRON | NA | 0.3660 |
| rs1423479 | 33723884 | A | G | INTRON | NA | 0.1907 |
| rs1423480 | 33723895 | G | A | INTRON | NA | 0.3654 |
| rs150984085 | 33724189 | T | C | INTRON | NA | 0.0026 |
| rs115952475 | 33724630 | G | T | INTRON | NA | 0.0088 |
| rs58731794 | 33725064 | A | T | INTRON | NA | 0.0957 |
| rs12659781 | 33725145 | A | G | INTRON | NA | 0.0683 |
| rs113610704 | 33725495 | C | A | INTRON | NA | 0.0056 |
| rs4377712 | 33725681 | A | G | INTRON | NA | 0.4457 |
| rs10066561 | 33725956 | C | G | INTRON | NA | 0.2702 |
| rs7718949 | 33726336 | A | G | INTRON | NA | 0.2125 |
| rs4866342 | 33727008 | G | A | INTRON | NA | 0.0671 |
| rs143410709 | 33727416 | G | A | INTRON | NA | 0.0012 |
| . | 33727909 | T | C | INTRON | NA |  |
| rs34526999 | 33728540 | A | G | INTRON | NA | 0.4267 |
| rs141826760 | 33729491 | T | C | INTRON | NA | 0.0004 |
| rs116411032 | 33729671 | A | G | INTRON | NA | 0.0020 |
| rs55817365 | 33730036 | C | T | INTRON | NA |  |
| rs11948306 | 33731503 | A | G | INTRON | NA | 0.3538 |
| rs115060689 | 33733758 | G | A | INTRON | NA | 0.0128 |
| rs141078298 | 33734388 | T | C | INTRON | NA | 0.0020 |
| rs115477131 | 33734747 | G | A | INTRON | NA | 0.0044 |
| rs4866344 | 33736313 | G | A | INTRON | NA | 0.3427 |
| rs188592667 | 33736387 | C | T | INTRON | NA | 0.0002 |
| rs10941084 | 33736909 | G | A | INTRON | NA | 0.3427 |
| rs4866382 | 33737332 | T | C | INTRON | NA | 0.0671 |
| rs62349767 | 33737833 | A | G | INTRON | NA | 0.0036 |
| rs9784618 | 33739306 | A | G | INTRON | NA | 0.2416 |
| . | 33739342 | G | A | INTRON | NA |  |
| . | 33739780 | T | A | INTRON | NA |  |
| rs28585053 | 33740993 | C | T | INTRON | NA | 0.4087 |
| . | 33741295 | T | C | INTRON | NA |  |
| rs187914129 | 33741422 | G | C | INTRON | NA | 0.0002 |
| rs2331304 | 33741991 | G | A | INTRON | NA | 0.3708 |
| rs1862590 | 33742545 | T | C | INTRON | NA | 0.2051 |
| rs1862591 | 33742571 | T | A | INTRON | NA | 0.2179 |
| rs149129120 | 33742993 | G | A | INTRON | NA | 0.0010 |
| rs189390570 | 33743466 | C | G | INTRON | NA | 0.0020 |
| rs10941085 | 33744046 | T | A | INTRON | NA | 0.4141 |
| rs4866383 | 33744247 | G | A | INTRON | NA | 0.4451 |
| rs4866384 | 33744532 | T | C | INTRON | NA | 0.3650 |
| rs7703841 | 33744740 | T | A | INTRON | NA | 0.4359 |
| rs7703842 | 33744743 | T | C | INTRON | NA | 0.3648 |
| rs11745480 | 33744830 | A | G | INTRON | NA | 0.4361 |
| rs10052828 | 33744893 | G | T | INTRON | NA | 0.2105 |
| rs11739718 | 33745016 | C | T | INTRON | NA | 0.4361 |
| rs3966166 | 33745095 | A | T | INTRON | NA | 0.4361 |
| rs1968600 | 33745568 | T | A | INTRON | NA | 0.3648 |
| rs1966796 | 33745589 | T | C | INTRON | NA | 0.2173 |
| rs13164467 | 33745882 | G | C | INTRON | NA | 0.0290 |
| rs149781477 | 33746001 | C | T | INTRON | NA | 0.0016 |
| rs1982326 | 33746017 | C | G | INTRON | NA | 0.4227 |
| . | 33746258 | A | G | INTRON | NA |  |
| rs11744137 | 33746339 | T | C | INTRON | NA | 0.0094 |
| rs148280812 | 33746883 | A | G | INTRON | NA | 0.0054 |
| rs187682164 | 33747398 | G | A | INTRON | NA | 0.0006 |
| rs77929852 | 33747439 | T | C | INTRON | NA | 0.0038 |
| rs1423482 | 33747587 | G | A | INTRON | NA | 0.1368 |
| . | 33747599 | G | A | INTRON | NA |  |
| rs114157969 | 33748046 | G | A | INTRON | NA |  |
| rs1014525 | 33748542 | C | T | INTRON | NA | 0.3003 |
| rs1549258 | 33748600 | C | T | INTRON | NA | 0.3005 |
| rs6891022 | 33749568 | T | C | INTRON | NA | 0.3696 |
| rs6895637 | 33749812 | T | G | INTRON | NA | 0.3496 |
| rs6874519 | 33750121 | A | C | INTRON | NA |  |
| rs1423481 | 33750353 | T | C | INTRON | NA | 0.3496 |
| rs62349783 | 33750798 | C | T | INTRON | NA | 0.0038 |
| rs78790832 | 33751203 | A | T | INTRON | NA | 0.1831 |
| rs115479662 | 33751332 | A | T | INTRON | NA | 0.0162 |
| rs1530507 | 33751454 | A | T | INTRON | NA | 0.4431 |
| rs1562245 | 33751919 | T | C | INTRON | NA | 0.3592 |
| . | 33752090 | C | T | INTRON | NA |  |
| rs35355167 | 33752178 | T | C | INTRON | NA | 0.0671 |
| rs2194240 | 33754138 | T | G | INTRON | NA | 0.3357 |
| rs62349784 | 33754322 | T | C | INTRON | NA | 0.1947 |
| rs734118 | 33754602 | C | T | INTRON | NA | 0.2550 |
| rs188604664 | 33754614 | T | C | INTRON | NA | 0.0018 |
| rs10941086 | 33754922 | T | C | INTRON | NA | 0.0793 |
| rs56196043 | 33755562 | G | T | INTRON | NA | 0.1935 |
| rs34072316 | 33755990 | A | T | INTRON | NA | 0.0793 |
| rs9292512 | 33756520 | A | G | INTRON | NA | 0.3596 |
| rs10472891 | 33756619 | T | G | INTRON | NA | 0.3257 |
| rs55979811 | 33756878 | G | A | INTRON | NA | 0.0575 |
| rs10472892 | 33756909 | C | T | INTRON | NA | 0.4433 |
| rs13172462 | 33757205 | A | G | INTRON | NA | 0.0793 |
| . | 33758016 | G | A | INTRON | NA |  |
| rs7712324 | 33758513 | T | C | INTRON | NA | 0.3363 |
| rs10521007 | 33758562 | G | A | INTRON | NA | 0.0791 |
| rs7734337 | 33758896 | C | T | INTRON | NA | 0.4461 |
| rs9790860 | 33759312 | G | A | INTRON | NA | 0.2554 |
| rs114455758 | 33759517 | T | A | INTRON | NA | 0.0162 |
| rs77676032 | 33759613 | T | G | INTRON | NA | 0.0064 |
| rs6896791 | 33759618 | C | T | INTRON | NA | 0.4389 |
| . | 33759677 | A | T | INTRON | NA |  |
| . | 33759770 | A | G | INTRON | NA |  |
| rs12054731 | 33760162 | G | A | INTRON | NA | 0.4387 |
| . | 33761094 | C | T | INTRON | NA |  |
| . | 33761126 | A | G | INTRON | NA |  |
| rs12109370 | 33761566 | T | C | INTRON | NA | 0.3293 |
| . | 33764138 | T | G | INTRON | NA |  |
| rs113922801 | 33764332 | A | G | INTRON | NA | 0.0046 |
| . | 33764448 | A | G | INTRON | NA |  |
| rs72741453 | 33765366 | C | T | INTRON | NA | 0.0254 |
| rs74861922 | 33765760 | G | A | INTRON | NA | 0.0040 |
| . | 33765931 | A | G | INTRON | NA |  |
| rs181442954 | 33766318 | C | G | INTRON | NA |  |
| rs186554184 | 33766459 | A | G | INTRON | NA | 0.0022 |
| rs113316840 | 33766901 | C | A | INTRON | NA | 0.0020 |
| rs16891679 | 33767310 | G | A | INTRON | NA | 0.1939 |
| rs10472893 | 33767646 | T | A | INTRON | NA | 0.3289 |
| rs188311109 | 33768875 | A | G | INTRON | NA | 0.0016 |
| rs115905374 | 33768935 | T | C | INTRON | NA |  |
| rs148349783 | 33769068 | C | T | INTRON | NA | 0.0070 |
| . | 33769372 | C | T | INTRON | NA |  |
| rs10061337 | 33769407 | C | T | INTRON | NA | 0.1933 |
| . | 33769907 | C | T | INTRON | NA |  |
| rs1423485 | 33770053 | C | T | INTRON | NA | 0.1933 |
| rs146053907 | 33770331 | G | A | INTRON | NA | 0.0030 |
| . | 33771967 | G | T | INTRON | NA |  |
| rs55659260 | 33772214 | G | A | INTRON | NA | 0.1877 |
| rs10472894 | 33772264 | G | C | INTRON | NA | 0.2206 |
| rs967409 | 33772391 | T | C | INTRON | NA | 0.1891 |
| rs889277 | 33772641 | T | G | INTRON | NA | 0.4321 |
| rs889276 | 33772696 | C | T | INTRON | NA | 0.4321 |
| . | 33772766 | T | C | INTRON | NA |  |
| . | 33772917 | G | A | INTRON | NA |  |
| rs58134922 | 33773223 | A | C | INTRON | NA | 0.2206 |
| rs7705691 | 33773262 | A | T | INTRON | NA | 0.4321 |
| rs10070109 | 33773500 | G | A | INTRON | NA | 0.1933 |
| rs182926206 | 33773783 | C | T | INTRON | NA | 0.0068 |
| rs9283730 | 33774275 | A | G | INTRON | NA | 0.2905 |
| rs4866385 | 33774414 | A | G | INTRON | NA | 0.3646 |
| rs189114305 | 33774732 | A | C | INTRON | NA | 0.0028 |
| rs181630156 | 33775619 | A | G | INTRON | NA | 0.0032 |
| rs9968578 | 33775653 | T | C | INTRON | NA | 0.2208 |
| rs67931447 | 33775783 | G | A | INTRON | NA | 0.0779 |
| rs112315344 | 33775793 | G | T | INTRON | NA | 0.0060 |
| rs182366131 | 33776371 | T | C | INTRON | NA | 0.0004 |
| rs7725466 | 33776531 | T | A | INTRON | NA | 0.4451 |
| rs11750049 | 33777125 | G | C | INTRON | NA | 0.0777 |
| rs55980087 | 33777811 | G | A | INTRON | NA | 0.2272 |
| . | 33778051 | T | C | INTRON | NA |  |
| . | 33778766 | T | C | INTRON | NA |  |
| rs74625313 | 33779359 | T | C | INTRON | NA | 0.4449 |
| rs113115866 | 33779417 | A | T | INTRON | NA | 0.2266 |
| rs62349788 | 33779577 | C | T | INTRON | NA | 0.1929 |
| rs62349789 | 33779608 | A | G | INTRON | NA | 0.1929 |
| . | 33780192 | A | C | INTRON | NA |  |
| rs74518462 | 33780416 | T | C | INTRON | NA | 0.0036 |
| rs72741455 | 33781288 | G | A | INTRON | NA | 0.0256 |
| rs13161116 | 33781687 | T | G | INTRON | NA | 0.4383 |
| rs141886422 | 33781753 | C | T | INTRON | NA | 0.0008 |
| rs114883961 | 33781798 | T | A | INTRON | NA | 0.0206 |
| rs56327650 | 33782050 | G | T | INTRON | NA | 0.2244 |
| rs142902726 | 33782215 | T | C | INTRON | NA | 0.0144 |
| rs115540568 | 33782355 | G | T | INTRON | NA | 0.0162 |
| rs9292513 | 33782820 | G | T | INTRON | NA | 0.1931 |
| . | 33783138 | T | C | INTRON | NA |  |
| rs10080046 | 33783791 | G | A | INTRON | NA | 0.4319 |
| rs7719861 | 33784613 | A | C | INTRON | NA | 0.4319 |
| rs10038678 | 33784636 | T | G | INTRON | NA | 0.1929 |
| . | 33784875 | T | C | INTRON | NA |  |
| rs66868236 | 33784881 | C | T | INTRON | NA | 0.0332 |
| rs10060162 | 33784886 | T | C | INTRON | NA | 0.2272 |
| rs55883332 | 33785556 | A | C | INTRON | NA | 0.1875 |
| rs10062147 | 33785624 | T | C | INTRON | NA | 0.2248 |
| . | 33785881 | C | T | INTRON | NA |  |
| rs28690244 | 33786271 | C | T | INTRON | NA | 0.2250 |
| rs182250768 | 33786339 | T | C | INTRON | NA | 0.0004 |
| rs1423488 | 33786877 | C | G | INTRON | NA | 0.4427 |
| rs10058403 | 33787621 | C | T | INTRON | NA | 0.2159 |
| rs76261075 | 33787879 | T | C | INTRON | NA | 0.0026 |
| rs112493515 | 33789210 | T | C | INTRON | NA | 0.0046 |
| rs61373561 | 33789475 | A | G | INTRON | NA | 0.0613 |
| rs16891722 | 33789693 | G | A | INTRON | NA | 0.1931 |
| rs137958892 | 33789957 | T | C | INTRON | NA | 0.0050 |
| rs34117085 | 33790216 | C | T | INTRON | NA | 0.0731 |
| rs10053594 | 33790733 | C | T | INTRON | NA | 0.2274 |
| rs190886813 | 33790907 | T | C | INTRON | NA | 0.0142 |
| rs9292514 | 33791095 | C | G | INTRON | NA | 0.4319 |
| rs67558897 | 33792089 | G | A | INTRON | NA | 0.0268 |
| rs1971180 | 33792172 | G | A | INTRON | NA | 0.1927 |
| rs7724739 | 33792456 | C | T | INTRON | NA | 0.4233 |
| rs187527568 | 33792803 | G | A | INTRON | NA | 0.0018 |
| . | 33793455 | A | G | INTRON | NA |  |
| rs6865823 | 33794113 | C | T | INTRON | NA | 0.1128 |
| rs10039443 | 33794797 | C | A | INTRON | NA | 0.1727 |
| rs188899629 | 33794949 | A | G | INTRON | NA | 0.0018 |
| rs9292515 | 33795031 | A | G | INTRON | NA | 0.1727 |
| rs6451022 | 33795056 | A | C | INTRON | NA | 0.4942 |
| rs7712316 | 33795167 | G | A | INTRON | NA | 0.1444 |
| rs13187377 | 33795421 | C | A | INTRON | NA | 0.1034 |
| rs1549259 | 33795799 | G | C | INTRON | NA | 0.3105 |
| rs1012814 | 33796235 | T | A | INTRON | NA | 0.3047 |
| rs7717852 | 33796518 | C | T | INTRON | NA | 0.3197 |
| rs5026245 | 33796710 | T | C | INTRON | NA | 0.2821 |
| rs13162619 | 33799480 | T | C | INTRON | NA | 0.0306 |
| rs2032878 | 33799699 | T | G | INTRON | NA | 0.1715 |
| rs1374023 | 33800634 | G | C | INTRON | NA |  |
| rs1374022 | 33800686 | T | C | INTRON | NA | 0.1713 |
| . | 33800764 | A | G | INTRON | NA |  |
| . | 33800933 | G | A | INTRON | NA |  |
| rs1374021 | 33800941 | A | C | INTRON | NA | 0.2730 |
| rs12658071 | 33801014 | T | G | INTRON | NA | 0.2356 |
| rs12523642 | 33801115 | A | G | INTRON | NA | 0.1344 |
| rs6866225 | 33801415 | G | A | INTRON | NA | 0.0841 |
| . | 33801620 | C | T | INTRON | NA |  |
| rs72741464 | 33801981 | C | A | INTRON | NA | 0.1156 |
| . | 33802234 | C | T | INTRON | NA |  |
| rs10941089 | 33802540 | T | A | INTRON | NA | 0.4952 |
| rs115010590 | 33802567 | A | C | INTRON | NA | 0.0084 |
| rs1900177 | 33802980 | C | G | INTRON | NA |  |
| rs1900176 | 33803105 | C | T | INTRON | NA | 0.2746 |
| . | 33803288 | G | A | INTRON | NA |  |
| . | 33803325 | C | T | INTRON | NA |  |
| rs2218523 | 33803454 | A | G | INTRON | NA | 0.2734 |
| . | 33803513 | A | G | INTRON | NA |  |
| rs2197517 | 33803716 | G | A | INTRON | NA | 0.2770 |
| rs28727882 | 33803809 | T | C | INTRON | NA | 0.1861 |
| rs28671325 | 33803978 | G | A | INTRON | NA | 0.1807 |
| . | 33803982 | T | A | INTRON | NA |  |
| rs4866386 | 33804416 | C | T | INTRON | NA |  |
| rs10941090 | 33804517 | A | G | INTRON | NA | 0.2318 |
| rs115500703 | 33804606 | C | T | INTRON | NA | 0.0044 |
| rs10472896 | 33804679 | C | T | INTRON | NA | 0.2314 |
| . | 33804701 | A | G | INTRON | NA |  |
| rs7737278 | 33805246 | C | A | INTRON | NA | 0.4127 |
| rs7737447 | 33805331 | C | T | INTRON | NA | 0.4058 |
| rs7447124 | 33806263 | T | C | INTRON | NA | 0.3395 |
| rs35418755 | 33807579 | C | T | INTRON | NA |  |
| rs9292516 | 33807907 | C | T | INTRON | NA | 0.3486 |
| . | 33807912 | A | T | INTRON | NA |  |
| . | 33807926 | G | C | INTRON | NA |  |
| . | 33808769 | G | T | INTRON | NA |  |
| rs1037104 | 33808956 | G | A | INTRON | NA | 0.4479 |
| rs10941091 | 33809047 | T | C | INTRON | NA | 0.1270 |
| rs1037102 | 33809303 | T | G | INTRON | NA | 0.4213 |
| rs13167500 | 33809330 | A | G | INTRON | NA | 0.3648 |
| rs141367147 | 33809461 | G | A | INTRON | NA | 0.0036 |
| rs10071596 | 33809758 | G | A | INTRON | NA | 0.2782 |
| rs80233200 | 33809798 | C | T | INTRON | NA | 0.1150 |
| . | 33809845 | G | C | INTRON | NA |  |
| rs1037101 | 33810076 | A | C | INTRON | NA | 0.2802 |
| rs717765 | 33810584 | C | T | INTRON | NA | 0.3698 |
| rs1445912 | 33810669 | G | A | INTRON | NA | 0.3335 |
| rs6891568 | 33810837 | A | G | INTRON | NA | 0.0224 |
| rs72741472 | 33811196 | C | T | INTRON | NA |  |
| rs1867719 | 33811418 | G | C | INTRON | NA | 0.1262 |
| rs10044427 | 33811670 | C | A | INTRON | NA | 0.1703 |
| . | 33811997 | G | A | INTRON | NA |  |
| rs10055310 | 33812113 | T | C | INTRON | NA | 0.2548 |
| rs1445911 | 33812568 | A | C | INTRON | NA | 0.3514 |
| rs4443421 | 33812770 | G | T | INTRON | NA | 0.3514 |
| rs75158920 | 33812958 | G | A | INTRON | NA | 0.2288 |
| rs77684540 | 33813143 | G | A | INTRON | NA | 0.0094 |
| rs62349806 | 33813364 | A | C | INTRON | NA | 0.3828 |
| rs2084124 | 33813435 | A | T | INTRON | NA | 0.3626 |
| rs2084123 | 33813608 | C | T | INTRON | NA | 0.3604 |
| rs10941092 | 33813994 | A | G | INTRON | NA | 0.3602 |
| rs7701400 | 33814173 | T | C | INTRON | NA | 0.1484 |
| rs11955017 | 33814207 | A | G | INTRON | NA | 0.3604 |
| rs11955019 | 33814228 | A | G | INTRON | NA | 0.3604 |
| rs10941093 | 33814388 | A | G | INTRON | NA | 0.1703 |
| rs62349807 | 33814399 | T | C | INTRON | NA | 0.3602 |
| . | 33814447 | A | C | INTRON | NA |  |
| rs34230132 | 33814479 | T | C | INTRON | NA | 0.3604 |
| rs72741475 | 33814705 | G | A | INTRON | NA | 0.1044 |
| rs10941095 | 33814862 | G | A | INTRON | NA | 0.3329 |
| . | 33815338 | C | T | INTRON | NA |  |
| rs984665 | 33815386 | T | A | INTRON | NA | 0.3496 |
| rs2197516 | 33815468 | T | C | INTRON | NA | 0.3620 |
| rs10941096 | 33815471 | T | G | INTRON | NA | 0.3620 |
| rs10805582 | 33815648 | A | G | INTRON | NA | 0.3620 |
| . | 33815992 | G | A | INTRON | NA |  |
| rs1445910 | 33816012 | C | G | INTRON | NA |  |
| rs12518063 | 33816335 | T | C | INTRON | NA | 0.0853 |
| rs190751145 | 33816336 | G | A | INTRON | NA | 0.0018 |
| rs895367 | 33816433 | T | C | INTRON | NA | 0.3646 |
| rs34511729 | 33816543 | C | G | INTRON | NA | 0.3163 |
| rs34936889 | 33816663 | C | T | INTRON | NA | 0.3664 |
| rs10941097 | 33816874 | T | A | INTRON | NA | 0.3508 |
| rs10941098 | 33816914 | A | C | INTRON | NA | 0.3343 |
| rs10941099 | 33816922 | G | C | INTRON | NA | 0.3433 |
| rs6895892 | 33816942 | A | T | INTRON | NA | 0.3894 |
| . | 33817054 | G | T | INTRON | NA |  |
| rs895366 | 33817078 | T | A | INTRON | NA | 0.4902 |
| rs895365 | 33817083 | T | C | INTRON | NA | 0.4910 |
| rs895364 | 33817174 | A | G | INTRON | NA | 0.4926 |
| rs1867718 | 33817339 | A | G | INTRON | NA | 0.4796 |
| rs72741480 | 33817367 | T | C | INTRON | NA | 0.0521 |
| rs1445909 | 33817593 | T | C | INTRON | NA | 0.3616 |
| rs10941100 | 33817833 | A | G | INTRON | NA | 0.0927 |
| rs2548037 | 33817865 | C | T | INTRON | NA | 0.1052 |
| rs2548036 | 33817901 | T | C | INTRON | NA | 0.3618 |
| rs10941101 | 33818021 | A | C | INTRON | NA | 0.2007 |
| rs2548035 | 33818151 | C | T | INTRON | NA | 0.3618 |
| rs13169685 | 33818371 | T | C | INTRON | NA | 0.0373 |
| rs59969290 | 33818413 | T | G | INTRON | NA | 0.2378 |
| rs56146616 | 33819043 | G | C | INTRON | NA | 0.2440 |
| rs2548033 | 33819287 | A | T | INTRON | NA | 0.4004 |
| rs4145601 | 33819981 | T | C | INTRON | NA | 0.2380 |
| rs12514368 | 33820006 | G | A | INTRON | NA | 0.2031 |
| rs12187521 | 33820123 | T | C | INTRON | NA | 0.1997 |
| rs12652514 | 33820355 | C | T | INTRON | NA | 0.2017 |
| rs2548032 | 33820448 | T | C | INTRON | NA | 0.3646 |
| rs10941102 | 33820504 | A | G | INTRON | NA | 0.0543 |
| rs2591732 | 33820587 | C | G | INTRON | NA |  |
| rs2548031 | 33820804 | A | G | INTRON | NA | 0.4002 |
| rs2548030 | 33820992 | G | A | INTRON | NA | 0.3536 |
| rs78904097 | 33821147 | C | T | INTRON | NA | 0.0174 |
| rs7718656 | 33821202 | C | T | INTRON | NA | 0.2378 |
| rs7719128 | 33821466 | C | T | INTRON | NA | 0.2378 |
| . | 33821520 | T | A | INTRON | NA |  |
| rs2591731 | 33821630 | C | T | INTRON | NA | 0.3538 |
| rs56270723 | 33821895 | C | T | INTRON | NA | 0.2380 |
| rs1374020 | 33822482 | T | C | INTRON | NA | 0.2378 |
| rs1445908 | 33822639 | T | C | INTRON | NA | 0.2161 |
| rs1374019 | 33822682 | T | A | INTRON | NA | 0.3860 |
| rs187153716 | 33822744 | C | T | INTRON | NA | 0.0020 |
| rs2591730 | 33822906 | A | C | INTRON | NA | 0.1440 |
| . | 33823147 | G | C | INTRON | NA |  |
| rs11741913 | 33823190 | C | T | INTRON | NA | 0.2015 |
| . | 33823400 | T | C | INTRON | NA |  |
| rs11952033 | 33823475 | G | A | INTRON | NA | 0.4974 |
| rs6872014 | 33823657 | T | C | INTRON | NA | 0.4928 |
| rs62349827 | 33823742 | C | T | INTRON | NA | 0.4493 |
| rs6451025 | 33823776 | G | A | INTRON | NA | 0.4982 |
| rs62349828 | 33823824 | G | T | INTRON | NA | 0.1947 |
| rs11749356 | 33824254 | A | G | INTRON | NA | 0.0324 |
| rs10941103 | 33824395 | C | T | INTRON | NA | 0.2770 |
| . | 33824429 | C | T | INTRON | NA |  |
| . | 33824670 | T | C | INTRON | NA |  |
| rs185163017 | 33824888 | C | G | INTRON | NA | 0.0014 |
| rs62349829 | 33824898 | G | A | INTRON | NA | 0.2686 |
| rs4866387 | 33825090 | G | A | INTRON | NA | 0.4776 |
| rs4866388 | 33825131 | C | T | INTRON | NA | 0.1779 |
| rs1862562 | 33825582 | A | T | INTRON | NA | 0.1138 |
| rs7445739 | 33826380 | C | G | INTRON | NA | 0.0260 |
| rs2548029 | 33826866 | C | G | INTRON | NA | 0.3373 |
| rs188567197 | 33826897 | G | C | INTRON | NA | 0.0028 |
| rs1423302 | 33827677 | T | C | INTRON | NA | 0.1344 |
| rs977842 | 33828203 | G | A | INTRON | NA | 0.3860 |
| rs3903442 | 33828593 | T | C | INTRON | NA | 0.4998 |
| rs2591727 | 33828707 | C | T | INTRON | NA | 0.1737 |
| rs7380826 | 33828835 | A | G | INTRON | NA | 0.0294 |
| rs2548026 | 33828944 | G | A | INTRON | NA | 0.4275 |
| rs55766742 | 33829069 | T | C | INTRON | NA | 0.1508 |
| rs6894469 | 33829264 | C | A | INTRON | NA | 0.1717 |
| rs2548025 | 33830565 | G | A | INTRON | NA | 0.1556 |
| rs961079 | 33830687 | A | T | INTRON | NA |  |
| rs6860534 | 33830765 | C | T | INTRON | NA | 0.4834 |
| rs7705765 | 33831013 | A | G | INTRON | NA | 0.0495 |
| rs2591726 | 33831019 | C | A | INTRON | NA | 0.4139 |
| rs961251 | 33831168 | A | G | INTRON | NA | 0.2474 |
| rs1363880 | 33831305 | A | G | INTRON | NA | 0.4559 |
| rs961252 | 33831355 | T | C | INTRON | NA | 0.2428 |
| rs2548023 | 33831973 | G | A | INTRON | NA | 0.4920 |
| rs2591724 | 33832015 | A | G | INTRON | NA | 0.4421 |
| rs6870893 | 33832169 | C | G | INTRON | NA | 0.3476 |
| rs1562246 | 33832234 | C | T | INTRON | NA | 0.0493 |
| rs1423300 | 33832433 | G | A | INTRON | NA | 0.4251 |
| rs2591723 | 33832958 | T | C | INTRON | NA | 0.2450 |
| rs1423299 | 33833140 | T | A | INTRON | NA | 0.2454 |
| rs2591722 | 33833462 | A | C | INTRON | NA | 0.2454 |
| rs77371652 | 33834092 | C | T | INTRON | NA | 0.0953 |
| rs62349832 | 33834178 | G | A | INTRON | NA | 0.0042 |
| rs2591721 | 33834257 | A | G | INTRON | NA | 0.2456 |
| rs141583521 | 33834545 | C | T | INTRON | NA | 0.0092 |
| rs2591720 | 33835062 | A | C | INTRON | NA | 0.2454 |
| rs113958724 | 33835091 | A | T | INTRON | NA | 0.0270 |
| rs62349833 | 33835174 | A | G | INTRON | NA | 0.0887 |
| rs1374017 | 33835353 | T | C | INTRON | NA | 0.2464 |
| . | 33835572 | A | G | INTRON | NA |  |
| rs2591719 | 33835643 | G | A | INTRON | NA |  |
| rs1445907 | 33835718 | C | T | INTRON | NA | 0.2374 |
| rs1363879 | 33836428 | G | C | INTRON | NA | 0.3119 |
| rs2591716 | 33836436 | G | C | INTRON | NA | 0.4109 |
| rs62349836 | 33837049 | T | C | INTRON | NA | 0.0192 |
| rs2548022 | 33838722 | A | G | INTRON | NA | 0.2200 |
| rs1465437 | 33839238 | C | T | INTRON | NA | 0.0935 |
| rs1465436 | 33839309 | T | C | INTRON | NA | 0.0931 |
| rs1465435 | 33839318 | G | A | INTRON | NA | 0.2198 |
| rs6881432 | 33839555 | C | T | INTRON | NA | 0.0823 |
| . | 33839918 | G | A | INTRON | NA |  |
| rs145326083 | 33840033 | G | A | INTRON | NA | 0.0208 |
| rs2548020 | 33842268 | A | G | INTRON | NA | 0.4369 |
| rs13184475 | 33842746 | G | C | INTRON | NA | 0.1741 |
| rs189974962 | 33843382 | C | T | INTRON | NA | 0.0066 |
| rs146674550 | 33843906 | G | T | INTRON | NA | 0.0004 |
| rs116074720 | 33844562 | C | A | INTRON | NA | 0.1530 |
| rs11738654 | 33844621 | C | T | INTRON | NA | 0.1743 |
| rs2591734 | 33844859 | T | C | INTRON | NA | 0.4077 |
| rs143790397 | 33844898 | G | C | INTRON | NA | 0.0935 |
| rs2548019 | 33844927 | A | C | INTRON | NA | 0.4367 |
| rs139318738 | 33844998 | C | T | INTRON | NA | 0.0160 |
| rs34928410 | 33845054 | C | T | INTRON | NA | 0.0769 |
| rs116491214 | 33845056 | C | T | INTRON | NA | 0.0056 |
| rs35147918 | 33845101 | G | A | INTRON | NA | 0.1743 |
| rs79762132 | 33845378 | G | A | INTRON | NA | 0.0931 |
| rs190118688 | 33846155 | C | T | INTRON | NA | 0.0004 |
| rs2591728 | 33846283 | G | A | INTRON | NA | 0.4371 |
| rs4866391 | 33846330 | T | C | INTRON | NA | 0.0823 |
| . | 33846751 | G | A | INTRON | NA |  |
| rs77483196 | 33847428 | T | C | INTRON | NA | 0.0949 |
| rs4866392 | 33847457 | G | A | INTRON | NA | 0.1743 |
| rs4866393 | 33847556 | C | T | INTRON | NA | 0.3345 |
| rs138287907 | 33847683 | G | A | INTRON | NA | 0.0048 |
| rs2441090 | 33847752 | G | A | INTRON | NA | 0.4371 |
| rs146694587 | 33847940 | G | A | INTRON | NA | 0.0006 |
| rs141453797 | 33848131 | C | A | INTRON | NA | 0.0108 |
| rs2017108 | 33848254 | G | A | INTRON | NA | 0.0949 |
| rs1966495 | 33848923 | A | G | INTRON | NA | 0.4073 |
| . | 33849442 | A | C | INTRON | NA |  |
| . | 33849465 | A | C | INTRON | NA |  |
| rs144145257 | 33849586 | C | T | INTRON | NA |  |
| rs144911072 | 33849611 | T | C | INTRON | NA |  |
| rs116424735 | 33849686 | T | C | INTRON | NA |  |
| rs147928694 | 33849826 | A | G | INTRON | NA | 0.0519 |
| rs62349858 | 33849959 | C | T | INTRON | NA | 0.3389 |
| . | 33850101 | T | C | INTRON | NA |  |
| rs1820137 | 33850404 | T | A | INTRON | NA | 0.4351 |
| rs149567494 | 33850595 | C | T | INTRON | NA | 0.0064 |
| rs16891832 | 33850646 | T | C | INTRON | NA | 0.1402 |
| . | 33850667 | G | A | INTRON | NA |  |
| . | 33850896 | T | C | INTRON | NA |  |
| . | 33850922 | G | A | INTRON | NA |  |
| rs72741496 | 33852528 | C | T | INTRON | NA | 0.0176 |
| . | 33852603 | G | C | INTRON | NA |  |
| rs192117305 | 33853188 | C | T | INTRON | NA | 0.0028 |
| rs17501324 | 33853341 | C | T | INTRON | NA | 0.0268 |
| . | 33853439 | C | T | INTRON | NA |  |
| rs16891840 | 33853753 | C | G | INTRON | NA | 0.0957 |
| rs149668741 | 33854210 | C | G | INTRON | NA | 0.0080 |
| rs13155761 | 33854740 | C | T | INTRON | NA | 0.1767 |
| rs1820136 | 33855980 | C | T | INTRON | NA | 0.3353 |
| rs1820135 | 33856049 | T | C | INTRON | NA | 0.4075 |
| rs140630233 | 33856128 | T | C | INTRON | NA | 0.0006 |
| rs116353537 | 33856281 | G | A | INTRON | NA | 0.0080 |
| rs116213943 | 33856527 | C | T | INTRON | NA | 0.0006 |
| rs7704760 | 33857302 | T | C | INTRON | NA | 0.3419 |
| . | 33858133 | A | T | INTRON | NA |  |
| rs1820134 | 33858228 | A | T | INTRON | NA | 0.3419 |
| rs13153094 | 33859045 | C | T | INTRON | NA | 0.1765 |
| rs114803348 | 33859404 | C | A | INTRON | NA | 0.0691 |
| rs2910635 | 33860263 | C | T | INTRON | NA | 0.4619 |
| rs3096133 | 33861457 | G | A | INTRON | NA | 0.4073 |
| . | 33862042 | A | G | INTRON | NA |  |
| rs4866395 | 33866241 | G | A | INTRON | NA | 0.3417 |
| rs74371619 | 33866463 | A | G | INTRON | NA | 0.0829 |
| rs62349859 | 33866777 | A | G | INTRON | NA | 0.1585 |
| . | 33866876 | A | C | INTRON | NA |  |
| rs10941108 | 33867419 | T | C | INTRON | NA | 0.1651 |
| rs34593012 | 33867593 | A | T | INTRON | NA | 0.0106 |
| rs55737732 | 33870175 | C | A | INTRON | NA | 0.1382 |
| rs7712870 | 33870868 | T | A | INTRON | NA | 0.1765 |
| rs77110833 | 33871090 | G | C | INTRON | NA | 0.0082 |
| rs76641739 | 33871257 | T | C | INTRON | NA | 0.0160 |
| rs75989721 | 33871466 | T | C | INTRON | NA | 0.0859 |
| rs73074808 | 33871850 | A | G | INTRON | NA | 0.1589 |
| . | 33872158 | T | C | INTRON | NA |  |
| rs12656358 | 33872343 | C | T | INTRON | NA | 0.1765 |
| . | 33872567 | G | A | INTRON | NA |  |
| rs4866397 | 33874324 | A | T | INTRON | NA | 0.3417 |
| rs7379743 | 33875121 | T | C | INTRON | NA | 0.3373 |
| rs115615091 | 33875338 | T | C | INTRON | NA | 0.0072 |
| . | 33875361 | G | A | INTRON | NA |  |
| . | 33876128 | G | A | INTRON | NA |  |
| rs1582561 | 33876331 | A | C | INTRON | NA | 0.3776 |
| rs12522657 | 33876964 | G | A | INTRON | NA | 0.1651 |
| rs2113109 | 33877007 | A | G | INTRON | NA | 0.4237 |
| rs7709003 | 33877451 | C | A | INTRON | NA | 0.4073 |
| rs7729099 | 33877537 | T | C | INTRON | NA | 0.4071 |
| rs6894957 | 33878130 | T | A | INTRON | NA | 0.4071 |
| rs13160471 | 33878205 | C | T | INTRON | NA | 0.2115 |
| rs6859977 | 33879045 | T | C | INTRON | NA | 0.4000 |
| rs1423692 | 33879407 | G | A | INTRON | NA | 0.0697 |
| rs2277010 | 33879744 | A | G | INTRON | NA | 0.4281 |
| rs1364044 | 33879792 | C | T | INTRON | NA | 0.3706 |
| . | 33880079 | C | T | INTRON | NA |  |
| . | 33880082 | G | C | INTRON | NA |  |
| rs7443937 | 33880679 | T | C | INTRON | NA | 0.3301 |
| rs75486147 | 33880960 | C | G | INTRON | NA | 0.1046 |
| rs80107811 | 33882018 | G | A | INTRON | NA | 0.0002 |
| rs13182017 | 33882284 | C | T | INTRON | NA | 0.2111 |
| rs148294586 | 33882765 | C | T | INTRON | NA | 0.0048 |
| rs4049255 | 33883233 | T | C | INTRON | NA | 0.3307 |
| rs72743306 | 33883800 | G | C | INTRON | NA | 0.0114 |
| rs4866398 | 33883876 | C | T | INTRON | NA | 0.1048 |
| rs6451031 | 33884127 | T | C | INTRON | NA | 0.4329 |
| rs75810312 | 33884217 | C | T | INTRON | NA | 0.0062 |
| . | 33884255 | T | C | INTRON | NA |  |
| rs62352460 | 33884516 | C | T | INTRON | NA | 0.0182 |
| rs6451032 | 33885273 | A | T | INTRON | NA | 0.0100 |
| . | 33885336 | G | A | INTRON | NA |  |
| rs115828027 | 33885529 | G | A | INTRON | NA | 0.0154 |
| rs2877341 | 33885860 | C | A | INTRON | NA | 0.1126 |
| rs7447613 | 33885945 | A | C | INTRON | NA | 0.4730 |
| rs6414860 | 33886719 | T | C | INTRON | NA | 0.3890 |
| rs13358296 | 33887319 | T | A | INTRON | NA | 0.1532 |
| rs4866399 | 33887419 | G | A | INTRON | NA | 0.2452 |
| rs62352461 | 33887549 | T | C | INTRON | NA | 0.1364 |
| . | 33887621 | G | A | INTRON | NA |  |
| rs113562753 | 33888055 | G | A | INTRON | NA | 0.1126 |
| . | 33888973 | A | C | INTRON | NA |  |
| rs16891881 | 33889079 | G | A | INTRON | NA | 0.1370 |
| rs7378990 | 33889275 | G | C | INTRON | NA | 0.2456 |
| rs12654089 | 33889628 | T | C | INTRON | NA | 0.2456 |
| rs10050861 | 33889645 | G | C | INTRON | NA | 0.3201 |
| rs12519294 | 33890051 | A | G | INTRON | NA | 0.4696 |
| rs113034428 | 33890340 | T | C | INTRON | NA | 0.0058 |
| rs889324 | 33891581 | C | T | INTRON | NA | 0.1318 |
| rs140486982 | 33891894 | G | A | NON_SYNONYMOUS_CODING | 0 | 0.0002 |
| rs7443383 | 33893952 | C | T | UPSTREAM | NA | 0.0082 |
| rs10521009 | 33894764 | G | A | UPSTREAM | NA | 0.4301 |
| rs116150065 | 33895241 | T | C | UPSTREAM | NA | 0.0172 |
| rs10521010 | 33895326 | T | C | UPSTREAM | NA | 0.1566 |
| rs13357703 | 33895453 | G | C | UPSTREAM | NA | 0.2196 |
| rs10521011 | 33895956 | T | C | UPSTREAM | NA | 0.0172 |
| rs59839466 | 33896604 | A | G | UPSTREAM | NA | 0.0984 |
| rs114297473 | 33896739 | T | C | UPSTREAM | NA | 0.0030 |
| rs62352462 | 33896793 | A | G | UPSTREAM | NA | 0.2198 |
| rs144948069 | 33896906 | G | T | UPSTREAM | NA | 0.0068 |
