# Supplementary material for: ADAMTS12, a new candidate gene for pediatric stroke
Source: PLoS One. 2020 Aug 20;15(8):e0237928. doi: 10.1371/journal.pone.0237928 (PMC7446847; doi:10.1371/journal.pone.0237928)
Supplement: S3 Table — Results of the SDT association analysis in 48 discordant sib-pairs for the non-synonymous variants in ADAMTS2 and ADAMTS12. (DOCX) [file pone.0237928.s004.docx]

**S3 Table.** **Sibship Disequilibrium Test (SDT) for *ADAMTS2* and *ADAMTS12* variants.** Results of the SDT association analysis in 48 discordant sib-pairs for the non-synonymous variants in *ADAMTS2* and *ADAMTS12*.

| SNP | POS_hg19 | SDT_PVAL | REF | ALT | AF | Gene | Protein_Domain |
| --- | --- | --- | --- | --- | --- | --- | --- |
| chr5:179207680_C/T | 178634681 | 0.317~~3105~~ | C | T | 0.022 | ADAMTS2 | Peptidase_M12B |
| rs1054480 | 178540975 | 0.317~~3105~~ | G | A | 0.3 | ADAMTS2 | no_domain |
| rs11750821 | 178634683 | 0.179~~7125~~ | C | T | 0.091 | ADAMTS2 | Peptidase_M12B |
| rs139658049 | 178578135 | NA | C | T | 0.011 | ADAMTS2 | no_domain |
| rs35372714 | 178563002 | 0.317~~3105~~ | C | T | 0.016 | ADAMTS2 | no_domain |
| rs35445112 | 178555097 | 0.317~~3105~~ | C | T | 0.016 | ADAMTS2 | ADAM_spacer1 |
| rs398829 | 178634672 | 0.205~~9032~~ | C | T | 0.261 | ADAMTS2 | Peptidase_M12B |
| rs59567206 | 178634704 | 0.317~~3105~~ | T | C | 0.006024 | ADAMTS2 | Peptidase_M12B |
| rs112196098 | 33549350 | 0.157~~2992~~ | C | G | 0.032 | ADAMTS12 | Thrombospondin_1_Repeat |
| rs140486982 | 33891894 | 0.317~~3105~~ | G | A | 0.005263 | ADAMTS12 | Sigp |
| rs25754 | 33535060 | 0.345~~7786~~ | G | A | 0.395 | ADAMTS12 | Thrombospondin_1_Repeat |
| rs3813474 | 33576602 | 0.527~~0893~~ | A | G | 0.105 | ADAMTS12 | no_domain |
| rs61753559 | 33546207 | NA | T | C | 0.021 | ADAMTS12 | Thrombospondin_1_Repeat |
| rs77581578 | 33549374 | 0.157~~2992~~ | G | T | 0.032 | ADAMTS12 | Thrombospondin_1_Repeat |
